# The Role of Fat‐Free Mass in Weight‐Related Complications: A Rapid Review and Meta‐Analysis

**DOI:** 10.1111/obr.70202

**Published:** 2026-07-21

**Authors:** Tadesse Asmamaw Dejenie, Paul P. Fahey, Haider Mannan, Mohammad Ariya, Evan Atlantis

**Affiliations:** ^1^ School of Health Sciences Western Sydney University Campbelltown New South Wales Australia; ^2^ Department of Biochemistry, College of Medicine and Health Sciences University of Gondar Gondar Ethiopia; ^3^ Translational Health Research Institute Western Sydney University Campbelltown New South Wales Australia

**Keywords:** body composition, FFM, obesity, weight‐related complications

## Abstract

**Background:**

This review synthesized evidence on the association between fat‐free mass (FFM) metrics and weight‐related metabolic complications in adults with overweight or obesity.

**Methods:**

A systematic search of Medline, Embase, Web of Science, and Google Scholar (inception to October 2024) identified 32 observational studies involving 138,935 participants. Random‐effects meta‐analyses were conducted for type 2 diabetes (T2DM, 5 studies, *n* = 41,235), metabolic syndrome (MetS, 3 studies, *n* = 2832), cardiovascular disease (CVD, 3 studies, *n* = 8980), and non‐alcoholic fatty liver disease (NAFLD, 3 studies, *n* = 4570). Heterogeneity was assessed using I^2^. Sensitivity analyses stratification by FFM metric type were performed where feasible.

**Results:**

Low FFM metrics were significantly associated with increased risk of T2DM (pooled RR = 1.46, 95% CI 1.11–1.91), MetS (OR = 4.48, 95% CI 2.18–9.22), CVD (RR = 2.03, 95% CI 1.27–3.26), and NAFLD (RR = 2.24, 95% CI 1.52–3.32). High heterogeneity was observed for T2DM (*I* ^2^ = 90.9%) and MetS (*I* ^2^ = 79.8%); however, sex‐stratified sensitivity analyses eliminated heterogeneity for T2DM (*I* ^2^ = 0%). Narrative synthesis of remaining studies generally supported these findings, though associations varied according to FFM metric type.

**Conclusions:**

Low FFM‐related metrics were associated with increased risk of major weight‐related metabolic complications, although the strength and consistency of associations differed by FFM metric type.

**Abbreviations**
ASM: Appendicular skeletal muscle mass
BIA: Bioelectrical impedance analysis
BMI: Body mass index
CI: Confidence interval
COPD: Chronic obstructive pulmonary disease
CVD: Cardiovascular disease
DXA: Dual‐energy X‐ray absorptiometry
FFM: Fat‐free mass
GFR: Glomerular filtration rate
HDL: High‐density lipoprotein
HOMA‐IR: Homeostatic model assessment‐insulin resistance
HR: Hazard ratio
JBI: Joanna Briggs Institute
MetS: Metabolic syndrome
MOOSE: Meta‐analysis Of Observational Studies in Epidemiology
NAFLD: Non‐alcoholic fatty liver disease
OR: Odds ratio
PICOS: Population, Interventions, Comparators, Outcomes, and Study designs
PRISMA: Preferred Reporting Items for Systematic Reviews and Meta‐Analyses Protocols
RR: Risk ratio
SMI: Skeletal muscle mass index
T2DM: Type 2 diabetes mellitus
QUICKI: Quantitative insulin sensitivity check index

## 1 Introduction

Obesity is a major global health issue that increases the risk of various chronic diseases, including cardiovascular diseases (CVDs), type 2 diabetes (T2DM), metabolic syndrome (MetS), and certain cancers [1, 2, 3, 4]. Additionally, obesity imposes substantial economic burdens through direct health care costs and lost productivity. Direct costs alone account for a significant portion of healthcare expenditure, while indirect costs, such as lost productivity, further exacerbate the financial burden [5, 6, 7, 8, 9]. Given the growing prevalence of obesity and its associated complications, there is an urgent need to better understand factors contributing to obesity‐related health risks and identify strategies that mitigate these complications [10, 11, 12].

Although excess adiposity is widely recognized as a key contributing factor to weight‐related complications, the role of fat‐free mass (FFM) is more complex. FFM is frequently used as a practical proxy for skeletal muscle mass (SMM) in scientific literature [13, 14]. However, studies report FFM using heterogeneous metrics, including absolute FFM (kg), height‐adjusted FFM (kg/m^2^), percentage FFM, and FFM relative to body weight or BMI [15, 16]. These metrics are not interchangeable and may reflect distinct physiological constructs. Relative measures such as FFM percentage or FFM/weight are mathematically influenced by adiposity, whereas height‐adjusted FFM measures may better reflect lean tissue quantity independent of fat mass [17]. Consequently, associations reported using relative FFM metrics may partly reflect changes in adiposity rather than lean tissue itself.

Prior observational studies suggested that lower FFM‐related metrics are associated with increased risk of insulin resistance, metabolic syndrome, and functional impairments [18, 19, 20, 21]. Conversely, higher FFM‐related measures have been associated with more favorable metabolic profiles, including improved insulin sensitivity, lower triglyceride and low‐density lipoprotein cholesterol levels, and higher high‐density lipoprotein (HDL) cholesterol levels [22, 23]. Randomized controlled trials further support the metabolic importance of muscle‐preserving interventions, with resistance training shown to improve metabolic profiles and overall health outcomes [24, 25]. The proposed mechanisms linking lower FFM to adverse metabolic health include impaired glucose utilization, reduced energy expenditure, excessive fat storage, and reduced physical functionality [26, 27]. However, the relationship between FFM and metabolic health is not uniformly protective. Several studies have reported adverse or null associations. For example, in sedentary postmenopausal women, higher appendicular lean mass was associated with greater insulin resistance [28]. Similarly, among women with polycystic ovary syndrome, lean mass correlated directly with HOMA‐IR, an insulin resistance marker [29]. In a large cohort of older adults, total lean mass showed no significant association with incident diabetes after adjustment for visceral fat [30]. Additionally, one longitudinal study found that annual increases in FFM were associated with higher incidence of hypertension [31]. Collectively, these findings demonstrate substantial heterogeneity in reported associations between FFM and metabolic health, potentially reflecting differences in FFM metrics, study population, or analytical approaches. This heterogeneity highlights the need for a comprehensive synthesis of the evidence to clarify the role of FFM‐related measures in weight‐related complications.

Therefore, this review aimed to evaluate associations between FFM‐related metrics and weight‐related complications in adults with overweight or obesity and to clarify their potential role in metabolic health.

## 2 Methods

### 2.1 Protocol and Registration

We developed the review protocol based on Joanna Briggs Institute (JBI) methodology guidelines [32], but it was not prospectively registered in PROSPERO or any other databases. The review was reported in accordance with the Preferred Reporting Items for Systematic Reviews and Meta‐Analyses Protocols (PRISMA) guidelines [33]. Additionally, we followed the reporting standards of the Meta‐analysis Of Observational Studies in Epidemiology (MOOSE) guidelines [34].

### 2.2 Eligibility Criteria

Eligibility criteria were defined using the PICOS framework [35]. The population included adults aged ≥ 18 years with overweight or obesity (BMI ≥ 25 kg/m^2^), as well as mixed‐BMI studies reporting stratified analyses for this subgroup. Eligible exposures included measures of FFM, such as absolute FFM (kg), height‐adjusted FFM (kg/m^2^), percentage FFM, FFM relative to BMI, skeletal muscle mass, appendicular lean mass, or related derived indices. Comparators were defined as higher versus lower FFM using study‐defined cutoffs (tertiles, quartiles, or predefined sarcopenia thresholds). Primary outcomes comprised T2DM, CVDs, MetS, and non‐alcoholic fatty liver disease, which were quantitatively synthesized in the meta‐analysis. Secondary outcomes synthesized narratively included hypertension, cardiorespiratory fitness, chronic obstructive pulmonary disease, and chronic kidney disease. Eligible study designs included cross‐sectional and cohort studies. Case reports, case series, reviews, editorials, conference abstracts, animal studies, and non‐English publications were excluded.

### 2.3 Search Strategy and Information Sources

In consultation with a university librarian, we employed a systematic search approach using selected keywords and phrases within titles and abstracts across databases, including Medline via Ovid, Embase via Ovid, Web of Science, and Google Scholar. The search strategy was designed for each database using Boolean operators and algorithms (Supplementary Table S1) and was informed by both a subject matter expert (EA) and an academic librarian, ensuring a comprehensive and effective search process. We included only original studies published in English and involving adult populations, with no restrictions on geographical location or publication date. The identified studies were exported to the Endnote 21 reference manager, where duplicate studies were removed.

Study selection was conducted in two phases according to the predefined eligibility criteria. Two authors (TAD and EA) independently screened titles and abstracts, with excellent inter‐reviewer agreement (κ = 0.89, 95% CI 0.84–0.94). Agreement during full‐text screening was also high (κ = 0.92, 95% CI 0.87–0.97). Disagreements were resolved through discussion with a third author (MA). Additionally, we reviewed the reference lists from included studies to identify further relevant articles and contacted the corresponding authors when additional data were needed. Justification for excluding studies identified in the second screening is provided in Supplementary Table S2.

### 2.4 Data Extraction and Risk of Bias Assessment

Two authors (TAD and EA) independently extracted key study characteristics, including study design, population, publication year, setting, independent variables, outcomes, statistical analysis, and key findings with author conclusions. Any discrepancies were resolved through discussion between these two reviewers and other members of the research team. To account for potential confounders, we prioritized the most adjusted effect estimates reported in the included studies. Moreover, we evaluated the methodological quality of the studies using the standardized critical appraisal tools provided by the JBI Meta‐Analysis of Statistics Assessment and Review Instrument [36].

### 2.5 Definition of Exposure Measures

Included studies reported heterogeneous FFM metrics. These measures were categorized as follows: (1) absolute or height‐adjusted FFM measures, including total FFM, lean mass, appendicular skeletal muscle mass (ASM; kg), FFM index (FFMI; kg/m^2^), and other height‐adjusted skeletal muscle mass indices; (2) relative or adiposity‐adjusted FFM measures, including FFM/weight, FFM/BMI, or percentage FFM, and other skeletal muscle mass indices standardized to body weight or BMI.

We acknowledge that these metrics are not interchangeable and may reflect different aspects of body composition. Ratio‐based or adiposity‐adjusted measures such as FFM percentage or FFM/BMI may be substantially influenced by adiposity, whereas absolute or height‐adjusted measures more directly reflect lean tissue quantity independent of adiposity. For consistency, we used FFM metrics as an umbrella term encompassing heterogeneous lean mass measures reported across studies.

For primary analyses, we pooled studies according to the original exposure definitions reported by the individual studies because of the limited number of eligible studies available for each outcome. Low FFM was therefore defined according to the original study‐specific operationalization (e.g., lowest tertile/quartile or below study‐defined cutoffs). To further assess whether body composition metric choice influenced findings, we conducted post hoc sensitivity analyses stratified by metric type: Type A (absolute FFM or height‐adjusted measures) and Type B (relative or adiposity‐adjusted FFM measures such as FFM/weight or FFM/BMI), where sufficient studies were available.

### 2.6 Effect Estimate Standardization

To improve comparability across studies, we applied the following standardization rules to all effect estimates prior to pooling.

Effect measure standardization: Studies were included in the meta‐analyses when effect estimates were reported as relative measures of association with corresponding 95% confidence intervals. For some outcomes (e.g., CVD and NAFLD), different effect estimates were reported across studies. In these cases, studies reporting hazard ratios (HRs) were excluded from the primary pooled analysis, and separated sensitivity analyses restricted to studies reporting odds ratios (ORs) were conducted to assess the robustness of findings.

Reference‐category standardization: For studies that used the lowest FFM category as the reference groups, effect estimates and corresponding 95% confidence intervals were mathematically inverted to standardize all pooled estimates to represent the relative risk associated with lower FFM exposure compared with higher FFM exposure. This ensured consistent exposure directionality across pooled analyses.

Subgroup combination: Where studies reported subgroup‐specific results separately (e.g., by sex), subgroup effect estimates were first combined using a fixed‐effect meta‐analysis model in accordance with the Cochrane Handbook guidelines [37], before pooling with results from other studies. The combined subgroup effect estimate, and variances were calculated using the inverse‐variance method.

Log transformation: All effect estimates (RR, OR, HR) were logarithmically transformed prior to pooling to stabilize variance and normalize the sampling distributions. Pooled effect estimates were subsequently back‐transformed (exponentiated) for presentation in forest plots.

Unit of analysis: Studies reporting associations per unit increase in FFM measures (e.g., per 1 kg/m^2^ increase in FFMI) were treated as continuous effect estimates, whereas studies reporting categorical comparisons (e.g., lowest vs. highest tertile) were analyzed as categorical effect estimates. No direct harmonization between continuous and categorical effect estimates was performed.

### 2.7 Meta‐Analytic Procedures

Once reference categories were standardized and subgroups combined, we conducted random‐effects meta‐analyses (DerSimonian–Laird method) for outcomes reported in three or more studies. Findings are presented as forest plots showing results from each study, study weights, and the pooled effect estimates with corresponding 95% CIs. Heterogeneity statistics (*I* ^2^, τ^*2*^) and the Q‐statistic test for evidence of heterogeneity are annotated onto each forest plot.

To assess the robustness of the pooled estimates and explore potential sources of heterogeneity, we performed sensitivity analyses using a leave‐one‐out approach, in which the analysis was repeated after sequentially removing each individual study to evaluate its influence on the overall effect estimate and heterogeneity. We also conducted subgroup analyses based on FFM measurement techniques (DEXA vs. BIA) and sex‐specific stratifications where available. These analyses enabled us to determine whether the main findings were consistent across different study subsets and to identify potential sources of heterogeneity.

We do not report Egger's test for publication bias as the number of pooled studies (*k* < 10) is insufficient for reliable estimation. All statistical analyses were conducted using the metafor package (version 3.8‐1) in R (version 4.2.0).

## 3 Result

### 3.1 Study Selection

**FIGURE 1:**
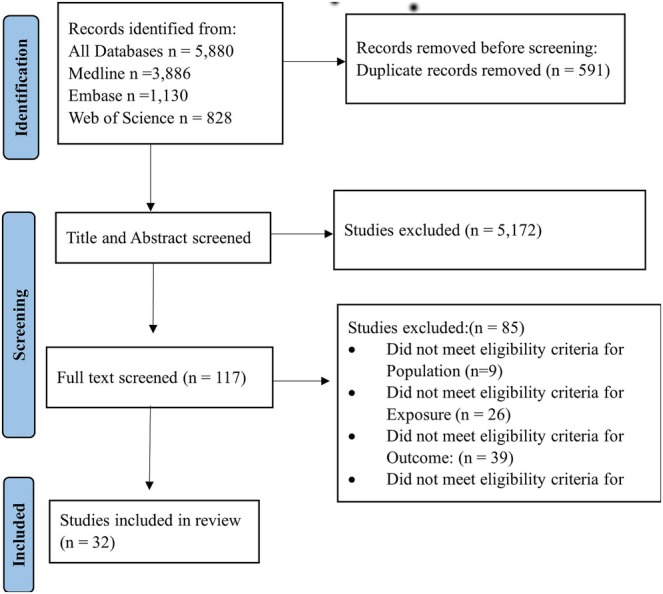
PRISMA flow diagram of study selection.

Through a search of selective academic databases, we identified 5880 studies. After removing 591 duplicates, 5289 articles were retrieved for title and abstract screening. During this process, 5172 articles were excluded, leaving 117 articles for full‐text screening. In the full text screening phase, 85 articles were excluded for not meeting the inclusion criteria, resulting in 32 articles being included in this study (Figure 1).

### 3.2 Study Characteristics

A detailed summary of the study characteristics is provided in Supplementary Table S3. We included 32 observational studies investigating the role of FFM in weight‐related complications. Of these, 21 were cross‐sectional studies [27, 28, 29, 38, 39, 40, 41, 42, 43, 44, 45, 46, 47, 48, 49, 50, 51, 52, 53, 54, 55] and 11 were cohort studies [19, 26, 30, 31, 56, 57, 58, 59, 60, 61, 62], with follow‐up periods ranging from 1 to 17 years. These studies were conducted across various regions: nine from South Korea [19, 38, 40, 42, 43, 48, 54, 56, 59], seven from the United States [29, 30, 50, 51, 52, 53, 58], three from Australia [46, 55], two each from the Netherlands [44, 60] and Greece [49, 61], and one from each of the remaining countries: Germany [31], Canada [28], Denmark [57], Italy [41], Spain [45], Taiwan [26], Brazil [39], Turkey [47], and Thailand [27]. Sample sizes ranged from 39 to 127,616 participants and ages from 18 to 82 years. Several studies specifically focused on older adults, including those aged 60 years and above [38], 70 years and above [43, 49], and 70–79 years [30], while one study included participants aged 21–82 years [31]. Twenty‐six studies [19, 26, 27, 29, 30, 31, 38, 39, 40, 41, 42, 43, 44, 45, 46, 47, 48, 50, 51, 52, 53, 54, 57, 58, 60, 61] included both sexes, three focused exclusively on women [28, 29, 49], and three included only men [38, 55, 62]. Fifteen studies reported mean BMIs within the overweight range (25–30 kg/m^2^) [19, 38, 43, 46, 48, 49, 50, 51, 52, 54, 56, 57, 58, 59, 62], six studies reported within the obesity range (30–35 kg/m^2^) [28, 29, 31, 41, 44, 53], and one study reported a mean BMI exceeding 40 kg/m^2^ [26]. The other 10 studies reported the prevalence of overweight and obesity as a percentage of study population [27, 30, 39, 40, 42, 45, 47, 55, 60, 61].

The most used methods for assessing body composition were DEXA in 16 (50%) studies and bioelectrical impedance analysis (BIA) in 14 (44%) studies. One used computed tomography imaging [60], and another used 24‐h urinary creatinine excretion rate [40]. The reported measures of FFM varied across studies: seven studies reported lean mass [30, 46, 47, 50, 53, 57, 62], three reported FFM [31, 45, 58], and two reported appendicular skeletal muscle mass (ASM) [42, 43]. Several studies also reported indices derived from these measures, including FFM index [44], skeletal muscle mass index (SMI) [40, 54], and lean mass index [49]. Thirteen studies reported the combination of two or more of these measures [19, 26, 27, 28, 29, 38, 39, 41, 48, 51, 52, 59, 61]. Regarding outcomes, we identified 12 studies that explored the role of FFM in risk of T2DM [19, 26, 27, 28, 29, 30, 41, 50, 51, 58, 60, 62], eight studies on cardiovascular risks [42, 45, 46, 48, 49, 54, 57, 61], four on metabolic syndrome [39, 43, 55, 59], three on non‐alcoholic fatty liver disease (NAFLD) [40, 52, 56], two on cardiorespiratory fitness [38, 53], and one study each on hypertension [31], chronic obstructive pulmonary disease [44], and chronic kidney disease [47].

### 3.3 Risk of Bias Within Studies

We present the results of our quality assessment for each study in the Supplemental Material (Supplementary Table S4). All 11 cohort studies met at least 82% of the quality criteria [19, 26, 30, 31, 40, 57, 58, 59, 60, 61, 62]. Of the 21 cross‐sectional studies, 17 studies met 100% quality criteria [27, 29, 38, 39, 40, 42, 43, 45, 47, 48, 49, 50, 51, 52, 53, 54, 55] and the remaining four studies met at least 75% quality items [28, 41, 44, 46].

### 3.4 Synthesis With Meta‐Analysis

Meta‐analyses were conducted for four major complications, including T2DM (5 studies [19, 30, 51, 58, 60]; *n* = 41,850), MetS (3 studies [39, 43, 55]; *n* = 2939), CVD risk (3 studies [42, 54, 61]; *n* = 8980), and NAFLD (3 studies [40, 52, 56]; *n* = 4570). All pooled analyses demonstrated significantly increased risks of weight‐related complications in individuals with lower FFM metrics. Meta‐analyses for T2DM and MetS showed substantial heterogeneity between studies. Stratified post hoc sensitivity analyses, where feasible, revealed that both effect estimates and heterogeneity varied according to FFM metric type (Type A: absolute or height‐adjusted FFM measures; Type B: relative or adiposity‐adjusted FFM measures) (Supplementary Table S5). However, no meaningful differences in effect estimates and heterogeneity were observed in sex‐specific subgroup analyses. Detailed findings for each outcome are presented below, with additional analyses provided in the Supplemental Materials.

### 3.5 T2DM

**FIGURE 2:**
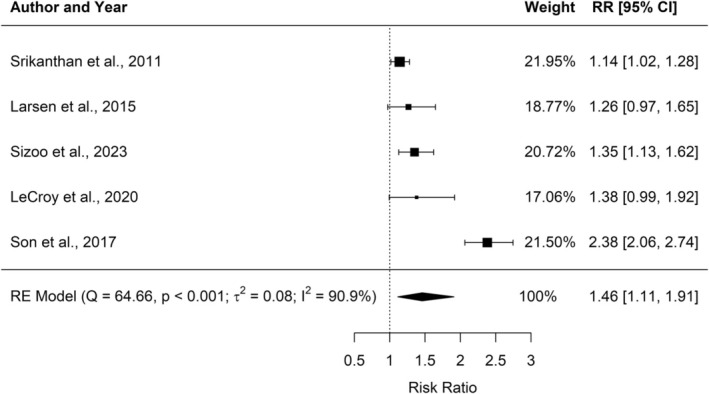
Forest plot illustrating the association between low FFM and risk of T2DM.

Meta‐analysis of the five eligible studies [19, 30, 51, 58, 60] demonstrated a statistically significant association between low FFM and an increased risk of developing T2DM (pooled RR = 1.46, 95% CI 1.11–1.91), with considerable heterogeneity (*Q =* 64.66, *p* < 0.001; *τ* ^*2*^ = 0.08; *I* ^2^ = 90.9%) (Figure 2). Despite the substantial heterogeneity, all point estimates indicated a positive association, consistently favoring the exposed group.

In sensitivity analyses, exclusion of one study conducted in older participants (70–79 years) [30] did not meaningfully alter the pooled effect estimate (RR = 1.50, 95% CI 1.08–2.09), although heterogeneity remained high (*Q =* 63.36, *p* < 0.001; *τ* ^*2*^ = 0.10, *I* ^2^ = 93.4%) (Supplementary Figure S1). Exclusion of another study [19] with a comparatively large effect estimate resulted in a modest reduction in the pooled association (RR = 1.24, 95% CI 1.11–1.38), along with a substantial reduction in heterogeneity (*Q* = 3.14, *p* = 0.371; *τ* ^*2*^ = 0.00, *I* ^2^ = 25.3%) (Supplementary Figure S2).

In sex‐stratified subgroup analyses, sex‐specific estimates from studies [30, 60] were pooled separately for males and females. Male specific analyses yielded a pooled RR of 1.23 (95% CI 1.00–1.52), with low heterogeneity (*Q* = 0.11, *p* = 0.736; *τ* ^*2*^ = 0.00, *0* ^2^ = 0.0%). Female‐specific analyses demonstrated a comparable association (RR = 1.42, 95% CI 1.15–1.76), with no observed heterogeneity (*Q* = 0.00, *p* = 0.978; *τ* ^*2*^ = 0.00, *I* ^2^ = 0.0%) (Supplementary Figure S3). The influential outlier study did not report sex‐specific results and was therefore not included in these subgroup analyses.

In analyses stratified by body composition measurement method, studies using BIA [19, 51, 58] yielded slightly higher but nonsignificant pooled effect estimate (RR = 1.56, 95% CI 0.99–2.44), with considerable heterogeneity (*Q* = 62.1, *p* = 0.0; *τ* ^*2*^ = 0.1, *I* ^2^ = 95.3%) (Supplementary Figure S4). There were insufficient studies using DXA [30] to permit pooled analysis. Restricting analyses to cohort studies [19, 30, 58, 60], the association remained statistically significant (RR = 1.56, 95% CI 1.15–2.12), although substantial heterogeneity persisted (*Q* = 33.50, *p* < 0.001; *τ* ^*2*^ = 0.08, *I* ^2^ = 87.9%) (Supplementary Figure S5).

In post hoc sensitivity analyses stratifying by metric types, analyses restricting to Type A studies [30, 60] (absolute FFM or height‐adjusted FFM metrics) showed a significant association between lower FFM metrics and T2DM risk (RR = 1.32, 95% CI 1.14–1.54), with no evidence of heterogeneity (*Q* = 0.16, *p* = 0.687; *τ* ^*2*^ = 0.00, *I* ^2^ = 00%) (Supplementary Figure S6). In contrast, Type B studies [19, 51, 58] (weight‐relative FFM metrics, including FFM/weight or FFM/BMI) yielded a larger pooled effect estimate (RR = 1.56, 95% CI 0.99–2.44), although the association was not significant and substantial heterogeneity was observed (*Q* = 62.11, *p* < 0.001; *τ* ^*2*^ = 0.15, *I* ^2^ = 95.3%). Overall, the direction of the association remained consistent across FFM metric types, although effect estimates, statistical significance, and between‐study heterogeneity differed according to the metric used.

### 3.6 MetS

**FIGURE 3:**
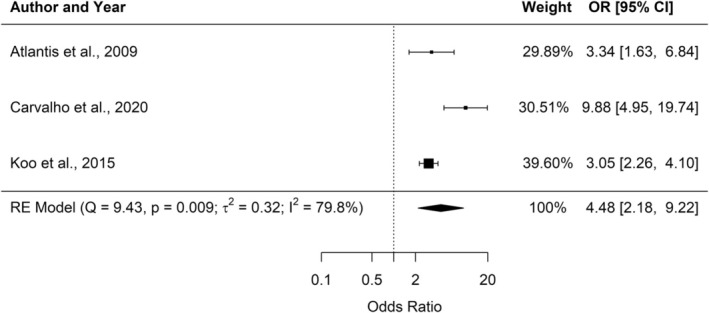
Forest plot illustrating the association between low FFM and MetS.

Meta‐analysis of three studies [39, 43, 55] indicated that low FFM was significantly associated with higher odds of MetS, with a pooled effect estimate of OR = 4.48 (95% CI 2.18–9.22) (Figure 3). However, substantial heterogeneity was observed across studies (*Q* = 9.43, *p* = 0.009; *τ* ^*2*^ = 0.32, *I* ^2^ = 79.8%).

Leave‐one‐out sensitivity analysis demonstrated that exclusion of one influential study [39] resulted in a pooled OR of 3.09 (95% CI 2.35–4.06), with no residual heterogeneity (*Q* = 0.05, *p* = 0.816; *τ* ^*2*^ = 0.00, *I* ^2^ = 0.0%) (Supplementary Figure S7), while retaining statistical significance. However, the omission of other individual studies did not meaningfully reduce heterogeneity.

Sex‐stratified analyses revealed directionally similar associations. Among studies restricted to male participants, the pooled effect estimate was OR = 5.27 (95% CI: 2.13–13.00), with substantial heterogeneity (*Q* = 5.72, *p* = 0.057; *τ* ^*2*^ = 0.46, *I* ^2^ = 76.1%). Among studies restricted to female participants, the pooled effect estimate was OR = 4.20 (95% CI 1.45–12.20), also with substantial heterogeneity (*Q* = 5.70, *p* = 0.017; *τ* ^*2*^ = 0.49, *I* ^2^ = 82.4%) (Supplementary Figure S8).

### 3.7 CVD Risk

**FIGURE 4:**
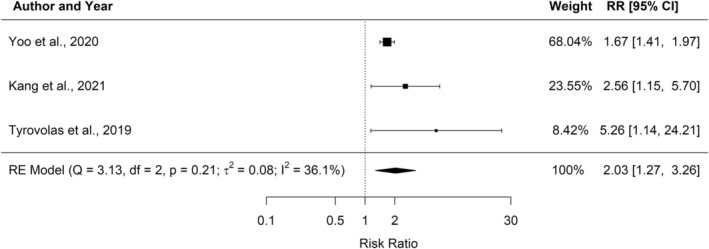
Forest plot illustrating the association between low FFM and risk of CVD.

Meta‐analysis of 3 studies [42, 54, 61] found that low FFM metric was significantly associated with more than twofold increased risk of CVD (pooled RR = 2.03; 95% CI 1.27–3.26), with low to moderate heterogeneity (*Q* = 3.13, *p* = 0.21; *τ* ^2^ = 0.08, *I* ^2^ = 36.1%) (Figure 4). Replacing the carotid plaque outcome with intima–media thickness in one study [42], which reported two indicators of subclinical atherosclerosis, yielded a similar pooled effect estimate (RR = 1.99; 95% CI 1.32–3.00) and heterogeneity level (*Q* = 3.33, *p* = 0.19; *τ* ^*2*^ = 0.06, *I* ^2^ = 37.6%) (Supplementary Figure S9).

Excluding one cohort study [61] that reported a hazard ratio and had the highest effect estimate, the pooled result remained statistically significant (RR = 1.71; 95% CI 1.40–2.10), with substantially reduced heterogeneity (*Q* = 1.05, *p* = 0.306; *τ* ^*2*^ = 0.00, *I* ^2^ = 4.4%) (Supplementary Figure S10).

### 3.8 NAFLD

**FIGURE 5:**
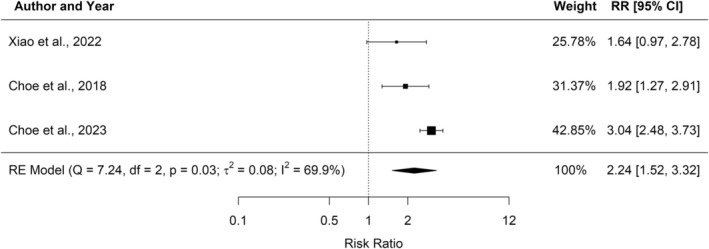
Forest plot illustrating the association between low FFM and risk of NAFLD.

The pooled effect estimate of 3 studies [40, 52, 56] indicated a statistically significant association between low FFM metrics and NAFLD (RR = 2.24; 95% CI 1.52–3.32) (Figure 5), with substantial heterogeneity across studies (*Q* = 7.24, *p* = 0.03; *τ* ^*2*^ = 0.08, *I* ^2^ = 69.9%).

Removing one study [56] that reported the highest effect estimate, the pooled estimate for the remaining studies remained statistically significant (RR = 1.81; 95% CI 1.31–2.50) and eliminated heterogeneity (*Q* = 0.21, *p* = 0.64; *τ* ^*2*^ = 0.00, *I* ^2^ = 0.0%) (Supplementary Figure S11). Conversely, excluding one type A study [52] (absolute FFM measure) yielded a statistically significant pooled estimate (RR = 2.51, 95% CI 1.61–3.91), although heterogeneity increased (*Q* = 3.80, *p* = 0.051; *τ* ^*2*^ = 0.08, *I* ^2^ = 73.7%) (Supplementary Figure S12).

### 3.9 Synthesis Without Meta‐Analysis

For outcomes with an insufficient number of studies, methodological incompatibilities, or incompatible exposure categories, we synthesized the evidence narratively, and the findings are presented below.

### 3.10 T2DM

Most studies reported inverse relationships between higher FFM metrics and the risk of T2DM or insulin resistance. Six studies [19, 26, 41, 50, 51, 58] reported significant inverse associations across one or more FFM metrics. However, some studies reported multiple FFM metrics within the same population, allowing direct comparison of how different FFM metrics influenced the observed associations. For example, Srikanthan et al. [51] found stronger inverse associations with T2DM for FFM metrics expressed relative to body weight than for height‐adjusted FFM metrics, whereas Li et al. [62] reported that although FFM percentage was inversely associated with incident T2DM in unadjusted analyses, neither FFM percentage nor height‐adjusted FFM metrics remained independently associated after multivariable adjustment. Across other studies, lower relative FFM was associated with an increased incidence of prediabetes and adverse glycemic measures, although no significant association was observed with overall incident T2DM and most associations with glucose and insulin measures were attenuated after adjustment for changes in adiposity [58]. Higher FFM percentage was associated with diabetes remission following laparoscopic sleeve gastrectomy [26], and each standard deviation decrease in FFM index was associated with a 35% increased risk of developing T2DM [19].

Conversely, three studies [28, 29, 49] reported direct associations between higher FFM measures and decreased insulin sensitivity. Two studies [28, 49] conducted among sedentary postmenopausal women found that higher appendicular lean body mass index was associated with lower insulin sensitivity, while one study [29] involving women with polycystic ovary syndrome reported that lean mass correlated positively with insulin resistance markers (HOMA) and inversely with insulin sensitivity markers (QUICKI). Two (17%) studies [27, 60] did not show statistically significant associations between FFM metrics and T2DM‐related outcomes.

### 3.11 CVD Risk

For CVD risk, five studies reported inverse associations between FFM metrics and cardiovascular outcomes [42, 46, 48, 54, 61]. Specifically, Tyrovolas et al. [61] reported that higher baseline SMI was independently associated with a lower 10‐year risk of CVD (HR = 0.06; 95% CI 0.005–0.78). Consistent with this finding, individuals in the highest SMI tertile had an 81% lower risk of developing CVD over 10 years than those in the lowest tertile (HR = 0.19, 95% CI 0.04–0.85). Kang et al. [42] reported that low skeletal muscle mass (defined using the ASM/BMI) was independently associated with increased carotid intima‐media thickness (OR = 2.26, 95% CI 1.26–4.04) and carotid plaque (OR = 2.74, 95% CI 1.30–5.78). Yoo et al. [54] found that individuals in the lowest relative FFM metric tertile had 56% higher odds of left ventricular diastolic dysfunction than those in the highest tertile (OR = 1.56, 95% CI 1.31–1.85).

Muster et al. [46] and Park et al. [48] also reported consistent findings using different FFM metrics, showing that lower lean mass and low muscle mass defined by ASM% were, respectively, independently associated with greater arterial stiffness, measured by augmentation index and cardio‐ankle vascular index. In contrast, two studies [45, 57]. reported adverse or null associations. Moreno et al. [45] found that higher lean mass was independently associated with increased carotid intima‐media thickness, while Fenger‐Grøn et al. [57] reported that higher lean body mass was the predominant risk factor for atrial fibrillation (HR = 1.37, 95% CI 1.33–1.42).

### 3.12 MetS

Two studies [39, 55] found a significant inverse association between adiposity‐adjusted FFM metrics and MetS. One study [39] reported that muscle mass adjusted for body weight was significantly associated with lower odds of MetS (OR = 0.06, 95% CI 0.02–0.21), while another study [55] showed that a 10% increase in muscle mass could prevent 14%–24% of MetS outcomes.

### 3.13 NAFLD outcomes

Two studies [40, 56] identified a clear significant association between FFM‐related metrics and non‐alcoholic fatty liver disease (NAFLD). Specifically, one study [40] reported that low FFM metrics were significantly associated with higher odds of NAFLD (OR = 1.51, 95% CI 1.15–1.99), while another study [56] showed that lower FFM increased the risk of NAFLD (HR = 1.26, 95% CI 1.04–1.53).

### 3.14 Cardiorespiratory Fitness

Two studies [38, 53] reported that greater FFM‐related measures were positively and significantly associated with cardiorespiratory fitness, as evidenced by higher VO_2_peak (*β* = 0.473, *p* < 0.001) and O_2_pulse_peak (*β* = 0.5312, *p* < 0.001).

### 3.15 Hypertension

One longitudinal study [31] found that annual increases in FFM were significantly associated with higher incidence of hypertension (β = 1.66, 95% CI 1.31–2.01), defined as systolic blood pressure ≥ 140 mmHg, diastolic blood pressure ≥ 90 mmHg, or the intake of antihypertensive medication at follow‐ups. Conversely, pooled data from the same study revealed that reduction in FFM metrics was associated with lower odds of hypertension remission (OR = 0.42, 95% CI 0.25–0.69).

### 3.16 Chronic Obstructive Pulmonary Disease

One study [44] reported that a low FFM index in individuals with obesity or overweight was directly associated with impaired lung function and reduced exercise tolerance.

### 3.17 Chronic Kidney Disease

One study [47] found that lean body mass indexed to serum creatinine (LBM/SCr) was an independent predictor of glomerular filtration rate (GFR) and improved the accuracy of GFR estimation in individuals with obesity or overweight.

## 4 Discussion

This review provides a synthesis of evidence on the associations between FFM‐related measures and multiple weight‐related complications in adults with overweight or obesity. Overall, the evidence suggested that lower FFM‐related metrics were generally associated with an increased risk of key metabolic complications, including T2DM, MetS, CVD, and NAFLD. However, these associations varied according to the type of FFM metric used, with differences observed between absolute, height‐adjusted, and relative or adiposity‐adjusted measures.

### 4.1 T2DM

Our meta‐analysis demonstrated a significant association between lower FFM‐related metrics and increased risk of T2DM. Despite substantial heterogeneity, leave‐one‐out sensitivity analyses and sex‐stratified analyses confirmed a stable association. These findings are consistent with previous meta‐analytic evidence reporting increased T2DM risk among individuals with lower FFM‐related metrics [63]. Several individual studies also reported inverse associations between reduced FFM‐related metrics and insulin resistance, prediabetes, and T2DM in adults with obesity [26, 41, 50].

A plausible biological explanation is the central role of skeletal muscle as the primary site of glucose uptake and metabolism [64]. Quantitatively, reductions in weight‐adjusted FFM were associated with substantially increased T2DM risk [19], while higher FFM metrics were associated with improved insulin sensitivity [51].

Higher FFM percentage was also associated with T2DM remission following bariatric surgery [26]. As absolute FFM typically decreases during weight loss, this finding suggests that relative preservation of lean mass may be a more relevant indicator of metabolic improvement in the context of bariatric surgery. However, whether this observation extends to other populations remains uncertain, and the sex‐specific findings were inconclusive.

Interventional studies have shown that resistance training and protein‐based interventions improve insulin sensitivity and glycemic control, supporting a role for muscle‐preserving strategies in diabetes prevention and management [65, 66, 67, 68, 69, 70]. However, observational studies have not been entirely consistent. Adverse or null associations have been reported among sedentary postmenopausal women, women with polycystic ovary syndrome, and older adults after adjustment for visceral adiposity, suggesting that the relationship between FFM and metabolic health may differ according to population characteristics, body composition, and the FFM metric used [29, 62].

### 4.2 MetS

Lower FFM‐related metrics, particularly weight‐related measures, were also significantly associated with increased prevalence of MetS. Despite moderate heterogeneity, the association remained robust across stratified analyses and was supported by individual studies reporting inverse relationships between FFM‐related metrics and MetS risk [55, 59]. One potential explanation is the metabolic role of skeletal muscle in glucose and lipid regulation. Reduced muscle mass may impair glucose disposal and promote insulin resistance and dyslipidemia, key features of MetS and T2DM [71, 72].

### 4.3 CVDs

Lower FFM‐related metrics were associated with more than a twofold increased risk of CVD, although moderate heterogeneity was observed. Associations were also evident across multiple cardiovascular outcomes, including atrial fibrillation, arterial stiffness, subclinical atherosclerosis, and left ventricular dysfunction [42, 46, 48, 54]. Higher FFM‐related metrics were associated with lower carotid intima‐media thickness, reduced carotid plaque burden, and lower long‐term CVD risk [42, 61]. Adverse associations were also observed in some populations [45, 57], suggesting that the relationship between FFM and cardiovascular health may vary according to the population characteristics and clinical context. Potential mechanisms include chronic low‐grade inflammation, insulin resistance, and dyslipidemia, which are associated with reduced skeletal muscle mass and increased adiposity [73, 74]. Exercise‐based interventions that improve body composition may also contribute to cardiovascular risk reduction.

### 4.4 NAFLD

Lower FFM‐related metrics were associated with increased risk of NAFLD. This is consistent with previous meta‐analytic evidence showing higher odds of NAFLD in individuals with low relative skeletal muscle mass [75]. Our broader synthesis extends this evidence to multiple FFM‐related metrics and related complications.

Mechanistically, reduced skeletal muscle mass may exacerbate insulin resistance and dysregulated lipid metabolism, contributing to hepatic fat accumulation and disease progression [56]. Supporting evidence also links lower FFM with progression of hepatic fibrosis [52, 56]. Interventions aimed at preserving or improving FFM‐related metrics, particularly skeletal muscle quantity and distribution, including resistance‐based exercise, may contribute to NAFLD prevention and management.

### 4.5 Heterogeneity and Subgroup Differences

Heterogeneity in associations was observed across studies and may partly reflect differences in how FFM was operationalized. Relative measures such as FFM/weight, FFM/BMI, and percentage FFM may be influenced by adiposity and changes in fat mass, which may contribute to differences in the observed associations across studies. Our subgroup analyses further demonstrated differences in the magnitude and consistency of associations according to FFM metric type.

Variability in findings across populations also suggests potential effect modification. In particular, one study reported that higher values of FFM‐related metrics were associated with adverse cardiometabolic risk markers in postmenopausal women with obesity, even after adjustment for central adiposity [49]. Together, these findings suggest that the relationship between FFM and metabolic risk may vary according to physiological characteristics, clinical context, and the FFM metric used.

These findings should be interpreted with caution because increases in FFM may occur alongside increases in overall body mass or adiposity. This is particularly relevant for relative FFM metrics, such as FFM percentage or weight‐relative measures, where changes in the denominator (body weight) may influence the observed associations even when absolute lean mass remains unchanged. These methodological differences may partly explain the heterogeneity observed across studies. Future research should prioritize standardized and clearly defined FFM‐related metrics to isolate the independent contribution of lean tissue mass to metabolic health.

### 4.6 Implications

These findings suggest that consideration of FFM‐related metrics during body composition assessment may provide additional insight into metabolic risk. However, whether targeting preservation of FFM directly improves metabolic outcomes independent of fat loss requires confirmation in interventional studies.

For researchers, these findings highlight the need for standardized reporting and clear definition of FFM metrics, together with consistent adjustment for adiposity where appropriate. For clinicians, assessment of body composition may improve risk stratification in obesity management. For policymakers, promoting muscle health across the lifespan may represent a potential preventive strategy, although specific targets and implementation strategies require further evidence.

### 4.7 Strengths and Limitations

This review has several strengths, including a comprehensive synthesis of evidence across multiple weight‐related complications, evaluation of different FFM‐related metrics, and extensive sensitivity and subgroup analyses that strengthened interpretation of the findings and identified potential sources of heterogeneity.

However, several limitations should be acknowledged, including reliance on observational studies, substantial heterogeneity in FFM definitions, and limited ability to distinguish skeletal muscle from total lean mass. Residual confounding by adiposity is also possible, as not all studies fully adjusted for fat mass. In addition, variability in study design and outcome definitions may have influenced the pooled estimates.

## 5 Conclusion

Lower FFM‐related metrics were generally associated with increased risk of T2DM, MetS, CVD, and NAFLD in adults with overweight or obesity, although the magnitude and consistency of these associations varied according to the FFM metrics used. These findings highlight the importance of considering FFM metric choice when interpreting associations between body composition and metabolic health. Future longitudinal and interventional studies using standardized FFM‐related metrics are needed to clarify causal relationships and support their appropriate clinical application.

## Author Contributions

Tadesse Asmamaw Dejenie and Evan Atlantis contributed to the conceptualization, investigation, data curation, and drafting of the original manuscript. Tadesse Asmamaw Dejenie, Evan Atlantis and Paul P. Fahey were responsible for the methodology, formal analysis, and visualization. Tadesse Asmamaw Dejenie, Evan Atlantis, Mohammad Ariya, and Haider Mannan contributed to the validation processes. Evan Atlantis, Paul P. Fahey, and Haider Mannan provided supervision. All authors reviewed and edited the manuscript and approved the final version of manuscript. All authors reviewed and edited the manuscript and approved the final version of manuscript.

## Funding

This study was supported by Western Sydney University Postgraduate Research Scholarship and the Commonwealth through an Australian Government Research Training Program Scholarship [DOI: https://doi.org/10.82133/C42F‐K220]. No additional funding was received from public, commercial, or not‐for‐profit agencies for this manuscript.

## Conflicts of Interest

T.A.D. is supported by the Western Sydney University Postgraduate Research Scholarship. E.A. holds an honorary position as Secretary of the National Association of Clinical Obesity Services (NACOS). The authors declare that there are no other financial and non‐financial competing interests.

## Supporting information




**Table S1:** Search strategy.
**Table S2:** List of excluded studies with reasons.
**Table S3:** Characteristics and summary of studies reviewed.
**Table S4:** Risk of bias assessment of studies reviewed.

## Data Availability

Not applicable.

## References

[1] P. L. Alebna , A. Mehta , A. Yehya , A. daSilva‐deAbreu , C. J. Lavie , and S. Carbone , “Update on Obesity, the Obesity Paradox, and Obesity Management in Heart Failure,” Progress in Cardiovascular Diseases 82 (2024): 34–42, 10.1016/j.pcad.2024.01.003. 38199320

[2] A. Magomedova , “The Analysis of the Main Contributing Factors for the Increasing Trend of Diabetes Type 2, Diabetes Morbidity and Mortality Trends in England, 2017‐2022,” Medical Research Archives 11, no. 9 (2023), 10.18103/mra.v11i9.4403.

[3] A. M. Jastreboff , C. M. Kotz , S. Kahan , A. S. Kelly , and S. B. Heymsfield , “Obesity as a Disease: The Obesity Society 2018 Position Statement,” Obesity (Silver Spring) 27, no. 1 (2019): 7–9, 10.1002/oby.22378. 30569641

[4] M. Wiseman , “The Second World Cancer Research Fund/American Institute for Cancer Research Expert Report. Food, Nutrition, Physical Activity, and the Prevention of Cancer: A Global Perspective,” Proceedings of the Nutrition Society 67, no. 3 (2008): 253–256, 10.1017/s002966510800712x. 18452640

[5] A. Dee , K. Kearns , C. O'Neill , et al., “The Direct and Indirect Costs of Both Overweight and Obesity: A Systematic Review,” BMC Research Notes 7 (2014): 242, 10.1186/1756-0500-7-242. 24739239 PMC4006977

[6] M. L. Specchia , M. A. Veneziano , C. Cadeddu , et al., “Economic Impact of Adult Obesity on Health Systems: A Systematic Review,” European Journal of Public Health. 25, no. 2 (2015): 255–262. 25320051 10.1093/eurpub/cku170

[7] S. Colagiuri , C. M. Y. Lee , R. Colagiuri , et al., “The Cost of Overweight and Obesity in Australia,” Medical journal of Australia. 192, no. 5 (2010): 260–264, 10.5694/j.1326-5377.2010.tb03503.x. 20201759

[8] M. A. Nagi , H. Ahmed , M. A. A. Rezq , et al., “Economic Costs of Obesity: A Systematic Review,” International Journal of Obesity. 48, no. 1 (2024): 33–43, 10.1038/s41366-023-01398-y. 37884664

[9] E. Atlantis , M. Sahebolamri , B. S. Cheema , and K. Williams , “Usefulness of the Edmonton Obesity Staging System for Stratifying the Presence and Severity of Weight‐Related Health Problems in Clinical and Community Settings: A Rapid Review of Observational Studies,” Obesity Reviews 21, no. 11 (2020): e13120, 10.1111/obr.13120. 32812345

[10] E. Atlantis , K. Lange , and G. A. Wittert , “Chronic Disease Trends Due to Excess Body Weight in Australia,” Obesity Reviews 10, no. 5 (2009): 543–553, 10.1111/j.1467-789X.2009.00590.x. 19413699

[11] M. Kivimäki , T. Strandberg , J. Pentti , et al., “Body‐Mass Index and Risk of Obesity‐Related Complex Multimorbidity: An Observational Multicohort Study,” Lancet Diabetes and Endocrinology 10, no. 4 (2022): 253–263, 10.1016/s2213-8587(22)00033-x. 35248171 PMC8938400

[12] X. D. Zhou , Q. F. Chen , W. Yang , et al., “Burden of Disease Attributable to High Body Mass Index: An Analysis of Data From the Global Burden of Disease Study 2021,” EClinicalMedicine 76 (2024): 102848, 10.1016/j.eclinm.2024.102848. 39386160 PMC11462227

[13] C. M. Prado , S. M. Phillips , M. C. Gonzalez , and S. B. Heymsfield , “Muscle Matters: The Effects of Medically Induced Weight Loss on Skeletal Muscle,” Lancet Diabetes and Endocrinology 12, no. 11 (2024): 785–787, 10.1016/s2213-8587(24)00272-9. 39265590

[14] R. Kawakami , K. Tanisawa , T. Ito , et al., “Fat‐Free Mass Index as a Surrogate Marker of Appendicular Skeletal Muscle Mass Index for Low Muscle Mass Screening in Sarcopenia,” Journal of the American Medical Directors Association 23, no. 12 (2022): 1955–1961.e3, 10.1016/j.jamda.2022.08.016. 36179769

[15] K. M. Kim , H. C. Jang , and S. Lim , “Differences Among Skeletal Muscle Mass Indices Derived From Height‐, Weight‐, and Body Mass Index‐Adjusted Models in Assessing Sarcopenia,” Korean Journal of Internal Medicine 31, no. 4 (2016): 643–650, 10.3904/kjim.2016.015. 27334763 PMC4939509

[16] C. O. Walowski , W. Braun , M. J. Maisch , et al., “Reference Values for Skeletal Muscle Mass ‐ Current Concepts and Methodological Considerations,” Nutrients 12, no. 3 (2020): 755, 10.3390/nu12030755. 32178373 PMC7146130

[17] A. R. Cho , J. H. Lee , and Y. J. Kwon , “Differences Among Three Skeletal Muscle Mass Indices in Predicting Non‐Alcoholic Fatty Liver Disease: Korean Nationwide Population‐Based Study,” Life (Basel) 11, no. 8 (2021): 751, 10.3390/life11080751. 34440495 PMC8401633

[18] J. A. Batsis , T. A. Mackenzie , R. T. Emeny , F. Lopez‐Jimenez , and S. J. Bartels , “Low Lean Mass With and Without Obesity, and Mortality: Results From the 1999‐2004 National Health and Nutrition Examination Survey,” journals of gerontology Series A, Biological sciences and medical sciences. 72, no. 10 (2017): 1445–1451, 10.1093/gerona/glx002. 28207042 PMC5861857

[19] J. W. Son , S. S. Lee , S. R. Kim , et al., “Low Muscle Mass and Risk of Type 2 Diabetes in Middle‐Aged and Older Adults: Findings From the KoGES,” Diabetologia 60, no. 5 (2017): 865–872. 28102434 10.1007/s00125-016-4196-9

[20] S. Hong , Y. Chang , H.‐S. Jung , K. E. Yun , H. Shin , and S. Ryu , “Relative Muscle Mass and the Risk of Incident Type 2 Diabetes: A Cohort Study,” PLoS ONE 12, no. 11 (2017): e0188650, 10.1371/journal.pone.0188650. 29190709 PMC5708784

[21] A. Tinggaard , M. Skou , N. Jessen , and H. Wiggers , “How to Identify Patients With Low Muscle Mass Linked to Physical Capacity and Physical Activity in Heart Failure,” European Heart Journal 43 (2022): ehac544. 1045.

[22] M. N. LeCroy , S. Hua , R. C. Kaplan , et al., “Associations of Changes in Fat Free Mass with Risk for Type 2 Diabetes: Hispanic Community Health Study/Study of Latinos,” Diabetes Research and Clinical Practice. 171 (2021): 108557, 10.1016/j.diabres.2020.108557. 33242517 PMC8425264

[23] A. G. Dulloo , J. Jacquet , J. L. Miles‐Chan , and Y. Schutz , “Passive and Active Roles of Fat‐Free Mass in the Control of Energy Intake and Body Composition Regulation,” European journal of clinical nutrition. 71, no. 3 (2017): 353–357, 10.1038/ejcn.2016.256. 27966570

[24] S. Zaki , M. F. Alam , S. Sharma , S. El‐Ashker , M. Ahsan , and S. Nuhmani , “Impact of Concurrent Exercise Training on Cardiac Autonomic Modulation, Metabolic Profile, Body Composition, Cardiorespiratory Fitness, and Quality of Life in Type 2 Diabetes with Cardiac Autonomic Neuropathy: A Randomized Controlled Trial,” Journal of Clinical Medicine 13, no. 13 (2024): 3910. 38999476 10.3390/jcm13133910 PMC11242881

[25] K. Hejazi , G. R. Mohammad Rahimi , and S. K. Rosenkranz , “Effects of Exercise Training on Inflammatory and Cardiometabolic Risk Biomarkers in Patients With Type 2 Diabetes Mellitus: A Systematic Review and Meta‐Analysis of Randomized Controlled Trials,” Biological Research For Nursing. 25, no. 2 (2023): 250–266. 36213963 10.1177/10998004221132841

[26] N. T. K. Nguyen , N.‐P. Vo , S.‐Y. Huang , and W. Wang , “Fat‐Free Mass and Skeletal Muscle Mass Gain Are Associated With Diabetes Remission After Laparoscopic Sleeve Gastrectomy in Males but Not in Females,” International Journal of Environmental Research and Public Health 19, no. 2 (2022): 978, 10.3390/ijerph19020978. 35055799 PMC8776008

[27] P. Pramyothin , V. Limpattanachart , S. Dawilai , et al., “Fat‐Free Mass, Metabolically Healthy Obesity, and Type 2 Diabetes in Severely Obese Asian Adults,” Endocrine Practice 23, no. 8 (2017): 915–922, 10.4158/EP171792.OR. 28614006

[28] R. Barsalani , M. Brochu , and I. J. Dionne , “Is There a Skeletal Muscle Mass Threshold Associated With the Deterioration of Insulin Sensitivity in Sedentary Lean to Obese Postmenopausal Women?” Diabetes Research and Clinical Practice 102, no. 2 (2013): 123–128. 24120357 10.1016/j.diabres.2013.09.008

[29] K. B. Comerford , R. U. Almario , K. Kim , and S. E. Karakas , “Lean Mass and Insulin Resistance in Women with Polycystic Ovary Syndrome,” Metabolism: Clinical and Experimental 61, no. 9 (2012): 1256–1260. 22424820 10.1016/j.metabol.2012.02.004

[30] B. A. Larsen , C. L. Wassel , S. B. Kritchevsky , et al., “Association of Muscle Mass, Area, and Strength With Incident Diabetes in Older Adults: The Health ABC Study,” Journal of Clinical Endocrinology & Metabolism. 101, no. 4 (2016): 1847–1855, 10.1210/jc.2015-3643. 26930180 PMC4880161

[31] T. Ittermann , N. Werner , W. Lieb , et al., “Changes in Fat Mass and Fat‐Free‐Mass Are Associated With Incident Hypertension in Four Population‐Based Studies From Germany,” International Journal of Cardiology 274 (2019): 372–377. 30217425 10.1016/j.ijcard.2018.09.035

[32] E. Aromataris , C. Lockwood , K. Porritt , B. Pilla , and Z. Jordan , JBI Manual for Evidence Synthesis (JBI, 2024).

[33] M. J. Page , J. E. McKenzie , P. M. Bossuyt , et al., “The PRISMA 2020 Statement: An Updated Guideline for Reporting Systematic Reviews,” BMJ 372 (2020): n71, 10.1136/bmj.n71. PMC8005924 33782057

[34] B. S. Brooke , T. A. Schwartz , and T. M. Pawlik , “MOOSE Reporting Guidelines for Meta‐Analyses of Observational Studies,” JAMA Surgery 156, no. 8 (2021): 787–788, 10.1001/jamasurg.2021.0522. 33825847

[35] C. Schardt , M. B. Adams , T. Owens , S. Keitz , and P. Fontelo , “Utilization of the PICO Framework to Improve Searching PubMed for Clinical Questions,” BMC Medical Informatics and Decision Making 7 (2007): 16, 10.1186/1472-6947-7-16. 17573961 PMC1904193

[36] M. D. Peters , C. M. Godfrey , P. McInerney , C. B. Soares , H. Khalil , and D. Parker , “The Joanna Briggs Institute Reviewers' Manual 2015: Methodology for JBI Scoping Reviews,” (2015).

[37] J. Chandler , M. Cumpston , T. Li , M. J. Page , and V. Welch , Cochrane Handbook for Systematic Reviews of Interventions, vol. 4, 1002 (Wiley, 2019): 14651858.

[38] S. H. Boo , M. C. Joo , J. M. Lee , S. C. Kim , Y. M. Yu , and M. S. Kim , “Association Between Skeletal Muscle Mass and Cardiorespiratory Fitness in Community‐Dwelling Elderly Men,” Aging Clinical and Experimental Research 31, no. 1 (2019): 49–57. 29916089 10.1007/s40520-018-0987-9

[39] C. J. Carvalho , G. Z. Longo , A. M. Kakehasi , et al., “Association Between Skeletal Mass Indices and Metabolic Syndrome in Brazilian Adults,” Journal of Clinical Densitometry 24, no. 1 (2021): 118–128. 32205007 10.1016/j.jocd.2020.02.003

[40] E. K. Choe , H. Y. Kang , B. Park , J. I. Yang , and J. S. Kim , “The Association Between Nonalcoholic Fatty Liver Disease and CT‐Measured Skeletal Muscle Mass,” Journal of Clinical Medicine 7, no. 10 (2018): 310, 10.3390/jcm7100310. 30274215 PMC6211085

[41] R. Fornari , D. Francomano , E. A. Greco , et al., “Lean Mass in Obese Adult Subjects Correlates With Higher Levels of Vitamin D, Insulin Sensitivity and Lower Inflammation,” Journal of Endocrinological Investigation 38, no. 3 (2015): 367–372, 10.1007/s40618-014-0189-z. 25352234

[42] M. K. Kang and J. G. Park , “Low Skeletal Muscle Mass is a Risk Factor for Subclinical Atherosclerosis in Patients with Nonalcoholic Fatty Liver Disease,” Diagnostics 11, no. 5 (2021): 854, 10.3390/diagnostics11050854. 34068776 PMC8150334

[43] H. S. Koo , M. J. Kim , K. M. Kim , and Y. S. Kim , “Decreased Muscle Mass Is not an Independent Risk Factor for Metabolic Syndrome in Korean Population Aged 70 or Older,” Clinical Endocrinology. 82, no. 4 (2015): 509–516. 24965115 10.1111/cen.12509

[44] F. V. C. Machado , M. A. Spruit , M. T. J. Groenen , et al., “Frequency and Functional Translation of Low Muscle Mass in Overweight and Obese Patients With COPD,” Respiratory Research. 22, no. 1 (2021): 93. 33766023 10.1186/s12931-021-01689-w PMC7993483

[45] M. Moreno , J. Puig , J. M. Moreno‐Navarrete , et al., “Lean Mass, and not Fat Mass, Is an Independent Determinant of Carotid Intima Media Thickness in Obese Subjects,” Atherosclerosis 243, no. 2 (2015): 493–498, 10.1016/j.atherosclerosis.2015.09.012. 26520905

[46] V. Muster , K. Gutl , G. Pregartner , et al., “Lower Lean Mass is Associated With Greater Arterial Stiffness in Patients With Lower Extremity Artery Disease,” Journal of Personalized Medicine 11, no. 9 (2021): 911, 10.3390/jpm11090911. 34575687 PMC8470700

[47] S. Ozmen , M. A. Kaplan , H. Kaya , et al., “Role of Lean Body Mass for Estimation of Glomerular Filtration Rate in Patients With Chronic Kidney Disease With Various Body Mass Indices,” SCANDINAVIAN JOURNAL OF UROLOGY AND NEPHROLOGY. 43, no. 2 (2009): 171–176, 10.1080/00365590802502228. 18979280

[48] H. E. Park , G. E. Chung , H. Lee , et al., “Significance of Low Muscle Mass on Arterial Stiffness as Measured by Cardio‐Ankle Vascular Index,” Frontiers in Cardiovascular Medicine 9 (2022): 857871, 10.3389/fcvm.2022.857871. 35774369 PMC9239409

[49] M. Peppa , C. Koliaki , E. Boutati , et al., “Association of Lean Body Mass With Cardiometabolic Risk Factors in Healthy Postmenopausal Women,” Obesity 22, no. 3 (2014): 828–835. 23512933 10.1002/oby.20389

[50] Y. Shao , L. Li , H. Zhong , X. Wang , Y. Hua , and X. Zhou , “Anticipated Correlation Between Lean Body Mass to Visceral Fat Mass Ratio and Insulin Resistance: NHANES 2011‐2018,” Frontiers in Endocrinology 14 (2023): 1232896, https://10.3389/fendo.2023.1232896. 37772076 10.3389/fendo.2023.1232896 PMC10526824

[51] P. Srikanthan and A. S. Karlamangla , “Relative Muscle Mass Is Inversely Associated With Insulin Resistance and Prediabetes. Findings From the third National Health and Nutrition Examination Survey,” Journal of Clinical Endocrinology & Metabolism. 96, no. 9 (2011): 2898–2903. 21778224 10.1210/jc.2011-0435

[52] P. Xiao , P. Liang , P. Gao , and J. Wu , “Sex‐ and Region‐Specific Associations of Skeletal Muscle Mass with Metabolic Dysfunction‐Associated Fatty Liver Disease,” Frontiers in Endocrinology 13 (2022): 1057261, 10.3389/fendo.2022.1057261. 36531457 PMC9755203

[53] L. R. Yanek , D. Vaidya , B. G. Kral , et al., “Lean Mass and Fat Mass as Contributors to Physical Fitness in an Overweight and Obese African American Population,” Ethnicity & Disease. 25, no. 2 (2015): 214–219. 26118151 PMC4790079

[54] J. H. Yoo , S. W. Park , J. E. Jun , et al., “Relationship Between Low Skeletal Muscle Mass, Sarcopenic Obesity and Left Ventricular Diastolic Dysfunction in Korean Adults,” Diabetes/Metabolism Research and Reviews 37, no. 1 (2021): e3363. 32521113 10.1002/dmrr.3363

[55] E. Atlantis , S. A. Martin , M. T. Haren , A. W. Taylor , and G. A. Wittert , “Inverse Associations Between Muscle Mass, Strength, and the Metabolic Syndrome,” Metabolism 58, no. 7 (2009): 1013–1022, 10.1016/j.metabol.2009.02.027. 19394973

[56] H. J. Choe , H. Lee , D. Lee , S. H. Kwak , and B. K. Koo , “Different Effects of Low Muscle Mass on the Risk of Non‐Alcoholic Fatty Liver Disease and Hepatic Fibrosis in a Prospective Cohort,” Journal of Cachexia, Sarcopenia and Muscle. 14, no. 1 (2023): 260–269. 36403577 10.1002/jcsm.13125 PMC9891951

[57] M. Fenger‐Gron , K. Overvad , A. Tjonneland , and L. Frost , “Lean Body Mass Is the Predominant Anthropometric Risk Factor for Atrial Fibrillation,” Journal of the American College of Cardiology. 69, no. 20 (2017): 2488–2497. 28521886 10.1016/j.jacc.2017.03.558

[58] M. N. LeCroy , S. Hua , R. C. Kaplan , et al., “Associations of Changes in Fat Free Mass with Risk for Type 2 Diabetes: Hispanic Community Health Study/Study of Latinos,” Diabetes Research and Clinical Practice 171 (2021): 108557. 33242517 10.1016/j.diabres.2020.108557 PMC8425264

[59] M. J. Lee , E. H. Kim , S. J. Bae , et al., “Protective Role of Skeletal Muscle Mass Against Progression From Metabolically Healthy to Unhealthy Phenotype,” Clinical Endocrinology. 90, no. 1 (2019): 102–113. 30290006 10.1111/cen.13874

[60] D. Sizoo , S. P. Stam , L. J. M. de Heide , et al., “The Association of Low Muscle Mass With Prevalence and Incidence of Type 2 Diabetes in Different BMI Classes,” Diabetes Research and Clinical Practice 195 (2023): 110197. 36464089 10.1016/j.diabres.2022.110197

[61] S. Tyrovolas , D. Panagiotakos , E. Georgousopoulou , et al., “Skeletal Muscle Mass in Relation to 10 Year Cardiovascular Disease Incidence Among Middle Aged and Older Adults: The ATTICA Study,” Journal of Epidemiology & Community Health. 74, no. 1 (2020): 26–31. 31712252 10.1136/jech-2019-212268 PMC6929696

[62] J. J. Li , G. A. Wittert , A. Vincent , et al., “Muscle Grip Strength Predicts Incident Type 2 Diabetes: Population‐Based Cohort Study,” Metabolism 65, no. 6 (2016): 883–892, 10.1016/j.metabol.2016.03.011. 27173467

[63] P. Gupta , C. Lanca , A. T. L. Gan , et al., “The Association between Body Composition Using Dual Energy X‐Ray Absorptiometry and Type‐2 Diabetes: A Systematic Review and Meta‐Analysis of Observational Studies,” Scientific Reports 9, no. 1 (2019): 12634, 10.1038/s41598-019-49162-5. 31477766 PMC6718404

[64] S. Lillioja , C. Bogardus , D. M. Mott , A. L. Kennedy , W. C. Knowler , and B. V. Howard , “Relationship Between Insulin‐Mediated Glucose Disposal and Lipid Metabolism in Man,” Journal of Clinical Investigation 75, no. 4 (1985): 1106–1115, 10.1172/jci111804. 3886702 PMC425433

[65] C. Castaneda , J. E. Layne , L. Munoz‐Orians , et al., “A Randomized Controlled Trial of Resistance Exercise Training to Improve Glycemic Control in Older Adults With Type 2 Diabetes,” Diabetes Care 25, no. 12 (2002): 2335–2341, 10.2337/diacare.25.12.2335. 12453982

[66] D. W. Dunstan , R. M. Daly , N. Owen , et al., “High‐Intensity Resistance Training Improves Glycemic Control in Older Patients With Type 2 Diabetes,” Diabetes Care 25, no. 10 (2002): 1729–1736, 10.2337/diacare.25.10.1729. 12351469

[67] W. J. Pasman , R. G. Memelink , J. de Vogel‐Van den Bosch , et al., “Obese Older Type 2 Diabetes Mellitus Patients with Muscle Insulin Resistance Benefit from an Enriched Protein Drink during Combined Lifestyle Intervention: The PROBE Study,” Nutrients 12, no. 10 (2020): 2979, 10.3390/nu12102979. 33003389 PMC7601009

[68] M. Al‐Badri , A. Almasih Barbar Askar , A. Khater , et al., “14‐PUB: The Effect of Structured Intensive Lifestyle Intervention on Muscle Mass in Patients with Type 2 Diabetes Receiving GLP‐1 Receptor Agonists,” Diabetes 73 (2024): 14‐PUB.

[69] G. Wittert , K. Bracken , K. P. Robledo , et al., “Testosterone Treatment to Prevent or Revert Type 2 Diabetes in Men Enrolled in a Lifestyle Programme (T4DM): A Randomised, Double‐Blind, Placebo‐Controlled, 2‐Year, Phase 3b Trial,” Lancet Diabetes and Endocrinology 9, no. 1 (2021): 32–45, 10.1016/s2213-8587(20)30367-3. 33338415

[70] A. K. Jansson , L. X. Chan , D. R. Lubans , M. J. Duncan , and R. C. Plotnikoff , “Effect of Resistance Training on HbA1c in Adults with Type 2 Diabetes Mellitus and the Moderating Effect of Changes in Muscular Strength: A Systematic Review and Meta‐Analysis,” BMJ Open Diabetes Research & Care 10, no. 2 (2022): e002595, 10.1136/bmjdrc-2021-002595. PMC8915309 35273011

[71] M. P. Castillo Í , J. M. Argilés , R. Rueda , M. Ramírez , and J. M. L. Pedrosa , “Skeletal Muscle Atrophy and Dysfunction in Obesity and Type‐2 Diabetes Mellitus: Myocellular Mechanisms Involved,” Reviews in Endocrine & Metabolic Disorders 26 (2025): 815–836, 10.1007/s11154-025-09954-9. 40064750 PMC12534344

[72] C. Han , H. Xue , S. Yang , and B. Gao , “Resistance Exercise Training and Its Impact on Metabolic Syndrome in Type 2 Diabetes: A Systematic Review and Meta‐Analysis of Randomized Controlled Trials,” Diabetes Research and Clinical Practice 222 (2025): 112077, 10.1016/j.diabres.2025.112077. 40064302

[73] W. Dai , Z. Liu , S. Yang , and J. Kong , “Inflamed Adipose Tissue: Therapeutic Targets for Obesity‐Related Endothelial Injury,” Endocrinology 164, no. 7 (2023): bqad094, 10.1210/endocr/bqad094. 37289080

[74] V. T. Samuel and G. I. Shulman , “Mechanisms for Insulin Resistance: Common Threads and Missing Links,” Cell. 148, no. 5 (2012): 852–871, 10.1016/j.cell.2012.02.017. 22385956 PMC3294420

[75] C. Cai , X. Song , Y. Chen , X. Chen , and C. Yu , “Relationship Between Relative Skeletal Muscle Mass and Nonalcoholic Fatty Liver Disease: A Systematic Review and Meta‐Analysis,” Hepatology international. 14, no. 1 (2020): 115–126. 31290072 10.1007/s12072-019-09964-1 PMC6994447

