# Supplementary material for: The Role of Fat‐Free Mass in Weight‐Related Complications: A Rapid Review and Meta‐Analysis
Source: Obes Rev. 2026 Jul 21;27(9):e70202. doi: 10.1111/obr.70202 (PMC13460688; doi:10.1111/obr.70202)
Supplement: Supplementary file 1 [file OBR-27-e70202-s001.pdf]

The role of fat-free mass in weight-related complications: A rapid review and meta-analysis

Tadesse Asmamaw Dejenie[1, 2], Paul P Fahey[1, 3], Haider Mannan[3], Mohammad Ariya[1], Evan Atlantis[1, 3]

[1] School of Health Sciences, Western Sydney University, Campbelltown, New South Wales, Australia

[2]Department of Biochemistry, College of Medicine and Health Sciences, University of Gondar, Gondar, Ethiopia

[3]Translational Health Research Institute, Western Sydney University, Campbelltown, New South Wales, Australia

Appendix I: Supplementary Materials

Table of Contents Page

Supplementary Table S1: Search strategy 2
Ovid MEDLINE(R) ALL 2
Embase via OvidSP 4
Web of Science 5
Google Scholar 8
Supplementary Table S2: List of excluded studies with reasons 8
Supplementary Table S3: Characteristics and summary of studies reviewed. 9
Supplementary Table S4: Risk of bias assessment of studies reviewed 45
Forest plots of sensitivity and subgroup analyses for the association between low FFM metrics and T2DM 50
References for excluded studies 56

Supplementary Table S1: Search strategy

Ovid MEDLINE(R) ALL

#	Query	Results
1	(Adult* or "Aged 18 and Older" or "middle aged").ti,ab.	1,759,091
2	Adult/ or Middle Aged/	7,379,508
3	1 or 2	8,309,432
4	(Obes* or Overweight or adipos* or overnutrition or "nutrition disorder").ti,ab.	528,178
5	Obesity/ or Nutrition Disorders/ or Overnutrition/ or Overweight/ or Adipose Tissue/ or Body Weight/	509,075
6	4 or 5	779,943
7	3 and 6	282,090
8	("Fat-free mass" or FFM or "lean body" or "lean mass" or "lean tissue" or "muscle mass" or "skeleton muscle" or "muscle mass index" or "appendicular mass" or "body composition").ti,ab.	88,034
9	Body Composition/ or Muscle, Skeletal/ or skeletal muscles/ or muscle tissue/	366,493
10	8 or 9	411,501
11	("Chronic diseases" or "Chronic illness" or "Chronic conditions" or "Type 2 diabetes" or "cardiovascular disease*" or CVD or "coronary heart disease" or "Coronary Disease" or "ischaemic heart diseases" or "Heart failure" or "angina pectoris" or "Coronary artery Disease" or "Myocardial infarction" or "thromboembolic diseases" or Preeclampsia or stroke or "cerebrovascular diseases" or hypertens* or metabolic syndrome or cancer or "High Blood Pressure" or "Fatty Liver" or NAFLD or "Hepatic dysfunction" or Dyslipid?emia or "Insulin Resistance" or "Chronic obstructive pulmonary disease" or "impaired glucose tolerance" or CKD or "Kidney disease*" or "renal insufficiency" or osteoarthritis or Weight-related problem* or "Weight-related complication*" or "obesity-related" or "Mental disorder*" or Depression or anxiety or "Obstructive sleep apnea" or OSA or "Polycystic ovary syndrome" or "Gastro-oesophageal reflux disease"	6,153,638

	or GORD or "binge eating disorder" or "disordered eating behaviour" or "Stress-related disorder*" or stress or "Functional limitation*" or "mobility restriction*" or Gout or "physical limitation*" or disabilit* or "impaired wellbeing" or "six-minute walk test" or 6MWT or Vo2max or "Oxygen Consumption" or fitness or "activities of daily living").ti,ab.	
12	Diabetes Mellitus, Type 2/ or Chronic Disease/ or cardiovascular diseases/ or Coronary Disease/ or Myocardial Ischemia/ or Heart Failure/ or Angina Pectoris/ or coronary artery disease/ or Myocardial Infarction/ or Pre-Eclampsia/ or Cerebrovascular Disorders/ or Hypertension/ or Metabolic Syndrome/ or Non-alcoholic Fatty Liver Disease/ or Dyslipidemias/ or Insulin Resistance/ or Pulmonary Disease, Chronic Obstructive/ or Kidney Failure, Chronic/ or Kidney Diseases/ or Renal Insufficiency/ or Gout/ or Osteoarthritis/ or Mental Disorders/ or Depression/ or Anxiety/ or Sleep Apnea, Obstructive/ or Polycystic Ovary Syndrome/ or Gastroesophageal Reflux/ or Binge-Eating Disorder/ or Walk Test/ or Oxygen Consumption/ or Physical Fitness/ or "Activities of Daily Living"/	2,477,569
13	11 or 12	7,206,920
14	(((cohort or cross sectional or observation) adj stud*) or mendelian randomization).ti,ab.	709,576
15	cohort studies/ or follow-up studies/ or longitudinal studies/ or prospective studies/ or retrospective studies/ or cross-sectional studies/ or Mendelian Randomization Analysis/ or observational study/	3,197,486
16	14 or 15	3,397,187
17	7 and 10 and 13 and 16	4,107
18	limit 17 to english language	3885

Embase via OvidSP

#	Query	Results
1	(Adult* or "Aged 18 and Older" or "middle aged").ti,ab.	2,358,549
2	Adult/ or Middle Aged/	10,916,331
3	1 or 2	11,784,437
4	(Obes* or Overweight or adipos* or overnutrition or "nutrition disorder").ti,ab.	782,204
5	Obesity/ or Nutrition Disorders/ or Overnutrition/ or Overweight/ or Adipose Tissue/ or Body Weight/	1,087,393
6	4 or 5	1,320,678
7	3 and 6	545,319
8	("Fat-free mass" or FFM or "lean body" or "lean mass" or "lean tissue" or "muscle mass" or "skeleton muscle" or "muscle mass index" or "appendicular mass" or "body composition").ti,ab.	123,297
9	Body Composition/ or Muscle, Skeletal/ or skeletal muscles/ or muscle tissue/	257,247
10	8 or 9	306,125
11	("Chronic diseases" or "Chronic illness" or "Chronic conditions" or "Type 2 diabetes" or "cardiovascular disease*" or CVD or "coronary heart disease" or "Coronary Disease" or "ischaemic heart diseases" or "Heart failure" or "angina pectoris" or "Coronary artery Disease" or "Myocardial infarction" or "thromboembolic diseases" or Preeclampsia or stroke or "cerebrovascular diseases" or hypertens* or metabolic syndrome or cancer or "High Blood Pressure" or "Fatty Liver" or NAFLD or "Hepatic dysfunction" or Dyslipid?emia or "Insulin Resistance" or "Chronic obstructive pulmonary disease" or "impaired glucose tolerance" or CKD or "Kidney disease*" or "renal insufficiency" or osteoarthritis or Weight-related problem* or "Weight-related complication*" or "obesity-related" or "Mental disorder*" or Depression or anxiety or "Obstructive sleep apnea" or OSA or "Polycystic ovary syndrome" or "Gastro-oesophageal reflux disease" or GORD	8,787,545

	or "binge eating disorder" or "disordered eating behaviour" or "Stress-related disorder*" or stress or "Functional limitation*" or "mobility restriction*" or Gout or "physical limitation*" or disabilit* or "impaired wellbeing" or "six-minute walk test" or 6MWT or Vo2max or "Oxygen Consumption" or fitness or "activities of daily living").ti,ab.	
12	Diabetes Mellitus, Type 2/ or Chronic Disease/ or cardiovascular diseases/ or Coronary Disease/ or Myocardial Ischemia/ or Heart Failure/ or Angina Pectoris/ or coronary artery disease/ or Myocardial Infarction/ or Pre-Eclampsia/ or Cerebrovascular Disorders/ or Hypertension/ or Metabolic Syndrome/ or Non-alcoholic Fatty Liver Disease/ or Dyslipidemias/ or Insulin Resistance/ or Pulmonary Disease, Chronic Obstructive/ or Kidney Failure, Chronic/ or Kidney Diseases/ or Renal Insufficiency/ or Gout/ or Osteoarthritis/ or Mental Disorders/ or Depression/ or Anxiety/ or Sleep Apnea, Obstructive/ or Polycystic Ovary Syndrome/ or Gastroesophageal Reflux/ or Binge-Eating Disorder/ or Walk Test/ or Oxygen Consumption/ or Physical Fitness/ or "Activities of Daily Living"/	4,690,865
13	11 or 12	10,550,991
14	(((cohort or cross sectional or observation) adj stud*) or mendelian randomization).ti,ab.	966,225
15	cohort studies/ or follow-up studies/ or longitudinal studies/ or prospective studies/ or retrospective studies/ or cross-sectional studies/ or Mendelian Randomization Analysis/ or observational study/	5,673,859
16	14 or 15	5,769,161
17	7 and 10 and 13 and 16	8,661
18	limit 17 to (english language and "remove medline records")	1130

Web of Science

#	Query	Results

1	(TI= (Adult* OR "Aged 18 and Older" OR "adult population" OR "adult patients" OR adulthood OR "Older Adult*")) OR AB= (Adult* OR "Aged 18 and Older" OR "adult population" OR "adult patients" OR adulthood OR "Older Adult*")	2,027,058
2	(TI= (Obes* OR Overweight OR adipos* OR overnutrition OR "nutrition disorder")) OR AB= (Obes* OR Overweight OR adipos* OR overnutrition OR "nutrition disorder")	596,172
3	(TI=("Fat-free mass" OR FFM OR "lean body" OR "lean mass" OR "lean tissue" OR "muscle mass" OR "skeleton muscle" OR "muscle mass index" OR "appendicular mass" OR "body composition")) OR AB=("Fat-free mass" OR FFM OR "lean body" OR "lean mass" OR "lean tissue" OR "muscle mass" OR "skeleton muscle" OR "muscle mass index" OR "appendicular mass" OR "body composition")	103,239
4	(TI=("Chronic diseases" OR "Chronic illness" OR "Chronic conditions" OR "Type 2 diabetes" OR “Diabetes Mellitus, Type 2” OR "cardiovascular disease*" OR CVD OR "coronary heart disease" OR “Coronary Disease” OR "ischaemic heart diseases" OR "Heart failure" OR "angina pectoris" OR "Coronary artery Disease" OR "Myocardial infarction" OR "thromboembolic diseases" OR Preeclampsia OR stroke OR "cerebrovascular diseases" OR hypertens* OR metabolic syndrome OR cancer OR "High Blood Pressure" OR "Fatty Liver" OR NAFLD OR "Hepatic dysfunction" OR Dyslipid?emia OR "Insulin Resistance" OR "Chronic obstructive pulmonary disease" OR "impaired glucose tolerance" OR CKD OR "Kidney disease*" OR "renal insufficiency" OR Gout OR osteoarthritis OR Weight-related problem* OR "Weight-related complication*" OR "obesity-related” OR "Mental disorder*" OR Depression OR anxiety OR "Obstructive sleep apnea" OR OSA OR "Polycystic ovary syndrome" OR "Gastro-oesophageal reflux disease" OR GORD OR "binge eating disorder" OR "disordered eating behaviour" OR "Stress-related disorder*" OR stress OR "Functional limitation*" OR "mobility restriction*" OR Gout OR "physical limitation*" OR disabilit* OR "impaired	8,425,187

	wellbeing" OR "six-minute walk test" OR 6MWT OR Vo2max OR "Oxygen Consumption" OR fitness OR "activities of daily living")) OR AB=("Chronic diseases" OR "Chronic illness" OR "Chronic conditions" OR "Type 2 diabetes" OR "Diabetes Mellitus, Type 2" OR "cardiovascular disease*" OR CVD OR "coronary heart disease" OR "Coronary Disease" OR "ischaemic heart diseases" OR "Heart failure" OR "angina pectoris" OR "Coronary artery Disease" OR "Myocardial infarction" OR "thromboembolic diseases" OR Preeclampsia OR stroke OR "cerebrovascular diseases" OR hypertens* OR metabolic syndrome OR cancer OR "High Blood Pressure" OR "Fatty Liver" OR NAFLD OR "Hepatic dysfunction" OR Dyslipid?emia OR "Insulin Resistance" OR "Chronic obstructive pulmonary disease" OR "impaired glucose tolerance" OR CKD OR "Kidney disease*" OR "renal insufficiency" OR Gout OR osteoarthritis OR Weight-related problem* OR "Weight-related complication*" OR "obesity-related" OR "Mental disorder*" OR Depression OR anxiety OR "Obstructive sleep apnea" OR OSA OR "Polycystic ovary syndrome" OR "Gastro-oesophageal reflux disease" OR GORD OR "binge eating disorder" OR "disordered eating behaviour" OR "Stress-related disorder*" OR stress OR "Functional limitation*" OR "mobility restriction*" OR Gout OR "physical limitation*" OR disabilit* OR "impaired wellbeing" OR "six-minute walk test" OR 6MWT OR Vo2max OR "Oxygen Consumption" OR fitness OR "activities of daily living")	
5	(TI= (Cohort Stud* OR Follow-Up Stud* OR Longitudinal Stud* OR Prospective Stud* OR Retrospective Stud* OR Cross Sectional Stud* OR Observation* Stud* OR "Mendelian Randomi#ation")) OR AB=(Cohort Stud* OR Follow-Up Stud* OR Longitudinal Stud* OR Prospective Stud* OR Retrospective Stud* OR Cross Sectional Stud* OR Observation* Stud* OR "Mendelian Randomi#ation")	3,904,528
6	#1 AND #2 AND #3 AND #4 AND #5and English (Languages)	828

Google Scholar

(Adult OR Aged 18 and Older OR Older Adult OR adult population OR adult patients OR adulthood) AND (Obesity OR Overweight OR Adiposity OR Overnutrition OR Nutrition Disorder) AND (Fat free mass OR Lean body OR Lean mass OR Lean tissue OR Muscle mass OR Skeletal muscle OR Muscle mass index OR Appendicular mass OR Body composition) AND (Obesity-related complications)	1036

Supplementary Table S2: List of excluded studies with reasons

PICOS domains	Reasons for exclusion	References
Population	Did not meet eligibility criteria for population: participants aged <18 years, studies including participants without overweight or obesity (BMI <25 kg/m²), or mixed-BMI studies without separate analyses for participants with overweight/obesity (n = 9)	[1-9]
Exposure	Did not meet eligibility criteria for exposure: no measure of fat-free mass (FFM), skeletal muscle mass, appendicular lean mass, or related derived indices reported (n = 26)	[10-35]
Outcome	Did not meet eligibility criteria for outcomes: studies did not report eligible weight-related complications, or effect estimates could not be extracted or calculated (n= 39)	[36-74]
Study design	Did not meet eligibility criteria for study design: randomized controlled trials (RCTs), case-control studies, review articles, or other non-eligible study designs (n = 11)	[75-85]

Supplementary Table S3: Characteristics and summary of studies reviewed.

Study details	Population and setting	Independent variable, measures	Dependent variables, measures	Statistical analysis, results/effect estimates	Author's conclusion
**Authors:** Barsalani et al.,<br>**Year published:** 2013<br>**Study design:** Cross-sectional study<br>**Country:** Canada	**Sample size:**<br>N=231 postmenopausal women aged 46 to 75 years, mean ± SD BMI: 30.7±4.9 kg/m^2<br>**Inclusion criteria:**<br>• Apparently healthy individuals<br>• Body mass index<br>• (BMI) > 18.5 kg/m^2<br>• No physical incapacity<br>• No hormone replacement therapy for at least one year<br>• Weight stable (±2 kg) for the last six months<br>• Non-smoker, moderate drinker (max 15 g of alcohol/day)<br>• No medication affecting glucose and lipid metabolism.<br>• No menstruation for the past twelve months<br>**Exclusion criteria:**<br>• None stated<br>**Study Setting and population:**	• Appendicular lean body mass (LBM) index, measured using DXA and<br>• Calculated as appendicular LBM divided by height squared.	1. Plasma insulin<br>2. Insulin resistance, measured using (QUICKI)	**Statistical analysis**<br>• Pearson's correlation coefficients were used to quantify the relationship between variables.<br>• ROC analysis was used to determine the cut-off value of appendicular LBM index with optimum (equalled) sensitivity and specificity for insulin resistance.<br>• Adjusted for WC and age<br>**Results/effects estimates:**<br>1. Correlation between plasma insulin and LBM: $r = 0.48$, $p < 0.01$<br>2. Correlation between QUICKI and appendicular LBM index: $r = -0.45$, $p < 0.01$<br>3. ROC analysis: AUC = 0.724 for appendicular LBM index cut-off of 7.025 kg/m^2, based on QUICKI cut-off of < 0.357)	**Authors' conclusions:**<br>The authors concluded that: "Our results suggest that women who were postmenopausal and had sedentary lifestyles and an appendicular skeletal muscle mass index above 7.025 kg/m^2 may be at greater risk of insulin resistance."

	• Postmenopausal women aged 46 to 75 years from University Geriatric Institute of Sherbrooke and Université de Montréal				
**Authors:** Boo, S. H et al.,<br>**Year published:** 2018.<br>**Study design:** Cross-sectional study<br>**Country:** South Korea	**Sample size:**<br>N=102 men, aged over 60 years, mean ±SD BMI: 25.0±3.5 kg/m^2<br>**Inclusion criteria:**<br>• Men 60 years and older who underwent a health check-up.<br>**Exclusion criteria:**<br>• History of severe cardiovascular or respiratory disease<br>• Unstable angina, uncontrolled arrhythmias,<br>• Orthopaedic impairments affecting exercise performance<br>• Use of medications affecting body weight or composition<br>**Study Setting and population:**<br>• Community-dwelling men from tertiary medical institution.	• Appendicular skeletal muscle mass (ASM), measured using BIA<br>• Skeletal muscle mass index (SMI), calculated as ASM divided by the square of height.<br>• Sarcopenia grouping (SMI of ≤7.0 kg/m^2 vs. >7.0 kg/m^2)	1. VO2peak (mL/min)<br>2. VO2peak/weight (mL/kg/ min)<br>3. METpeak	**Statistical analysis**<br>• Pearson's correlation analysis was used to evaluate relationships between variables.<br>• Independent t-test was used to compare means between two groups<br>• Multiple linear regression was used to test for independent associations between variables<br>• Adjusted for fat mass but adjusted for age and nutritional status<br>**Results/effects estimates:**<br>• Pearson's correlation analysis<br>• VO2peak ($r = 0.597$, $p < 0.001$)<br>• VO2peak/weight ($r = 0.268$, $p = 0.024$) adjusted for body weight<br>• METpeak ($r = 0.269$, $p = 0.021$)<br>• Independent t-test between groups:<br>• Mean ±SD of VO2peak (mL/min) in sarcopenic = 1193±207 vs. non-sarcopenic = 1430±346, $p < 0.001$.<br>• Mean ±SD of VO2peak/weight (mL/kg/min) in sarcopenic = 17.6 ± 4.1 vs. non-sarcopenic = 19.8 ± 4.4, $p = 0.046$ adjusted for body weight	**Author's conclusions:**<br>The authors concluded that: "skeletal muscle mass might be closely associated with CRF."

				• Mean ±SD of METpeak in sarcopenic = 5.0 ± 1.2 vs. non-sarcopenic = 5.7 ± 1.3, p = 0.045<br>• Multiple linear regression analyses<br>• $\beta = 0.473$, $p < 0.001$	
**Authors:** Carvalho et al., **Year published:** 2020 **Study design:** Cross-sectional study **Country:** Brazil	**Sample size:** N= 689 adults, mean ±SD BMI: 27.0±4.5 kg/m^2 **Inclusion criteria:**<br>• Adults aged 20-59 years<br>**Exclusion criteria:**<br>• Pregnant women<br>• Bedridden individuals<br>• Those unable to undergo measurements or respond to the questionnaire<br>**Setting and population:**<br>• Adults of both sexes who live in urban area of the municipality of Viçosa-MG	• skeletal muscle mass, measured using DXA<br>• The skeletal muscle mass index (SMI) calculated as appendicular lean mass (ALM) adjusted for tertiles of ALM/BMI, kg/kg/m²)	1. Metabolic syndrome (MetS) defined by harmonized criteria	**Statistical analysis**<br>• Multivariable logistic regression for OR (95%CI), adjusted for covariates<br>• Adjusted for age, schooling, marital status, and lifestyle factors<br>**Results/effects estimates:**<br>1. SMI $_{BMI}$ (tertile 3 vs. ref. (tertile 1))<br>• Men: OR = 0.14 (95% CI: 0.05, 0.37)<br>• Women: OR = 0.34 (95% CI: 0.12, 0.94)	**Author's conclusions:** The authors concluded that: "In adults, the muscle mass assessed from the skeletal mass index adjusted for body weight and body mass index is inversely associated with metabolic syndrome in both sexes."
**Authors:** Choe et al., **Year published:** 2018 **Study design:** Cross-sectional study **Country:**	**Sample size:** N=1828, 26.6% with obesity (defined as BMI ≥ 25 kg/m^2) **Inclusion criteria:**<br>• Individuals visiting for routine health check-ups who underwent abdominal	• CT-measured skeletal muscle mass indices (SMI_{CT})<br>• Sarcopenia, defined as having a CT-measured skeletal muscle index (SMI-CT) that is 1 standard deviation below the sex-specific mean	1. Non-alcoholic fatty liver disease (NAFLD), defined as the presence of a fatty liver on ultrasonography in the absence of other possible causes of chronic liver diseases	**Statistical analysis:**<br>• Descriptives (proportions)<br>• Multiple logistic regression for OR (95%CI), adjusted for covariates<br>• Multivariate regression analysis of the risk of NAFLD in quartiles (adjusted for covariates (Model 3)<br>• Adjusted for age, sex, waist circumference, systolic blood pressure, and fasting glucose.<br>**Results/effects estimates:**	**Author's conclusions:** The authors concluded that: "This study shows that low muscle mass, which was precisely measured with CT, is significantly associated with

South Korea	ultrasonography and CT scanning<br>**Exclusion criteria:**<br>• Positive serologic marker for hepatitis B or C<br>• excessive alcohol intake (>30 g/day for males and >20 g/day for females)<br>• Other specific hepatic diseases<br>• History of malignant disease<br>**Setting and population:**<br>• Individuals visiting the centre for routine health check-ups at Seoul National University Hospital	value for a young healthy population, with cut-off values of 8.37 cm²/(kg/m²) for males and 7.47 cm²/(kg/m²) for females<br>• Mild sarcopenia is between 1 and 2 SD below the mean<br>• Severe sarcopenia is 2 or more SD below the mean (7.04 cm²/(kg/m²) for males and 6.12 cm²/(kg/m²) for females)<br>• No sarcopenia is defined as having an SMI CT above 8.37 cm²/(kg/m²) for males and above 7.47 cm²/(kg/m²) for females, as these are the cut-off values for sarcopenia.<br>• Quartiles:<br>o Males: Q1: ≤8.53 cm²/(kg/m²); Q2: 8.53-9.25 cm²/(kg/m²); Q3: 9.25-10.16		• Proportion of NAFLD for sarcopenic obesity vs nonsarcopenic obesity 35.9% vs. 26.8%)<br>• Multivariate regression analyses:<br>• OR= 1.51(95% CI: 1.15, 1.99); for mild sarcopenia vs. no sarcopenia:<br>• OR =1.45 (95% CI: 1.09, 1.92); for severe sarcopenia vs. no sarcopenia<br>• Multivariate regression analysis:<br>• Male: Q3 vs. ref. (Q4): OR = 1.09 (95% CI: 0.74, 1.61); Q2 vs. ref. (Q4): OR = 1.99 (95% CI: 1.33, 2.98); Q1 vs. ref. (Q4): OR = 1.78 (95% CI: 1.17, 2.72)<br>• Female: Q3 vs. ref. (Q4): OR = 1.61 (95% CI: 0.73, 3.71); Q2 vs. ref. (Q4): OR = 1.76 (95% CI: 0.83, 3.98); Q1 vs. ref. (Q4): OR = 2.39 (95% CI: 1.13, 5.37)	NAFLD, independent of other metabolic risk factors".

		cm²/(kg/m²); Q4: >10.16 cm²/(kg/m²)<br>o Females: Q1: ≤7.22 cm²/(kg/m²); Q2: 7.22-7.93 cm²/(kg/m²); Q3: 7.93-8.94 cm²/(kg/m²); Q4: >8.94 cm²/(kg/m²)			
**Authors:** Choe et al., **Year published:** 2023 **Study design:** Prospective cohort study **Country:** South Korea	**Sample size:** N= 5480 participants, mean ± SD BMI: 27.6 ± 3.1 kg/m²<br>**Inclusion criteria:**<br>• Adults aged 40-70 years from Ansung and Ansan communities<br>**Exclusion criteria:**<br>• Individuals with a history of atherosclerotic cardiovascular disease, heart failure, cancer, or thyroid disease; those with thyroid stimulating hormone levels lower than 0.4 IU/mL or higher than 5.0 IU/mL; individuals who reported steroid use or excessive alcohol intake (male >30 g/day, female >20 g/day); participants	• Muscle mass, measured using BIA<br>• Sex-specific quartiles of BMI-adjusted muscle mass were calculated, and low muscle mass was defined as the lowest quartile (Q1)	1. Incidence of NAFLD, defined as a hepatic steatosis index of ≥36<br>2. Incidence of NAFLD-related hepatic fibrosis, defined based on a fibrosis-4 (FIB-4) index of ≥1.3 in patients with underlying NAFLD	**Statistical analysis**<br>• Polynomial regression was used to calculate P- value for trends in the context of BMI-adjusted muscle mass quartiles<br>• Cox proportional hazard regression was used to assess the risk of developing NAFLD or hepatic fibrosis for HR (95% CI), adjusted for covariates<br>• Adjusted for age, sex, metabolic syndrome and WC<br>**Results/effects estimates:**<br>1. Polynomial regression in each quartile: Q1: 30.0%, Q2: 27.1%, Q3: 22.7%, Q4: 15.3%; P < 0.001<br>2. Cox proportional hazard analysis:<br>• Q1: HR = 1.65 (95% CI: 1.33, 2.05)<br>• Q2: HR = 1.53 (95% CI: 1.26, 1.87)<br>• Q3: HR =1.26 (95% CI: 1.04, 1.53)<br>• Q4: HR 1 (reference)<br>• Per 1Q: HR = 1.18(1.11, 1.27)<br>3. Incidence of hepatic fibrosis in each quartile: Q1: 51.8%, Q2: 44.7%, Q3: 42.6%, Q4: 41.0%; P for trend = 0.006	**Author's conclusions:** The authors concluded that: "Both lower muscle mass index and genetic risk variants are important contributors to the development of NAFLD in patients already diagnosed with NAFLD,"

	without bioelectrical impedance analysis; and participants without genotype information were excluded.<br>**Setting and population:**<br>• Adults aged 40-70 years from Ansung and Ansan communities, Korea			4. Cox proportional hazard analysis for hepatic fibrosis:<br>• HR =1.02 (95% CI: 0.85, 1.22)	
**Authors:** Kevin et al.,<br>**Year published:** 2012<br>**Study design:** Cross-sectional study<br><br>**Country:** United States	**Sample size:**<br>N= 39 females, mean ± SD BMI: 33.8±1.2 kg/m^2<br>**Inclusion criteria:**<br>• Women with PCOS based on NIH criteria, aged 18-42 years, BMI 20-50 kg/m²<br>**Exclusion criteria:**<br>• Use of birth control pills, insulin sensitizers, or lipid-lowering medications within 2 months prior to the study<br>**Setting and population:**<br>• Women with PCOS at Clinical and Translational Science Center Clinical Research Center, University of California, Davis	• Lean mass, measured using DEXA and<br>• Lean mass index (LMI), calculated as lean mass (kg) divided by height (m) squared	1. Insulin resistance, assessed using HOMA from the OGTT and FS-IVGTT<br>2. Insulin sensitivity, assessed using QUICKI for hepatic insulin sensitivity and the Matsuda index for peripheral insulin sensitivity	**Statistical analysis**<br>• Partial Pearson's correlation analysis between lean mass and insulin resistance and sensitivity parameters adjusted for fat mass<br>• Two-tailed t-tests between mean ± SD of high lean mass (52.8±1.8kg) vs. low lean mass (44.4±1.6kg) groups matched for fat mass (n=11)<br>• Adjusted for Fat mass<br><br>**Results/effects estimates:**<br>1. Correlations:<br>• HOMA: r=0.456, p = 0.014<br>• QUICKI: r=−0.522, p = 0.009<br>• ISI Matsuda: r=−0.289, P= 0.171<br>• SI: r= −0.360, P=0.109<br>2. Between group means<br>• HOMA: Mean ± SD high lean mass = 6.8 ± 2.1 vs. low lean mass = 3.9 ± 0.5, p = 0.225<br>• ISI Matsuda: high lean mass = 2.1 ± 0.4 vs. low lean mass = 3.0 ± 0.5, p = 0.113	**Author's conclusions:**<br>The authors concluded that: "These novel findings indicate that lean mass relates directly to insulin resistance in PCOS."

				• AUCGlucose 0–120: Mean ± SD high lean mass = 294 ± 14 vs. low lean mass = 253 ± 11, p = 0.034	
**Authors:** Fenger et al., **Year published:** 2017 **Study design:** Prospective cohort study **Country:** Denmark	**Sample size:** N=55,273, mean BMI males (n=26,307): 26.6 kg/m² vs. female (n=28,966): 25.6 kg/m² **Inclusion criteria:** • Age 50 to 64 years, • Living in Copenhagen or Aarhus • No previous cancer diagnosis **Exclusion criteria:** • Participants with an AF event before recruitment • Missing or insufficient lifestyle-related data **Setting and population:** • Individuals aged 50 to 64 years from the Danish Diet, Cancer, and Health study cohort from Copenhagen and Aarhus, Denmark	• Lean body mass, measured using BIA	1. Atrial fibrillation, defined as an inpatient or outpatient contact registered with a first-time primary diagnosis, using ICD-8 codes 427.93 and 427.94, and ICD-10 code I48	**Statistical analysis:** • Cox proportional hazards regression was used to examine the association between lean body mass and the risk of atrial fibrillation (adjusted for other arthrometric measures) • Adjusted for fat mass **Results/effects estimates:** 1. Cox proportional hazards regression: HR = 1.37 (95% CI: 1.33, 1.42)	**Author's conclusions:** The authors concluded that: "Lean body mass was the predominant anthropometric risk factor for AF, whereas no association was observed for either of the obesity-related anthropometric measures after adjustment for lean body mass."
**Authors:** Fornari et al., **Year published:** 2015 **Study design:**	**Sample size:** N= 426, mean ± SD BMI: 34.9 ± 6.1 kg/m² **Inclusion criteria:**	• Lean mass, measured using DXA and expressed as percentage of LM relative to total body mass	1. Insulin resistance, assessed using HOMA-IR 2. Inflammatory markers, assessed using fibrinogen levels	**Statistical analysis:** • Spearman's correlation (r) analysis was used to evaluate the relationship between lean mass percentage (LM%) and HOMA, as well as fibrinogen levels.	**Author's conclusions:** "The authors concluded that: Our results show for the first time that in

Cross-sectional study<br>**Country:** Italy	• Patients admitted for the diagnosis and therapy of obesity<br>**Exclusion criteria:**<br>• Chronic medical conditions<br>• Use of medications affecting bone metabolism<br>• Alterations in hormonal and nutritional status<br>• Patients taking Vitamin D supplementation<br>• Recent weight loss, prior bariatric surgery<br>**Setting and population:**<br>• Individuals with obesity from the Department of Experimental Medicine, Section of Medical Pathophysiology, Endocrinology and Nutrition, Sapienza University of Rome			• Adjusted for fat mass, demographics and lifestyle<br>**Results/effects estimates:**<br>1. Correlations<br>• HOMA: r^2= 0.039, p < 0.009<br>• fibrinogen level: r^2 = 0.1263, p < 0.0001	Individuals with obesity, higher amounts of lean mass are directly linked to a lower inflammatory profile and to better insulin sensitivity, but also to the presence of higher level of vitamin D and IGF-1. Moreover, these data suggest that higher levels of lean mass in people with obesity correlate with a better metabolic profile."
**Authors:** Itterman et al., **Year published:** 2019 **Study design:**	**Sample size:**<br>N=4476, mean ± SD BMI<br>• For SHIP group: 26.17 ± 3.85 kg/m²<br>• For KORA: 26.48 ± 3.93 kg/m²<br>• For CARLA: 26.90 ± 3.56 kg/m²	• Fat-free mass, calculated using a formula based on height and weight, derived from BIA analysis models.	1. Incident hypertension, defined as SBP ≥ 140 mmHg, DBP ≥ 90 mmHg, or intake of antihypertensive medication at follow-up	**Statistical analysis:**<br>• Confounder-adjusted linear regression was used for associations with changes in BP<br>• Logistic regression was used for incident hypertension and normalization of BP<br>• Adjusted for fat mass<br>**Results/effects estimates:**	**Author's conclusions:**<br>The authors concluded that: "Our data suggests that the well-established association between obesity and blood pressure levels

Population-based cohort study<br>**Country:**<br>Germany	• For PopGen: 25.83 ± 3.70 kg/m²<br>**Inclusion criteria:**<br>• Aged 21 to 82 years<br>• Not taking antihypertensive medication<br>• No type 2 diabetes mellitus or history of myocardial infarction at baseline and follow-up<br>**Exclusion criteria:**<br>• Pregnant women<br>• Patients with type 2 diabetes mellitus<br>• Myocardial infarction<br>• Stroke<br>• Cancer<br>• Use of antihypertensive medication<br>• Missing data<br>**Setting and population:**<br>• Participants from four population-based studies; (Northwest - PopGen, Northeast - SHIP, East - CARLA, South - KORA).		2. Changes in systolic and diastolic blood pressure, defined as the annual absolute change, calculated by dividing the absolute change between baseline and follow-up by the individual follow-up time in years. BP was measured three times, and the mean of the second and third measurements was used for analysis.	1. Annual change in SBP associated with FFM: β = 1.66 (95% CI: 1.31, 2.01)<br>2. Annual change in DBP associated with FFM: β = 1.18 (95% CI: 0.97, 1.40)<br>3. Associations of change in fat-free mass with incident hypertension Vs. normalization:<br>• Incident hypertension: OR = 3.26 (95% CI: 2.05, 5.19) vs. Incident normalization of BP: OR = 0.42 (95% CI: 0.25, 0.69)	might be more related to body composition rather than to total body weight per se. Our findings indicate gaining or losing FFM has substantial impact on the development or reversion of hypertension.”
**Authors:**<br>Kang et al.,	**Sample size:**<br>N=683, Obesity (%) in LSMM group (n = 75):	• Appendicular skeletal muscle mass (ASM), measured using BIA	1. NAFLD, defined as the presence of a fatty liver in the absence of any exclusion criteria,	**Statistical analysis:**<br>• Multivariate logistic regression analysis was used to evaluate the association between	**Author’s conclusions:**<br>The authors concluded that: “LSMM is

**Year published:** 2021 **Study design:** Cross-sectional study **Country:** South Korea	90.7% vs. non-LSMM group (n = 608): 66.3%, mean ±SD BMI: 28.0±2.8 kg/m^2 **Inclusion criteria:** • Patients with ultrasound-confirmed NAFLD who underwent carotid ultrasonography **Exclusion criteria:** • No evidence of fatty liver on abdominal ultrasound • Positive serologic findings for hepatitis B or C • History of excessive alcohol consumption **Setting and population:** • Patients with NAFLD from Health promotion center at Yeungnam University Hospital	• LSMM, defined as appendicular skeletal muscle mass divided by body mass index (ASM/BMI), with thresholds of <0.789 in males and <0.512 in females based on the definition of foundation for the National Institutes of Health	determined by increased echogenicity of the liver parenchyma, deep beam attenuation, and blurring of intrahepatic vessels. 2. Increased carotid intima–media thickness (cIMT), defined as an increase in mean cIMT by ≥1.0 mm 3. Presence of carotid plaques; defined as a cIMT ≥ 1.5 mm and the presence of focal protrusion into the arterial lumen that is at least 50% larger compared to the adjacent lumen; Carotid atherosclerosis, defined by increased cIMT and presence of carotid plaque.	LSMM and increased cIMT and carotid plaque • Adjusted for WC, and hyperuricemia **Results/effects estimates:** 1. Logistic regression analysis for patients with NAFLD: • Increased cIMT: OR = 2.26 (95% CI: 1.26, 4.04) • Carotid plaque: OR = 2.74 (95% CI: 1.30, 5.78) 2. Logistic regression analysis for patients with obesity and NAFLD: • Increased cIMT: OR = 2.44 (95% CI: 1.29, 4.60) • Carotid Plaque: OR = 2.70 (95% CI: 1.18, 6.16)	associated with an increased risk of carotid atherosclerosis in patients with NAFLD independent of metabolic risk factors. Considering that carotid atherosclerosis is a predictor of future CVD events, physicians need to assess the presence of carotid atherosclerosis, as well as SMM, in patients with NAFLD.”
**Authors:** Koo et al., **Year published:** 2015 **Study design:**	**Sample size:** N= 1947, mean ± SD BMI: 25.8 ± 3.0 kg/m² for males; 27.9 ± 3.4 kg/m² for females **Inclusion criteria:**	• ASM, measured using DXA and calculated as the sum of the muscle mass in the arms and legs.	1. Metabolic syndrome (MetS), defined by the National Cholesterol Education Program Adult Treatment Panel III (NCEP ATP III) criteria, having three or	**Statistical analysis:** • Independent-sample t test was used to compare obesity-related factors between the lowest quintile and the higher quintiles for muscle mass indices • Chi-squared test was used for categorical variable	**Author’s conclusions:** The authors concluded that: “The relationship between low muscle mass and MetS was different according to

Cross-sectional study<br>**Country:**<br>South Korea	• Individuals aged 70 or older<br>**Exclusion criteria:**<br>• Subjects not amenable for body composition analysis by DXA or the components of MetS<br>**Setting and population:**<br>• Older individuals from the Korean population	• Low muscle mass (LMM), defined using three indices:<br>✓ ASM/Ht² below 2 SD of the sex-specific mean for healthy young adults, with cut-off points of 6.093 kg/m² for men and 4.169 kg/m² for women.<br>✓ ASM/Wt below 2 SD of the sex-specific mean for healthy young adults, with cut-off points of 27.2% for men and 21.3% for women.<br>✓ The lowest quintile of the distribution of the residual, which accounts for height and fat mass.	more of these criteria: (i) central obesity (waist circumference ≥90 cm for men and 85 cm for women); (ii) systolic blood pressure ≥130 mmHg or diastolic blood pressure ≥85 mmHg or antihypertensive medication use; (iii) high-density lipoprotein cholesterol <1.04 mM for men and 1.29 mM for women; (iv) triglyceride >1.69 mM; and (v) elevated fasting blood glucose ≥6.11 mM or taking hypoglycaemic agents.	• Logistic regression was used to assess the influence of the ASM on MetS adjusted for covariates<br>• Adjusted for age, height, and total fat mass<br>**Results/effects estimates:**<br>1. Between groups:<br>• Men with low muscle mass: 22/30 vs. normal muscle mass: 112/205; OR=2.28 (95% CI: 0.97, 5.37)<br>• Women with low muscle mass: 56/81 vs. normal muscle mass: 253/370; OR =1.04 (95% CI: 0.62, 1.74)<br>2. Logistic regression analysis:<br>• Men: OR= 1.21 (95% CI: 0.94, 1.54)<br>• Women: OR = 1.28 (95% CI: 1.07, 1.54)<br>3. Logistic regression for ASM and each component of MetS<br>Men<br>• C-WC: OR = 4.32 (95% CI: 3.02, 6.17)<br>• C-DM: OR = 0.74 (95% CI: 0.60, 0.91)<br>• C-BP: OR = 0.93 (95% CI: 0.78, 1.12)<br>• C-TG: OR = 1.01 (95% CI: 0.83, 1.2)<br>• C-HDL: OR = 1.14 (95% CI: 0.93, 1.40)<br>Women:<br>• C-WC: OR = 3.01 (95% CI: 2.30, 3.93)<br>• C-DM: OR = 1.06 (95% CI: 0.89, 1.26)<br>• C-BP: OR = 1.05 (95% CI: 0.90, 1.24)<br>• C-TG: OR = 0.89 (95% CI: 0.75, 1.04)<br>• C-HDL: OR = 1.09 (95% CI: 0.94, 1.28)	the various muscle mass indices.<br>After controlling for the influence of fat mass, decreased muscle<br>mass was not an independent risk factor for MetS."

**Authors:** Larsen et al., **Year published:** 2016 **Study design:** Prospective cohort study **Country:** United States	**Sample size:** N=2166 "older adults, mean ±SD BMI: 29.0±4.2 kg/m^2<br><br>**Inclusion criteria:**<br>• Subjects aged 70-79 years<br>• Well-functioning residing in Pittsburgh, PA or Memphis<br>**Exclusion criteria:**<br>Individuals with T2DM at baseline were excluded from this study<br>**Setting and population:**<br>• Older adults aged 70-79 who were white Medicare beneficiaries and black community residents at Pittsburgh, PA and Memphis, TN	• Total body lean mass (kg) measured using DXA	1. Incident diabetes, defined as fasting glucose levels ≥126 mg/dL, a physician's diagnosis of diabetes, and/or the use of hypoglycemic medication.	**Statistical analysis:**<br>• A staged multivariate Cox regression model was used to determine the association between total lean mass and incident diabetes, as well as the risk of incident diabetes associated with total lean mass.<br>• Adjusted for BMI, visceral fat area, and total body fat<br>**Results/effects estimates:**<br>1. Cox regression analysis:<br>• Men: HR = 0.84 (95% CI 0.66, 1.07)<br>• Women: HR = 0.96 (95% CI 0.75, 1.25)<br>2. Cox regression analysis for risk of incident diabetes associated with total lean mass<br>• Men: HR = 0.87 (0.69, 1.09)<br>• Women: HR = 1.10 (0.89, 1.36)	**Author's conclusions:** The authors concluded that: "Greater muscle area is associated with lower risk of incident diabetes for older normal weight women, but not for men or overweight women."
**Authors:** LeCroy et al., **Year published:** 2020 **Study design:** Prospective cohort study **Country:**	**Sample size:** N= 6264, mean ± SD BMI:<br>Group 1: 27.7 ± 0.2 kg/m²<br>Group 2: 28.7 ± 0.2 kg/m²<br>Group 3: 28.8 ± 0.3 kg/m²<br>Group 4: 31.0 ± 0.2 kg/m²<br>based on change in relative FFM (group 1: the greatest decrease in %ΔFFM and group 4,	• FFM, measured using BIA<br>• Percent change in relative FFM (%DFFM), calculated as [(relative FFM baseline - relative FFM Visit 2)/ relative FFM baseline] *100	1. Incident prediabetes, defined as having a fasting glucose value in the range of 100–125 mg/dL, a post-OGTT glucose value of 140–199 mg/dL, or an HbA1c in the range of 5.7–6.5% (39–48 mmol/mol).	**Statistical analysis:**<br>• Poisson regression was used to determine the incidence rate ratios of prediabetes and T2DM according to %DFFM, adjusted for covariates<br>• Multivariable linear regression was used to examine associations between change in %DFFM and changes in fasting glucose, HbA1c, and HOMA-IR	**Author's conclusions:** The authors concluded that: "Reducing loss of FFM during adulthood may reduce prediabetes risk (primarily insulin resistance), particularly among individuals with overweight/obesity."

United States	greatest increase in %ΔFFM)<br>**Inclusion criteria:**<br>• Hispanic/Latino adults aged 18-74 years who completed Visit 2 without T2DM at baseline<br>**Exclusion criteria:**<br>• Subjects who are not living at home<br>• Planning to move soon<br>• Physically unable to attend clinic examination<br>• Unable to complete questionnaires in English or Spanish<br>• Pregnant women<br>**Setting and population:**<br>• Hispanic/Latino adults aged 18-74 years from Bronx, New York; Chicago, Illinois; Miami, Florida; San Diego, California.		2. Incident T2DM, defined according to American Diabetes Association criteria: defined as having a fasting glucose value ≥126 mg/dL, a post OGTT glucose value ≥200 mg/dL, an HbA1c ≥ 6.5% (≥ 48 mmol/mol), or use of diabetic medication.<br>3. Mean changes in glucose and insulin measures based on percent changes in relative FFM	• Adjusted for % change in FMI, % change in BMI and % change in WC<br>**Results/effects estimates:**<br>1. Poisson regression for incident Prediabetes:<br>• Group 1 (greatest % decrease in relative FFM): IRR = 0.98 (95% CI: 0.73, 1.33)<br>• Group 2: IRR = 0.95 (95% CI: 0.72, 1.25)<br>• Group 3: IRR = 0.94 (95% CI: 0.71, 1.24)<br>• Group 4 (greatest % increase in relative FFM): Reference group (IRR = 1.00)<br>2. Poisson regression models for incident T2DM<br>• Group 1: IRR = 1.13 (95% CI: 0.69, 1.84)<br>• Group 2: IRR = 1.30 (95% CI: 0.83, 2.02)<br>• Group 3: IRR = 1.09 (95% CI: 0.76, 1.55)<br>• Group 4: Reference group (IRR = 1.00)<br>3. Multivariable linear regression for ΔFasting glucose (mg/dL):<br>• Group 1: β= -0.13 (95% CI: -1.45, 1.20)<br>• Group 2: β= -0.40 (95% CI: -1.72, 0.93)<br>• Group 3: 0.51 (95% CI: -0.71, 1.72)<br>• Group 4: Reference group (0.00)<br>4. Multivariable linear regression for ΔHbA1c (%):<br>• Group 1: 0.01 (95% CI: -0.03, 0.05)<br>• Group 2: 0.01 (95% CI: -0.03, 0.05)<br>• Group 3: 0.02 (95% CI: -0.01, 0.06)<br>• Group 4: Reference group (0.00)<br>5. Multivariable linear regression for ΔHOMA-IR:<br>• Group 1: 0.77 (95% CI: 0.45, 1.10)	

				• Group 2: 0.51 (95% CI: 0.22, 0.80)<br>• Group 3: 0.40 (95% CI: 0.12, 0.67)<br>• Group 4: Reference group (0.00)	
**Authors:**<br>Lee et al.,<br>**Year published:**<br>2018<br>**Study design:**<br>Retrospective cohort study<br>**Country:**<br>Republic of Korea	**Sample size:**<br>N=9,033, mean BMI: males: Mean BMI = 26.8 kg/m² vs. females: Mean BMI = 26.7 kg/m²<br>**Inclusion criteria:**<br>• Metabolically healthy according to Wildman criteria<br>• Aged ≥30 years<br>**Exclusion criteria:**<br>Individuals with history of the following diseases<br>• History of CVD<br>• Cancer at baseline or diagnosed with incident cancer between baseline and follow-up<br>• Hyperthyroidism or Hypothyroidism,<br>• Chronic kidney disease,<br>• Hypoalbuminemia<br>**Setting and population:**<br>• Individuals aged ≥30 years, who underwent a routine health evaluation at the Asan Medical Center (AMC, Seoul, Republic of Korea)	• Muscle mass, measured using BIA<br>• ASM, calculated as the sum of lean muscle mass in the arms and legs<br>• A weight-adjusted skeletal muscle mass index (ASM/weight), used to assess relative muscle mass	1. Progression from metabolically healthy to metabolically unhealthy phenotype, defined as ≥1 metabolic risk factor according to Wildman criteria	**Statistical analysis:**<br>• Multivariate logistic regression was used to assess the odds ratio for the progression to a metabolically unhealthy phenotype after adjusting for covariates<br>• Adjusted for adiposity, metabolic health, and lifestyle<br>**Results/effects estimates:**<br>1. Multivariate logistic regression for the progression to a metabolically unhealthy phenotype<br>Men with Obesity:<br>• Q1: Reference (1.00)<br>• Q2: OR = 1.04 (95% CI: 0.74, 1.46)<br>• Q3: OR = 0.91 (95% CI: 0.64, 1.28)<br>• Q4: OR = 0.86 (95% CI: 0.60, 1.23)<br>Women with Obesity:<br>• Q1: Reference (1.00)<br>• Q2: OR = 0.65 (95% CI: 0.33, 1.30)<br>• Q3: OR = 0.86 (95% CI: 0.43, 1.72)<br>• Q4: OR = 0.69 (95% CI: 0.34, 1.42)	**Author's conclusions:**<br>The authors concluded that: "Greater skeletal muscle mass at baseline is significantly associated with maintenance of metabolically healthy status, especially in individuals without obesity."

**Authors:** Machado et al.,<br>**Year published:** 2021<br>**Study design:** Observational, prospective study<br>**Country:**<br>**Country:** Netherlands	**Sample size:**<br>N= 469, mean ± SD BMI: overweight group, 27.7 ± 1.5 kg/m^2 vs. obesity group, 34.4 ± 2.9 kg/m^2<br>**Inclusion criteria:**<br>• Diagnosis of COPD according to GOLD strategy<br>• Referral for pulmonary rehabilitation at CIRO<br>• No exacerbation at least 4 weeks prior<br>**Exclusion criteria:**<br>• History of other lung diseases<br>• Lung surgery<br>• Malignancy within the last five years<br>• BMI < 18.5 kg/m^2<br>**Setting and population:**<br>• Patients with COPD at CIRO, Horn, the Netherlands	• FFMI, calculated by dividing FFM by height squared<br>• ASMI calculated by summing lean mass for each extremity and dividing by height squared	1. Six-minute walking distance (6MWD)<br>2. VO2max (ml·min^{-1})<br>3. Anxiety and depression (measured by Hospital Anxiety and Depression Scale)	**Statistical analysis:**<br>• Student's t-test was used to compare means of normally distributed continuous variables for two groups<br>• Mann-Whitney U-test was used to compare medians of non-normally distributed variables for two groups<br>• Chi-squared test was used to assess associations between categorical variables<br>**Results/effects estimates:**<br>1. Student's t-test analysis for low FFMI vs. normal<br><u>Overweight Group (n=157)</u><br>• 6MWD (m): Mean ± SD Low FFMI: 414 ±126 vs. Normal FFMI: 434 ± 118<br>• 6MWD, %predicted: Mean ± SD Low FFMI: 67 ± 17 vs. Normal FFMI: 70 ± 17<br>• VO_2max (ml·min^{-1}): Median (Interquartile range) Low FFMI: 1088 (900–1291) vs. Normal FFMI: 1108 (872–1349)<br>• VO2max, %predicted: Median (Interquartile range) Low FFMI: 58 (48–77) vs. Normal FFMI: 67 (51–86)<br>• HADS Anxiety score: Median (Interquartile range) Low FFMI: 6 (4–10) vs. Normal FFMI: 8 (5–13)<br>• HADS Depression score: Median (Interquartile range) Low FFMI: 7 (4–10) vs. 7(4–11)<br><u>Obesity Group (n=106)</u><br>• 6MWD (m): Mean ± SD Low FFMI: 430 ± 118 vs. Normal FFMI: 402 ± 108	**Author's conclusions:**<br>The authors concluded that: "Approximately half of the individuals with overweight and obesity with COPD have low muscle mass when applying age-gender-BMI-specific cut-offs. Low muscle mass is associated with worse functional outcomes in overweight<br>and participants with obesity COPD."

				• 6MWD, % predicted: Mean ± SD Low FFMI: 69 ± 16 vs. Normal FFMI: 70 ± 17<br>• VO2max (ml·min^{-1}): Median (Interquartile range) Low FFMI: 1267 (974–1542) vs. Normal FFMI: 1126 (892–1368)<br>• VO2max, % predicted: Median (Interquartile range) Low FFMI: 64 (54–75) vs. Normal FFMI: 81 (55–112), *P* < 0.05<br>• HADS anxiety score: Median (Interquartile range) Low FFMI: 8 (4–12) vs. Normal FFMI: 7 (5–12)<br>• HADS Depression score: Median (Interquartile range) of Low FFMI: 6 (4–11) vs. Normal FFMI: 8 (5–10)<br>1. Comparisons of outcomes between patients with low ASMI vs. normal<br>Overweight Group (n=156):<br>• 6MWD: Mean ± SD Low ASMI: 391 ± 122 vs. Normal ASMI: 471 ± 110, *P* < 0.001<br>• 6MWD, % predicted: Mean ± SD Low ASMI: 64 ± 17 vs. Normal ASMI: 76 ± 15, *P* < 0.001<br>• VO2max (ml·min^{-1}): Median (Interquartile range) Low ASMI: 1060 (873–1303) vs. Normal ASMI: 1156 (900–1402)<br>• VO2max (% predicted): Median (Interquartile range) Low ASMI: 56 (46–76) vs. Normal ASMI: 67 (53–88), *P* < 0.01.	

				• HADS anxiety Score: Median (Interquartile range) Low ASMI: 7 (5–11) vs. Normal ASMI: 6 (5–12)<br>• HADS depression score: Median (Interquartile range) Low ASMI: 8 (4–11 vs. Normal ASMI: 7 (4–10)<br>Obesity Group (n=103):<br>• 6MWD (m): Mean ± SD Low ASMI: 356 ± 97 vs. Normal ASMI: 426 ± 114, $P < 0.05$<br>• 6MWD, % predicted: Mean ± SD Low ASMI: 59 ± 15 vs. Normal ASMI: 71 ± 16, $P < 0.01$.<br>• VO2max (ml·min^{-1}): Median (Interquartile range) Low ASMI: 958 (818–1147) vs. Normal ASMI: 1230 (995–1501), $P < 0.05$<br>• VO2max, % predicted: Median (Interquartile range) Low ASMI: 55 (46–70) vs. Normal ASMI: 67 (55–97)<br>• HADS anxiety score: Median (Interquartile range) Low ASMI: 11(5–13) vs. Normal ASMI: 7 (5–11), $P < 0.05$<br>• HADS depression score: Median (Interquartile range) Low ASMI: 6(4–14) vs. Normal ASMI: 8 (4–10)	
**Authors:** Moreno et al., **Year published:** 2015 **Study design:**	**Sample size:** N= 421, mean ±SD BMI: 28.0±3.8 kg/m^2<br>**Inclusion criteria:**<br>• Age 30 to 65 years<br>• Ability to understand study procedures<br>**Exclusion criteria:**	• Lean mass, measured using DEXA	1. Carotid intima-media thickness (c-IMT), measured using high resolution ultrasonography	**Statistical analysis:**<br>• ANOVA was used to assess differences of variables across quartiles of lean mass<br>• Multiple linear regression stepwise analysis was used<br>• Adjusted for age, blood pressure, BMI, waist<br>**Results/effects estimates:**	**Author's conclusions:** The authors concluded that: "Lean mass, and not fat mass, might contribute to increase c-IMT in 47 patients with obesity independently of

Cross-sectional study<br>**Country:** Spain	• Systemic disease<br>• Recent infection<br>• Serious chronic illness<br>• >20 g ethanol intake per day<br>• Medications affecting insulin action<br>**Setting and population:**<br>• Individuals with obesity (BMI > 30 kg/m²), and small subset of participants without obesity for comparison, multicenter FLORINASH Project.			1. ANOVA for quartiles of lean mass:<br>Women (Mean ± SD)<br>• < 46.1 kg: Mean ± SD= 0.55 ± 0.15<br>• 46.1-51.8 kg: Mean ± SD = 0.64 ± 0.16<br>• 51.8-57.3 kg: Mean ± SD = 0.62 ± 0.16<br>• > 57.3 kg: Mean ± SD = 0.67 ± 0.15<br>• Significance level: p < 0.0001<br>Men<br>• < 60.9 kg: Mean ± SD = 0.58 ± 0.14<br>• 60.9-68.0 kg: Mean ± SD = 0.66 ± 0.15<br>• 68.0-75.3 kg: Mean ± SD = 0.69 ± 0.15<br>• > 75.3 kg: Mean ± SD = 0.75 ± 0.16<br>• Significance level: p < 0.0001<br>2. Multiple linear regression stepwise analyses:<br>• β = 0.253, p <0.0001	classical atherosclerotic risk factors."
**Authors:** Muster et al.,<br>**Year published:** 2021<br>**Study design:** Cross-sectional study<br><br>**Country:** Austria	**Sample size:**<br>N= 412, mean ± SD = BMI: 27.3 ± 4<br>**Inclusion criteria:**<br>• Current or previously diagnosed LEAD (Rutherford Classification 2-4)<br>• Documented luminal stenosis >70% on ultrasound or angiography, or a history of endovascular or surgical revascularization.<br>**Exclusion criteria:**	• Lean mass, calculated as weight (kg)–fat mass (kg)	1. Arterial stiffness, measured by AIx and cfPWV	**Statistical analysis:**<br>• Linear regression analysis was used for arterial stiffness and lean mass according to sex:<br>• Adjusted for BMI<br>**Results/effects estimates:**<br>1. Linear regression analysis:<br>• AIx in male: β = -0.28 (95% CI: -0.48, 0.09)<br>• AIx in female: β = -0.41 (95% CI: -0.92, 0.11)<br>• cfPWV in male: β = -0.01 (95% CI: -0.06, 0.03)<br>• cfPWV in female: β = -0.02 (95% CI: -0.16, 0.12)	**Author's conclusions:**<br>The authors concluded that: "For patients with peripheral arterial disease, our data show that lower lean mass in male patients is associated with increased arterial stiffness as measured by the Aix."

	• Life expectancy <6 months<br>• Unstable coronary artery disease or cerebrovascular disease<br>• Uncontrolled diabetes<br>• Pregnancy<br>• Age <18<br>**Setting and population:**<br>• Predominantly male (72.09%), mean ± SD age 63.3 ± 10.1 years at the outpatient department at the Division of Angiology, Medical University of Graz				
**Authors:** Nguyen et al., **Year published:** 2022. **Study design:** Cohort study **Country:** Taiwan	**Sample size:** N=119, mean ± SD = BMI: 42.2 ± 7.0 kg/m²<br>**Inclusion criteria:**<br>• T2DM diagnosis with poor glycaemic control under medication<br>• No history of bariatric surgery<br>• Age over 18 years<br>**Exclusion criteria:**<br>• Pregnant women<br>• acquired end-organ damage	• FFM and muscle mass, measured using multiple-frequency BIA<br>• SM gain (%), calculated as 12-month SM% - baseline SM%<br>• FFM gain (%), calculated as 12-month FFM % - baseline FFM %	1. Type 2 diabetes remission, defined as HbA1c < 5.7% or FBG < 100 mg/dL and no regular T2D medication	**Statistical analysis:**<br>• Multivariate logistic regression was used to assess the association between changes in body composition (FFM gain, and SM gain) and type 2 diabetes remission 12 months post-laparoscopic sleeve gastrectomy (LSG)<br>• ROC analysis was used to determine the discriminatory power of fat-free mass (FFM) gain and skeletal muscle (SM) gain percentages in predicting diabetes remission 12 months post-LSG.<br>• Adjusted for age and sex<br>**Results/effects estimates:**<br>1. Logistic regression analysis for FFM:	**Author's conclusions:** The authors concluded that: "The T2DM remission rate significantly increased 16% and 26% for each additional percentage of FFM and SM gain one year after LSG, which only happened in males. Increased FFM and SM were remarkably associated with T2DM remission in males, but

	• C-peptide level < 0.2 ng/mL<br>**Setting and population:**<br>• Patients with T2DM and obesity at Taipei Medical University Hospital, Taiwan			• Total participants FFM gain (%): OR = 1.14 (95% CI: 1.04, 1.26)<br>• Female: OR= 1.09 (95% CI: 0.95, 1.24)<br>• Males: OR = 1.16 (95% CI: 1.02, 1.32)<br>2. Logistic Regression analysis for SM:<br>• Total participants SM gain (%): OR = 1.16 (95% CI: 1.01, 1.34)<br>• Females: OR = 1.02 (95% CI: 0.84–1.25)<br>• Males: OR = 1.26 (95% CI: 1.02, 1.55)<br>1. ROC curves analysis for percentage of FFM and SM gain predicting diabetes remission<br>• FFM gain (%): AUC = 0.807 (95% CI: 0.682, 0.931)<br>• SM gain (%): AUC = 0.803 (0.665, 0.940)	evidence lacks for females."
**Authors:** Ozmen et al., **Year published:** 2009 **Study design:** Cross-sectional study **Country:** Turkey	**Sample size:**<br>N = 110 Caucasian adults, mean ±SD BMI: 32.0±3.6 kg/m^2<br>**Inclusion criteria:**<br>• Subjects with CKD referred for GFR measurement by technetium-99m DTPA renography.<br>**Exclusion criteria:**<br>• Patients with acute renal failure<br>• Cirrhosis, trauma or burns	• Lean body mass (LBM) measured, using leg-to-leg bioimpedance<br>• LBM/SCr, calculated as ratio of ratio of LBM to SCr	1. CKD, defined according to the criteria of the Dialysis Outcomes Quality Initiative (DOQI), referred for GFR measurement by technetium-99m diethylenetetramine-pentaacetic acid (99mTc-DTPA) renography	**Statistical analysis:**<br>• Multiple linear regression was used for LBM/SCr as accurate predictor of GFR estimation in groups of overweight and obesity:<br>• Stepwise linear regression analysis<br>• Adjusted for age, gender, serum albumin, proteinuria and fat mass<br>**Results/effects estimates:**<br>Multiple linear regression analysis:<br>• Overweight group: r^2= 0.804<br>• Obesity group: "R^{2}" = 0.779	**Author's conclusions:** The authors concluded that: "LBM measured by bioimpedance was an independent predictive factor of GFR in individuals with obesity or overweight and added clinically important diagnostic value to 1/SCr."

	• Unstable kidney function or renal transplant<br>• Pregnant patients<br>• Those on dialysis.<br>**Setting and population:**<br>• Patients categorized by BMI into underweight, normal weight, overweight, and groups with obesity in Turkey				
**Authors:**<br>Park et al.,<br>**Year published:**<br>2022<br>**Study design:**<br>Retrospective cross-sectional study<br>**Country:**<br>South Korea	**Sample size:**<br>N=2,561, mean ± SD BMI: Class I Sarcopenia group = 25.6 ± 2.4 kg/m² vs. Class II Sarcopenia group = 27.9 ± 3.4 kg/m²<br>**Inclusion criteria:**<br>• Asymptomatic Korean subjects who underwent BIA and CAVI<br>**Exclusion criteria:**<br>• Other ethnicities<br>• No BIA<br>• History of myocardial infarction<br>• Known angina<br>• History of stroke<br>**Setting and population:**<br>• Asymptomatic Korean individuals without known cardiovascular or	• ASM%, calculated as ASM/weight (kg) × 100<br>• Class I sarcopenia, defined as ASM% beyond 1 standard deviation (SD) and <2 SD<br>• Class II sarcopenia, defined as 2 SD below the gender-specific mean of healthy young adults	1. Arterial stiffness measured using cardio-ankle vascular index (CAVI)	**Statistical analysis:**<br>• Linear regression analysis was used to assess the relationship between CAVI and the predictor variables<br>• Multivariate regression model was used to assess the independent contribution of Class I and Class II sarcopenia on arterial stiffness after adjusting for cofounders<br>• Adjusted for age, sex, BMI, HTN, DM, hypercholesterolemia, and smoking<br>**Results/effects estimates:**<br>1. Linear regression analysis for ASM%, Class I and II Sarcopenia:<br>• ASM%: β = −0.024, SE = 0.006, p <0.001<br>• Class I Sarcopenia: "Estimate" = 0.238, SE = 0.046, p <0.001<br>• Class II Sarcopenia: "Estimate" = 0.433, SE = 0.080 p <0.001<br>2. Multivariate regression analysis<br>• Class I Sarcopenia: "Estimate" = 0.148, SE = 0.043, p = 0.001	**Author's conclusions:**<br>The authors concluded that:<br>"Low muscle mass is independently and significantly associated with increased CAVI, and should be considered when managing asymptomatic subjects to<br>assess the risk of atherosclerosis."

	cerebrovascular disease at Seoul National University Hospital Gangnam Centre.			• Class II Sarcopenia: "Estimate" = 0.304, SE = 0.073, p <0.001	
**Authors:** Peppa et al., **Year published:** 2014 **Study design:** Cross-sectional study **Country:** Greece	**Sample size:** N=150 Females, mean ± SD BMI: 29.6 ± 5.8 kg/m^2 **Inclusion criteria:** • Postmenopausal women reporting more than 1 year since last menstruation and/or follicle-stimulating hormone levels above 30 U/L **Exclusion criteria:** • Current or prior hormone replacement treatment • Overt cardiovascular disease, diabetes, hypertension or dyslipidaemia under treatment • Medications affecting body composition or cardiometabolic parameters • Uncontrolled thyroid disease • Orthopedic limitations • Age above 70 years	• LBMI, defined as total LBM (kg) • divided by height in meters squared (m^2)	1. Cardiometabolic risk parameters	**Statistical analysis:** • Partial correlation analysis was used to analyze the correlation between LBMI and cardiometabolic parameters. • Adjusted for age, mediterranean diet adherence, physical activity level, total fat mass and waist circumference **Results/effects estimates:** 1. Partial correlation coefficients: • SBP: r= 0.23, $P \leq 0.05$ • DBP: r = 0.21 • HOMA-IR: r = 0.21 • HDL-Cholesterol: r = -0.06, not significant • Triglycerides: r = 0.07, not significant • hs-CRP: r = 0.25, P≤0.05	**Author's conclusions:** The authors concluded that: "LBM is unfavourably associated with cardiometabolic risk factors in healthy postmenopausal women. Whether LBM, especially in arms, is associated with cardiometabolic health independently of central fat distribution in postmenopausal women."

	• Substantial body weight fluctuations in the previous 6 months<br>**Setting and population:**<br>• Nonsmoking postmenopausal women at Attikon University Hospital, Athens University Medical School, Athens, Greece				
**Authors:** Pramyothin et al.,<br>**Year published:** 2017<br>**Study design:** Observational cross-sectional study<br>**Country:** Thailand	**Sample size:**<br>N=552 participants with obesity, mean ±SD BMI: 27.0 ± 3.5 kg/m^2<br>**Inclusion criteria:**<br>• Participants with BMI ≥25 kg/m² seeking weight management<br>**Exclusion criteria:**<br>• Patients with metallic implants or pacemakers<br>**Setting and population:**<br>• Asian adults with obesity, Siriraj Hospital, Bangkok, Thailand	• FFM, measured using BIA<br>• FFMI, calculated as FFM divided by height squared	1. Metabolic Syndrome, defined as patients who presented with ≥3 of 5 abnormalities as defined by the 2009 harmonized criteria<br>2. T2DM, diagnosis was made in patients who either received medications for T2D, self-reported diabetes, or had a fasting plasma glucose ≥126 mg/dL and/or HbA1c ≥6.5%	**Statistical analysis:**<br>• Chi-square and Fisher exact tests were used to compare categorical variables across groups.<br>• Independent t tests and Mann- Whitney tests were used to compare quantitative variables in patients with vs. without MS:<br>• Multivariate logistic regression model was used to identify predictors of T2DM and MetS while adjusting for potential confounders.<br>• Adjusted for age, FFMI, WHR and WC<br>**Results/effects estimates:**<br>1. Independent t tests analyses for MS<br>Women (n = 370)<br>• Median (IQR) FFM (kg): MS: 50.0 (45.1, 55.7) vs. No MS: 49.8 (45.2, 54.9), p = 0.72<br>• Median (IQR) FFMI (kg/m²): MS: 20.1 (18.7, 22.0) vs. No MS: 19.7 (18.3, 21.4), p= 0.06<br>Men (n = 182):	**Author's conclusions:**<br>The authors concluded that: "Older age, central fat distribution, and— in contrast to previous findings—an increase in FFMI among women were independent predictors of adverse metabolic health in this cohort of middle-aged Asian adults with obesity."

				• Median (IQR) FFM (kg): MS: 70.0 (64.8, 76.9) vs. No MS: 69.2 (64.3, 75.3), p = 0.46<br>• Median (IQR) FFMI (kg/m²): MS: 23.4 (22.0, 25.9) vs. No MS: 23.5 (22.0, 25.5), p= 0.88<br>2. Independent t tests analyses for T2DM<br>Women (n = 370):<br>• Median (IQR) FFM (kg): T2DM: 49.8 (45.4, 54.3) vs. No T2DM: 49.9 (44.9, 55.5), p = 0.99<br>• Median (IQR) FFMI (kg/m²): T2DM: 20.2 (18.8, 22.1) vs. No T2DM: 19.9 (18.3, 21.5), p = 0.07<br>Men (n = 182):<br>• Median (IQR) FFM (kg): T2DM: 69.6 (65.2, 74.7) vs. No T2DM: 69.8 (64.3–76.9), p= 0.99<br>• Median (IQR) FFMI (kg/m²): T2D: 23.9 (21.9, 25.7) vs. No T2DM: 23.3 (22.0, 25.6), p = 0.89<br>3. Multivariate logistic regression analyses of FFMI for MS<br>• Women FFMI: OR = 1.12 (95% CI: 1.03, 1.22)	
**Authors:**<br>Shao et al.,<br>**Year published:**<br>2023<br>**Study design:**	**Sample size:**<br>N=4,981, mean ± SD = BMI: 26.37 ± 4.95, Q3 =29.33 ± 5.47, Q4 = 33.90 ± 6.94<br>**Inclusion criteria:**	• Lean body mass, measured using DEXA	1. Insulin resistance (IR), measured by HOMA-IR	**Statistical analysis:**<br>• Linear Regression was used to assess the relationship between total lean mass and HOMA-IR, adjusting for confounding variables<br>• Adjusted for sex, age, marital status, race, education, family income to poverty ratio, BMI, WC, hypertension, diabetes, total	**Author's conclusions:**<br>The authors concluded that: "Maintaining a proper ratio of lean body mass and visceral fat is beneficial for decreasing IR."

Cross-sectional study<br>**Country:** United States	• Individuals older than 18 years who have completed DXA<br>**Exclusion criteria:**<br>• Individuals with tumors,<br>• Lacking visceral fat and lean body mass information<br>• Those unable to calculate HOMA-IR variables<br>**Setting and population:**<br>• United States (NHANES 2011-2018)			cholesterol, triglyceride, ALT, and blood uric acid<br>**Results/effects estimates:**<br>1. Linear regression analysis for total lean mass with HOMA-IR:<br>• Per-SD increase in total lean mass: β= -0.03 (95% CI: -0.05, -0.02)<br>• Q2 vs. ref. (Q1): β = -0.06 (95% CI: -0.08, -0.03)<br>• Q3 vs. ref: β= -0.08 (95% CI: -0.11, -0.05)<br>• Q4 vs. ref: β= -0.10 (95% CI: -0.14, -0.06)	
**Authors:** Sizoo et al.,<br>**Year published:** 2022<br>**Study design:** Cohort and cross-sectional<br>**Country:** Netherlands	**Sample size:**<br>N= 127,616 for T2DM prevalence analysis, mean ±SD BMI: 27.0±4.5 kg/m^2 and 94,565 for incident T2DM analysis, mean ±SD BMI: 26.0±3.5 kg/m^2<br>**Inclusion criteria:**<br>• Adults aged 18-65 from the Lifelines cohort with complete data<br>**Exclusion criteria:**<br>• Renal impairment<br>• Kidney disease<br>• Missing urinary CER or BMI data<br>• Underweight status<br>**Setting and population:**	• Low muscle mass at baseline, defined by 24-hour urinary creatinine excretion rate (CER) of < -1 SD of mean, separately for males and females (vs ref. group of ≥ -1 SD of the mean CER), within overweight (n= 26,287) and obesity (n= 17,196) group<br>• Decreased muscle mass, defined as the difference between CER at baseline and CER 5 years (vs ref.	1. Prevalence of T2DM at baseline (by self-report)<br>2. Prevalence of T2DM at 5-years (by self-report)<br>3. Incidence of T2DM at 5 years (by self-report)	**Statistical analysis:**<br>• Descriptive statistics, n (%)<br>• Logistic regression for OR (95%CI) for association between baseline low muscle mass and T2DM prevalence, adjusted for all covariates ("Model 6").<br>• Adjusted for age and BMI, hip circumference and WC, grams of protein per 1000 kcal, and physical activity.<br>**Results/effects estimates:**<br>1. Baseline T2DM prevalence<br>Overweight group<br>• Male: low muscle mass = 141 (4.9%) vs. ref. = 555(2.4%); OR = 1.37 (95%CI: 1.11, 1.68)<br>• Female: low muscle mass = 138 (4.4%) vs. ref. = 435 (2.0%); OR = 1.47 (95%CI: 1.19, 1.82)	**Author's conclusions:**<br>The authors concluded that: "Both a low muscle mass and loss of muscle mass are associated with the prevalence and incidence of diabetes in the general population. This association is strongest in people with normal weight and weakens in people within higher BMI subgroups."

	• Adults aged 18-65 from North of the Netherlands	group, "no decrease") within overweight and obesity group		Obesity group<br>• Male: low muscle mass = 50 (10.9%) vs. ref. = 471 (6.5%); OR = 1.41 (1.00, 1.98)<br>• Female: low muscle mass = 98 (9.5%) vs. ref. = 693 (6.0%); OR = 1.24 (95%CI: 0.98, 1.59)<br>2. T2DM prevalence at 5-years<br>Overweight group<br>• Male: decreased muscle mass = 253 (2.8%) vs. ref. = 255 (2.5%); OR = 1.28 (95%CI: 0.99, 1.66)<br>• Female: decreased muscle mass = 218 (2.4%) vs. ref. = 226 (2.2%); OR = 1.02 (95%CI: 0.75, 1.37)<br>Obesity group<br>• Male: decreased muscle mass = 164 (6.5%) vs. ref. = 168 (5.6%); OR = 1.10 (95%CI: 0.82, 1.48)<br>• Female: decreased muscle mass = 271 (6.0%) vs. ref. = 248 (5.0%); OR = 1.23 (95%CI: 0.95, 1.58)<br>3. T2DM incidence in<br>Overweight group<br>• Male: decreased muscle mass = 49 (2.2%) vs. ref. = 259 (1.7%)<br>• Female: decreased muscle mass = 24 (1.1%) vs. ref. = 210 (1.4%)<br>Obesity group<br>• Male: decreased muscle mass = 39 (5.5%) vs. ref. = 188 (4.5%)<br>• Female: decreased muscle mass = 55 (3.9%) vs. ref. = 235 (3.3%)	

**Authors:**<br>Son et al.,<br>**Year published:**<br>2017<br>**Study design:**<br>Prospective cohort study<br>**Country:**<br>South Korea	**Sample size:**<br>N= 6895, mean ±SD BMI after median follow-up of 9.06 years: 26.6 ± 4.2 kg/m² for individuals who developed T2DM and 25.6 ± 3.8 kg/m² for those who did not develop T2DM<br>**Inclusion criteria:**<br>• Age 40-69 years<br>• Residence in survey area for ≥6 months<br>**Exclusion criteria:**<br>• Unknown glucose status<br>• Previous diabetes history<br>• Loss to follow-up, missing waist circumference data<br>• BMI <18.5 kg/m²<br>• Invalid/missing BIA data<br>**Setting and population:**<br>• 3252 men and 3643 women from Ansung and Ansan cohorts, Korea.	• MMI, calculated by dividing weight-adjusted ASM by weight in kg × 100 and categorized into sex-specific MMI tertiles: Lowest: <30.94%, middle: 30.94–33.11% and highest: ≥33.11% for men and <24.96%, 24.96–26.95% and ≥26.95% for women	1. Incident T2DM by self-report, use of oral hypoglycaemic agents and/or insulin, or OGTT results at baseline and for newly diagnosed T2DM during follow up, fasting glucose concentration of ≥7 mmol/l or a post-load glucose concentration of ≥11 mmol/l after a 75 g OGTT based on the WHO criteria	**Statistical analysis:**<br>• Cox Proportional Hazards Regression was used to calculate HRs for the risk of developing T2DM<br>• Multivariate-adjusted hazard ratios after adjusting for confounders.<br>• Adjusted for Body fat, BMI, WC and covariates<br>**Results/effects estimates:**<br>1. HRs for incident T2DM in each MMI tertile<br><u>Total participants</u><br>• Lowest MMI tertile: HR= 2.05 (95% CI: 1.73, 2.43)<br>• Middle MMI tertile: HR= 1.39 (95% CI: 1.17, 1.66)<br>• Highest MMI tertile: HR= 1.0 (ref.)<br>• Per 1 SD decline in MMI: HR= 1.35 (1.26, 1.45)<br><u>Excluding those who diagnosed within the first 2 years of follow-up</u><br>• Lowest MMI tertile: HR = 2.08 (95% CI: 1.72, 2.52)<br>• Middle MMI tertile: HR = 1.42 (95% CI:1.16, 1.73)<br>• Highest MMI tertile: HR = 1.00 (reference)<br>• Per 1 SD decline in MMI: HR = 1.36 (1.26, 1.47)	**Author's conclusions:**<br>The authors concluded that: "Low muscle mass as defined by MMI was associated with an increased risk of type 2 diabetes, independent of general obesity, in middle-aged and older Korean adults."
**Authors:**<br>Srikanthan et al.,	**Sample size:**<br>N= 13,644, mean ±SD BMI: 27.0±3.8 kg/m^2	• SMI, calculated as ratio of estimated	1. Insulin resistance, measured by HOMA-IR	**Statistical analysis:**<br>• Descriptive statistics, (%)	**Author's conclusions:**<br>The authors concluded that: "Across the full

**Year published:**<br>2011<br>**Study design:**<br>Cross-sectional study<br>**Country:**<br>United States	**Inclusion criteria:**<br>• Older than 20 years<br>• BMI of at least 16 kg/m² and body weight more than 35 kg; without self-reported cardiac failure.<br>**Exclusion criteria:**<br>• Previous limb amputation.<br>**Setting and population:**<br>• Participants from the NHANES III survey, United States	skeletal muscle mass to total body weight<br>• MMI, calculated as ratio of skeletal muscle to the square of body height in kg per square meter ?consider (kg/m^2)?	2. Dysglycemia, measured by HbA1C<br>3. DM prevalence<br>4. Prediabetes prevalence	• Multivariable linear and Poisson regression was used to examine the associations of SMI and MMI with continuous outcome variables.<br>• Adjusted for age, sex, race and WC<br>**Results/effects estimates:**<br>1. Prevalence of DM (%): 10.2%<br>2. Prevalence of PDM (%): 32.5%<br>3. Poisson regression analysis for SMI (by quartiles) with insulin resistance and dysglycemia<br>3.1. HOMA-IR ratio:<br>• 2nd quartile vs. ref. (Lowest quartile) = 0.95 (95% CI: 0.90, 1.00)<br>• 3rd quartile vs. ref. = 0.87 (95%CI: 0.83, 0.92)<br>• Highest quartile vs. ref. = 0.74 (95%CI: 0.69, 0.80)<br>3.2. HbA1C ratio:<br>• 2nd quartile vs. ref. = 1.004 (95%CI: 0.994, 1.015)<br>• 3rd quartile vs. ref. = 1.003 (95%CI: 0.989, 1.018)<br>• Highest quartile vs. ref. = 1.007 (95% CI: 0.989, 1.026)<br>3.3. PDM risk ratio:<br>• 2nd quartile vs. ref. = 0.86 (95%CI: 0.76, 0.96)<br>• 3rd quartile vs. ref. = 0.81 (95%CI: 0.70, 0.93)<br>• Highest quartile vs. ref. = 0.72 (95%CI: 0.60, 0.87)	range, higher muscle mass (relative to body size) is associated with better insulin sensitivity and lower risk of PDM.”

				3.4. Diabetes risk ratio:<br>• 2nd quartile vs. ref. = 1.33 (95% CI: 1.05, 1.69)<br>• 3rd quartile vs. ref. = 1.44 (95% CI: 1.01, 2.05)<br>• Highest quartile vs. ref. = 1.16 (95% CI: 0.79, 1.70)<br><u>After excluding diabetics</u><br>3.5. HOMA-IR ratio:<br>• 2nd quartile vs. ref. = 0.91 (95%CI: 0.88, 0.95)<br>• 3rd quartile vs. ref. = 0.83 (95%CI: 0.80, 0.87)<br>• Highest quartile vs. ref. 0.71 (95%CI: 0.67, 0.75)<br>3.6. HbA1C ratio:<br>• 2nd quartile vs. ref. = 0.987 (95%CI: 0.982, 0.993)<br>• 3rd quartile vs. ref. = 0.983 (95%CI: 0.975, 0.991)<br>• Highest quartile vs. ref. = 0.986 (95%CI: 0.976, 0.996)<br>3.7. Prediabetes risk ratio:<br>• 2nd quartile vs. ref. = 0.72 (95%CI: 0.63, 0.83)<br>• 3rd quartile vs. ref. = 0.65 (95%CI: 0.55, 0.76)<br>• Highest quartile vs. ref. = 0.59 (95%CI: 0.48–0.72)<br>4. Poisson regression for SMI and MMI with insulin resistance and dysglycemia:<br>4.1. SMI (per 10% increase):	

				• HOMA-IR ratio = 0.89 (95%CI: 0.85, 0.94)<br>• PDM risk ratio = 0.88 (95% CI: 0.79, 0.99)<br>After excluding diabetics:<br>• HOMA-IR ratio = 0.86 (95% CI: 0.82, 0.89)<br>• HbA1C ratio =1.001 (95% CI: 0.995, 1.008)<br>• Prediabetes risk ratio = 0.77 (95% CI: 0.67, 0.89)<br>4.2. MMI (per kg/m²):<br>• HOMA-IR ratio = 0.97 (95% CI: 0.96, 0.995)<br>• PDM risk ratio = 0.96 (95% CI: 0.92, 0.997)<br>After excluding diabetics:<br>• HOMA-IR ratio: = 0.96 (95% CI, 0.94, 0.97)<br>• HbA1C ratio = 0.997 (95% CI, 0.994, 0.9997)<br>• Prediabetes risk ratio = 0.91 (95% CI, 0.87, 0.96)	
**Authors:** Tyrovolas et al.,<br>**Year published:** 2019<br>**Study design:** Prospective cohort study<br>**Country**	**Sample size:**<br>N= 1019 subjects aged 45+ years, mean ±SD BMI: 31.0±4.5 kg/m^2<br>**Inclusion criteria:**<br>• Non-institutionalized adults from the Greek general population<br>• Aged 18 years or older	• Skeletal muscle mass, measured using a skeletal muscle mass index (SMI)<br>• ASM, calculated using a formula specific to the Caucasian population	1. CVD, defined based on the International Classification of Diseases, 10th version (ICD-10)<br>2. Hypercholesterolaemia<br>3. Diabetes	**Statistical analysis:**<br>• Cox proportional hazard regression was used to evaluate the 10-year CVD risk by baseline muscle mass tertiles<br>• Adjusted for age, sex, physical activity, diet adherence, hypertension, diabetes, and hypercholesterolemia<br>**Results/effects estimates:**	**Author's conclusions:**<br>The authors concluded that: "The presented findings support the importance of SMM evaluation in the prediction of long-term CVD risk among adults 45+ years old without pre-existing

Greece	• Without pre-existing CVD<br>**Exclusion criteria:**<br>• Not stated<br>**Setting and population:**<br>• Caucasian adults from Athens metropolitan region of Attica, Greece			1. Cox proportional hazard model for 10-year risk of developing CVD: HR = 0.06(95% CI: 0.005, 0.78)<br>2. Cox proportional hazards model by baseline muscle mass tertile<br>• 2nd tertile vs. ref. (1st tertile): HR = 0.46 (95% CI: 0.11, 1.81)<br>• 3rd tertile vs. ref.: HR = 0.19 (95% CI: 0.04, 0.85)	CVD. Preservation of SMM may contribute to CVD health."
**Authors:** Xiao et al., **Year published:** 2022 **Study design:** Cross-sectional study **Country:** United States	**Sample size:**<br>N= 2065, mean ± SD BMI: 28.2 ± 6.3<br>**Inclusion criteria:**<br>• Adults over 18 years with complete data for DXA vibration controlled transient elastography (VCTE), body measures, total cholesterol, and alanine aminotransferase<br>**Exclusion criteria:**<br>• Participates having missing or invalid data<br>• Participants with morbid obesity.<br>**Setting and population:**<br>• Adults US population	• Skeletal muscle mass, measured using DXA<br>• SMI, defined as skeletal muscle mass by height squared (kg/m^2)<br>• Appendicular SMI, defined as the sum of both arms and legs skeletal muscle mass divided by height squared (kg/m^2)<br>• Trunk SMI, defined as trunk skeletal muscle mass by height squared (kg/m^2)<br>• Adequate skeletal muscle mass status, defined as the SMI in the highest quartile	1. Metabolic dysfunction-associated fatty liver disease (MAFLD), defined as the presence of hepatic steatosis (CAP scores ≥ 263 dB/m) with at least one of the following: (1) overweight or obesity (body mass index (BMI) ≥ 25 kg/m2), (2) T2DM, or (3) at least 2 metabolic risk abnormalities<br>2. Significance fibrosis, defined as a median LSM ≥ 8 kPa	**Statistical analysis:**<br>• Survey-weight adjusted multivariable logistic regression was used to determine associations between the adequate muscle mass and MAFLD or significant fibrosis.<br>• Adjusted for demographic and lifestyle factors, adiposity measures (BMI, waist circumference, FMI), and biochemical markers (cholesterol, ALT, AST).<br>**Results/effects estimates:**<br>1. Logistic regression analysis for MAFLD:<br>1.1. Adequate SMI, yes vs. ref. (no):<br>• All: OR = 0.81 (95%CI: 0.44, 1.50)<br>• Male: OR = 0.70 (95%CI: 0.30, 1.60)<br>• Female: OR = 0.60 (95%CI: 0.27, 1.33)<br>1.2. Adequate appendicular SMI, yes vs. ref. (no):<br>• All: OR = 0.61 (95%CI: 0.36, 1.03)<br>• Male: OR = 0.46 (95%CI: 0.25, 0.84)<br>• Female: OR = 0.32 (95%CI: 0.13, 0.82)<br>1.3. Adequate trunk SMI, yes vs. ref. (no):<br>• All: OR = 0.85 (95%CI: 0.5, 1.43)<br>• Male: OR = 0.89 (95%CI: 0.41, 1.93)	**Author's conclusions:** The authors concluded that: "The distribution of skeletal muscle mass differently affects MAFLD and significant fibrosis in the sex groups. Higher appendicular skeletal muscle mass was associated with a lower risk of MAFLD, while the risk of significant fibrosis in females was increased with the trunk skeletal muscle mass."

				• Female: OR = 0.53 (95%CI: 0.24, 1.16)<br>2. Logistic regression for sensitivity analysis<br>2.1. Adequate SMI yes vs. ref.(no):<br>• All: OR = 0.98 (95%CI: 0.55, 1.75)<br>• Male: OR= 0.91 (95%CI: 0.44, 1.89)<br>• Female: OR= 0.63 (95% CI: 0.28, 1.42)<br>2.2. Adequate appendicular SMI, yes vs. ref.(no):<br>• All: OR = 0.66 (95% CI: 0.39, 1.10)<br>• Male: OR= 0.52 (95% CI: 0.29, 0.95)<br>• Female: OR = 0.29 (95% CI: 0.11, 0.75)<br>2.3. Adequate trunk SMI, yes vs. ref.(no):<br>• All: OR= 1.08 (95% CI: 0.70, 1.65)<br>• Male: OR = 0.90 (95% CI: 0.41, 1.96)<br>• Female: OR = 1.00 (0.51, 1.96)<br>3. Logistic regression for significant fibrosis<br>3.1. Adequate SMI, yes vs. ref.(no):<br>• All: OR= 1.22 (95%CI: 0.61, 2.47)<br>• Male: OR= 1.07 (95%CI: 0.41, 2.76)<br>• Female: OR= 8.68 (95%CI: 1.56, 48.12)<br>3.2. Adequate appendicular SMI, yes vs. ref.(no):<br>• All: OR = 0.39 (95%CI: 0.14, 1.08)<br>• Male: OR= 0.59 (95%CI: 0.21, 1.61)<br>• Female: OR= 1.67 (0.38, 7.26)<br>3.3. Adequate trunk SMI, yes vs. ref.(no):<br>• All: OR= 1.48 (95%CI: 0.71, 3.09)<br>• Male: OR = 1.15 (95% CI: 0.54, 2.45)<br>• Female: OR = 7.82 (95%CI: 1.86, 32.77)	

**Authors:** Yanek et al., **Year published:** 2015 **Study design:** Cross-sectional study **Country:** United States	**Sample size:** N=191, mean ± SD BMI: 34 ± 5 kg/m^2) **Inclusion criteria:** • African American with overweight or obesity (BMI ≥25) participants **Exclusion criteria:** • Pregnant women at the time of screening **Setting and population:** • Healthy, overweight, sedentary African Americans from families with a history of early-onset coronary disease in Baltimore, Maryland	• Lean mass, measured using DEXA	1. Cardiorespiratory fitness, measured by VO_2peak (L/min)	**Statistical analysis:** • Multiple linear regression was used to assess prediction of physical fitness. • Adjusted for age, smoking, physical activity, BMI category, and fat mass. **Results/effects estimates:** 1. Multiple linear regression analysis: • (Log) lean mass (kg) male: $\beta = 0.5219$, $P = 0.0358$ • (Log) lean mass (kg) female: $\beta = 0.5312$, $p<.0001$	**Author's conclusions:** The authors concluded that: "In this cross-sectional study, lean mass was the key determinant of cardiorespiratory fitness, independent of sex, age, and magnitude of obesity."
**Authors:** Yoo et al., **Year published:** 2020 **Study design:** Cross-sectional study **Country:** Republic of Korea	**Sample size:** N= 31,258 mean ± SD BMI: 26.3 ± 2.9 for lowest SMI tertiles (n = 10 419) **Inclusion criteria:** • Subjects aged 20 years or older • Individuals who underwent comprehensive health examinations including echocardiography at Samsung Medical Centre between	1. SMI, calculated by dividing the sum of ASM values in the bilateral upper and lower four limbs (kg) by body weight (kg) and was expressed as a percentage (= total ASM/body weight × 100%).	1. Left ventricular diastolic dysfunction, defined as an EF of greater than 50% and (a) E/e0 ratio greater than 15 or (b) E/e0 ratio of 8 to 15 and LAVI greater than or equal to 34 mL/m	**Statistical analysis:** • Multiple logistic regression was used to evaluate the association between sex-specific SMI tertiles and left ventricular diastolic dysfunction (LVDD). • Adjusted for age, sex, smoking, alcohol, exercise, LAVI, WC, diabetes, hypertension, dyslipidemia, and CKD **Results/effects estimates:** 1. OR for lowest SMI tertile vs. highest(ref.): 1.56 (95% CI: 1.31-1.85)	**Author's conclusions:** The authors concluded that: "Our findings demonstrate that lower skeletal muscle mass and sarcopenic obesity are strongly associated with diastolic dysfunction in middle-aged and older adults."

	September 2006 and December 2013.<br>**Exclusion criteria:**<br>• Subjects with missing or erroneous echocardiography data<br>• Individuals with missing skeletal muscle index (SMI) data<br>• Participants with an estimated glomerular filtration rate (eGFR) less than 30 mL/min/1.73 m²<br>• Those with a left ventricular ejection fraction (LVEF) less than 50%<br>• Subjects lacking baseline waist circumference (WC) or total cholesterol data<br>**Setting and population:**<br>Korean adults from Samsung Medical Centre's Health Promotion Centre, Seoul, Republic of Korea.				
**Authors:**<br>Atlantis et al.,<br>**Year published:**<br>2009	**Sample size:**<br>N= 1,195, mean ±SD BMI: 25.0±3.5 kg/m^2<br>**Inclusion criteria:**	2. Whole-body Lean Mass, measured as a percentage of total body mass using DEXA	1. Prevalence of MetS, as defined by both the Adult Treatment Panel III (ATPIII) and International Diabetes	**Statistical analysis:**<br>• Multiple-adjusted binary logistic regression models with a stepwise selection process were used to identify and quantify the association between whole-	**Author's conclusions:**<br>The authors concluded that: "Our findings demonstrate that lower

**Study design:** Cross-sectional study<br>**Country:** Australia	• Men aged 35 to 80 years living in the northwest regions of Adelaide, Australia, who attended clinical assessments as part of the Florey Adelaide Male Ageing Study (FAMAS).<br>**Exclusion criteria:**<br>• Not stated<br>**Setting and population:**<br>• Northwestern regions of Adelaide, Australia, comprising men aged 35 to 81 years from the Florey Adelaide Male Ageing Study, conducted between 2002 and 2005	3. Quartiles, defined as<br>• Q1: (51.6-65.1)<br>• Q2: (65.2-68.9)<br>• Q3: (69.0-73.3)<br>• Q4: (73.4+)	Federation (IDF) criteria.	body lean mass and the presence of MetS, as defined by the ATPIII and IDF criteria<br>• Adjusted for demographic, physical, and lifestyle factors, abdominal fat mass, insulin, muscle strength, and medical conditions<br>**Results/effects estimates:**<br>1. Odds Ratios of Whole-Body Lean Mass for present ATPIII MetS and PAR%<br>• Q1: OR = 3.34 (95%CI: 1.63, 6.84), PAR% = 27%<br>• Q2: OR = 2.51 (95%CI: 1.25, 5.04), PAR% = 18%<br>• Q3: OR= 1.61 (95%CI: 0.79, 3.28), PAR% = 8%<br>• Q4: Reference (1.0)<br>2. Odds ratios of Whole-Body Lean Mass for present IDF MetS and PAR%<br>• Q1: OR = 4.85 (95%CI: 2.23, 10.54), PAR% = 31%<br>• Q2: OR = 5.45 (95%CI: 2.56, 11.6), PAR% = 24%<br>• Q3: OR= 2.96 (1.38, 6.36), PAR% = 14%<br>• Q4: Reference (1.0)	skeletal muscle mass and sarcopenic obesity are strongly associated with diastolic dysfunction in middle-aged and older adults."
**Authors:** Li et al.,<br>**Year published:** 2016<br>**Study design:** Cohort study<br>**Country:**	**Sample size:**<br>N= 118, mean ± SD BMI: 28.0 ± 4.2 kg/m²<br>**Inclusion criteria:**<br>• nclusion criteria:<br>• Community-dwelling men aged ≥35 years.	4. Lean mass percentage (per 5% increase, DXA-based)	2. Incident T2D	**Statistical analysis:**<br>Multiple-adjusted binary logistic regression models were used to assess associations between lean mass percentage (per 5% increase, DXA-based) and incident type 2 diabetes<br>Adjusted for age, income, cohort, waist circumference, fasting plasma glucose,	**Author's conclusions:**<br>The authors concluded that: "Reduced muscle strength, but not reduced muscle mass, is a risk factor for incident T2DM in men. This is not

Australia	• Participants of the MAILES cohort, which combined the Florey Adelaide Male Ageing Study (FAMAS) and the Northwest Adelaide Health Study (NWAHS)<br>• **Exclusion criteria:**<br>• Men with diabetes at baseline (n = 379).<br>• Men with major health issues or on testosterone-affecting medications.<br>• **Setting and population:**<br>Northwestern regions of Adelaide, Australia, comprising men aged 35 to 81 years from the Florey Adelaide Male Ageing Study, conducted between 2002 and 2005			physical activity, hypertension, triglycerides and family history of diabetes<br>**Results/effects estimates:**<br>OR with 95% CI: 0.96 (0.80, 1.20**)**	mediated by testosterone or inflammation. Intervention could prevent a substantial proportion of disease."

Abbreviations: LMB: Lean Body Mass; FFM: Fat-Free Mass; FFMI: Fat-free mass Index; FM: Fat Mass; BIA: Bioelectrical Impedance Analysis; BMI: Body Mass Index; DXA: Dual Energy X-ray Absorptiometry; QUICKI: Quantitative Insulin Sensitivity Check Index; ROC: Receiver Operating Characteristic; ASM: Appendicular Skeletal Muscle Mass; AUC: Area Under Curve; CRF: Cardiorespiratory fitness; SMI: Skeletal Muscle Mass Index; VO2peak: Maximal Oxygen Uptake; METpeak: peak metabolic equivalents; ANOVA: Analysis Of Variance; OR: Odds Ratio; CI: Confidence Interval; CT: Computed Tomography; NAFLD: Non-Alcoholic Fatty Liver Disease; MetS: Metabolic Syndrome; HR: Hazard Ratio; PNPLA3: Patatin-like Phospholipase Domain-Containing Protein 3; KSOS: Sarcopenic Obesity Study; CKD: Chronic Kidney Disease; eGFR: Estimated Glomerular Filtration Rate; ACR: Albumin to Creatinine Ratio; PCOS: polycystic ovary syndrome; HOMA: Homeostatic, Model Assessment;

OGTT: Oral Glucose Tolerance Test; CCRC: Clinical Research Center; IR: Insulin Resistance; CRP: C-Reactive Protein; LM: Lean Mass; LSMM: Low Skeletal Muscle Mass; IRR: Incidence Rate Ratios; FS-IVGTT: Frequently Sampled Intravenous Glucose Tolerance Test; CVD: Cardiovascular Disease; NCEP ATP III: National Cholesterol Education Program Adult Treatment Panel III; IDF: International Diabetes Federation; SMM: Skeletal Muscle Mass; ALM: appendicular lean mass; T2DM: Type 2 Diabetes Mellitus; HOMA-IR: Homeostatic, Model Assessment-Insulin Resistance; COPD: Chronic Obstructive Pulmonary Disease; 6MWD: Six-Minute Walking Distance; GOLD: Global Initiative for Chronic Obstructive Lung Disease; HADS: Hospital Anxiety and Depression Scale; AIX: Augmentation Index (AIx): DOQI: Dialysis Outcomes Quality Initiative; LBM: Lean Body Mass; LBMI: Lean Body Mass Index: hs-CRP: High-Sensitivity C-reactive Protein; ref.: Reference; PDM: Prediabetes Mellitus; MMI: Muscle Mass Index; VCTE: vibration controlled transient elastography; LVDD: Left Ventricular Diastolic Dysfunction; ASMI: Appendicular Skeletal Muscle Mass Index; Aix: augmentation index; cfPWV: Carotid-Femoral Pulse Wave Velocity; LEAD: Lower Extremity Artery Disease; WL: Weight Loss; SM: Skeletal Muscle; LSG: Laparoscopic Sleeve Gastrectomy; DTPA: Diethylenetetramine-Pentaacetic Acid; SCr: Serum Creatinine; CAVI: Cardio-Ankle Vascular Index; MAO: Metabolically Abnormal Obesity; NHANES: National Health and Nutrition Examination Survey; CER: Creatinine Excretion Rate; MF-BIA: Multifrequency Bioelectrical Impedance Analysis; MAFLD: Metabolic Dysfunction-Associated Fatty liver Disease; US: United States

Supplementary Table S4: Risk of bias assessment of studies reviewed

**Cohort studies**	**1. Were the two groups similar and recruited from the same population?**	**2. Were the exposures measured similarly to assign**	**3. Was the exposure measured in a valid and**	**4.Were confounding fact**	**5.Were strategies to deal**	**6. Were the groups/participants free of the outcome**	**7. Were the outcomes measured in a**	**8. Was the follow up time reported and sufficient to be long**	**9. Was follow up complete, and if not, were the reasons to**	**10. Were strategies to address incomplete follow**	**11. Was appropriate statistical**

		people to both exposed and unexposed groups?	reliable way?	ors identified?	with confounding factors stated?	at the start of the study (or at the moment of exposure)?	valid and reliable way?	enough for outcomes to occur?	loss to follow up described and explored?	up utilized?	analysis used?
Choe et al., 2023	Yes	Yes	Yes	Yes	Yes	Yes	Yes	Yes	No	No	Yes
Fenger et al., 2017	Yes	Yes	Yes	Yes	Yes	Yes	Yes	Yes	No	No	Yes
Itterman et al., 2019	Yes	Yes	Yes	Yes	Yes	Yes	Yes	Yes	No	Yes	Yes
Larsen et al., 2016	Yes	Yes	Yes	Yes	Yes	Yes	Yes	Yes	No	No	Yes
LeCroy et al., 2020	Yes	Yes	Yes	Yes	Yes	Yes	Yes	Yes	Yes	Yes	Yes
Lee et al., 2018	Yes	Yes	Yes	Yes	Yes	Yes	Yes	Yes	No	No	Yes
Nguyen et al., 2022	Yes	Yes	Yes	Yes	Yes	Yes	Yes	Yes	No	No	Yes
Sizoo et al., 2022	Yes	Yes	Yes	Yes	Yes	Yes	No	Yes	No	No	Yes
Son et al., 2017	Yes	Yes	Yes	Yes	Yes	Yes	Yes	Yes	No	No	Yes
Tyrovolas et al., 2019	Yes	Yes	Yes	Yes	Yes	Yes	Yes	Yes	Yes	Yes	Yes
Li et al., 2016	Yes	Yes	Yes	Yes	Yes	Yes	Yes	Yes	Yes	Yes	Yes

**Cross-Sectional Studies**	**1. Were the criteria for inclusion in the sample clearly defined?**	**2. Were the study subjects and the setting described in detail?**	**3. Was the exposure measured in a valid and reliable way?**	**4. Were objective, standard criteria used for measurement of the condition?**	**5. Were confounding factors identified?**	**6. Were strategies to deal with confounding factors stated?**	**7. Were the outcomes measured in a valid and reliable way?**	**8. Was appropriate statistical analysis used?**
Barsalani et al., 2013	Yes	Yes	Yes	Yes	No	No	Yes	Yes
Boo, S. H et al., 2018	Yes	Yes	Yes	Yes	Yes	Yes	Yes	Yes
Carvalho et al., 2020	Yes	Yes	Yes	Yes	Yes	Yes	Yes	Yes
Choe et al., 2018	Ye	Yes	Yes	Yes	Yes	Yes	Yes	Yes
Kevin et al., 2012	Yes	Yes	Yes	Yes	Yes	Yes	Yes	Yes
Fornari et al., 2015	Yes	Yes	Yes	Yes	No	No	Yes	Yes
Kang et al., 2021	Yes	Yes	Yes	Yes	Yes	Yes	Yes	Yes
Koo et al., 2015	Yes	Yes	Yes	Yes	Yes	Yes	Yes	Yes
Machado et al., 2021	Yes	Yes	Yes	Yes	Yes	No	Yes	Yes
Moreno et al., 2015	Yes	Yes	Yes	Yes	Yes	Yes	Yes	Yes
Muster et al., 2021	Yes	Yes	Yes	Yes	No	No	Yes	Yes

Ozmen et al., 2009	Yes	Yes	Yes	Yes	Yes	Yes	Yes	Yes
Park et al., 2022	Yes	Yes	Yes	Yes	Yes	Yes	Yes	Yes
Peppa et al., 2014	Yes	Yes	Yes	Yes	Yes	Yes	Yes	Yes
Pramyothin et al., 2017	Yes	Yes	Yes	Yes	Yes	Yes	Yes	Yes
Shao et al., 2023	Yes	Yes	Yes	Yes	Yes	Yes	Yes	Yes
Srikanthan et al., 2011	Yes	Yes	Yes	Yes	Yes	Yes	Yes	Yes
Xiao et al., 2022	Yes	Yes	Yes	Yes	Yes	Yes	Yes	Yes
Yanek et al., 2015	Yes	Yes	Yes	Yes	Yes	Yes	Yes	Yes
Yoo et al., 2020	Yes	Yes	Yes	Yes	Yes	Yes	Yes	Yes
Atlantis et al., 2009	Yes	Yes	Yes	Yes	Yes	Yes	Yes	Yes

Supplementary Table S5. Stratified sensitivity analyses according to FFM metric type across major weight-related metabolic outcomes.

**Study**	**FFM Metric (How reported)**	**Type**	**Outcome**	**Subgroup Pooled RR/OR (95% CI)**	**I^2**
Sizoo et al., 2023	CER (24-hour urinary creatinine)	Type A	T2DM		
Larsen et al., 2015	Total lean mass (kg)	Type A	T2DM		
Son et al., 2017	MMI (ASM/weight × 100)	Type B	T2DM		
Srikanthan et al., 2011	SMI (SMM/weight)	Type B	T2DM		
LeCroy et al., 2020	FFM percentage (%)	Type B	T2DM		

T2DM pooled estimates		Type A (n = 2)		1.32 (1.14–1.54)	0.0%
		Type B (n = 3)		1.56 (0.99–2.44)	95.3%
Carvalho et al., 2020	SMI/BMI	Type B	MetS		
Koo et al., 2015	ASM/weight	Type B	MetS		
Atlantis et al., 2009	Lean mass percentage (%)	Type B	MetS		
MetS pooled estimates		Type B (n = 3)		4.48 (2.18–9.22)	79.8%
Kang et al., 2021	ASM/BMI	Type B	CVD		
Yoo et al., 2020	SMI (ASM/weight × 100)	Type B	CVD		
Tyrovolas et al., 2019	SMI (ASM/BMI)	Type B	CVD		
CVD pooled estimates		Type B (n = 3)		2.03 (1.27–3.26)	36.1%
Xiao et al., 2022	SMI (SMM/height²)	Type A	NAFLD		
Choe et al., 2018	SMI-CT	Type B	NAFLD		
Choe et al., 2023	BMI-adjusted muscle mass	Type B	NAFLD		
NAFLD pooled estimates		Type A (n = 1)		Not pooled (N/A)	N/A
		Type B (n = 2)		2.51(1.61–3.91)	73.7%

Type A studies included absolute or height-adjusted FFM-related metrics, whereas Type B studies included relative or adiposity-adjusted FFM-related metrics. Pooled estimates represent subgroup-specific random-effects meta-analyses according to FFM metric type.

Abbreviations: *FFM, fat-free mass; CER, creatinine excretion rate; ASM, appendicular skeletal muscle mass; SMM, skeletal muscle mass; SMI, skeletal muscle index; MMI, muscle mass index; BMI, body mass index; CT, computed tomography; T2DM, type 2 diabetes mellitus; MetS, metabolic syndrome; CVD, cardiovascular disease; NAFLD, non-alcoholic fatty liver disease; RR, risk ratio; OR, odds ratio; CI, confidence interval; I^2, Higgins' inconsistency statistic.*

Forest plots of sensitivity and subgroup analyses for the association between low FFM metrics and T2DM

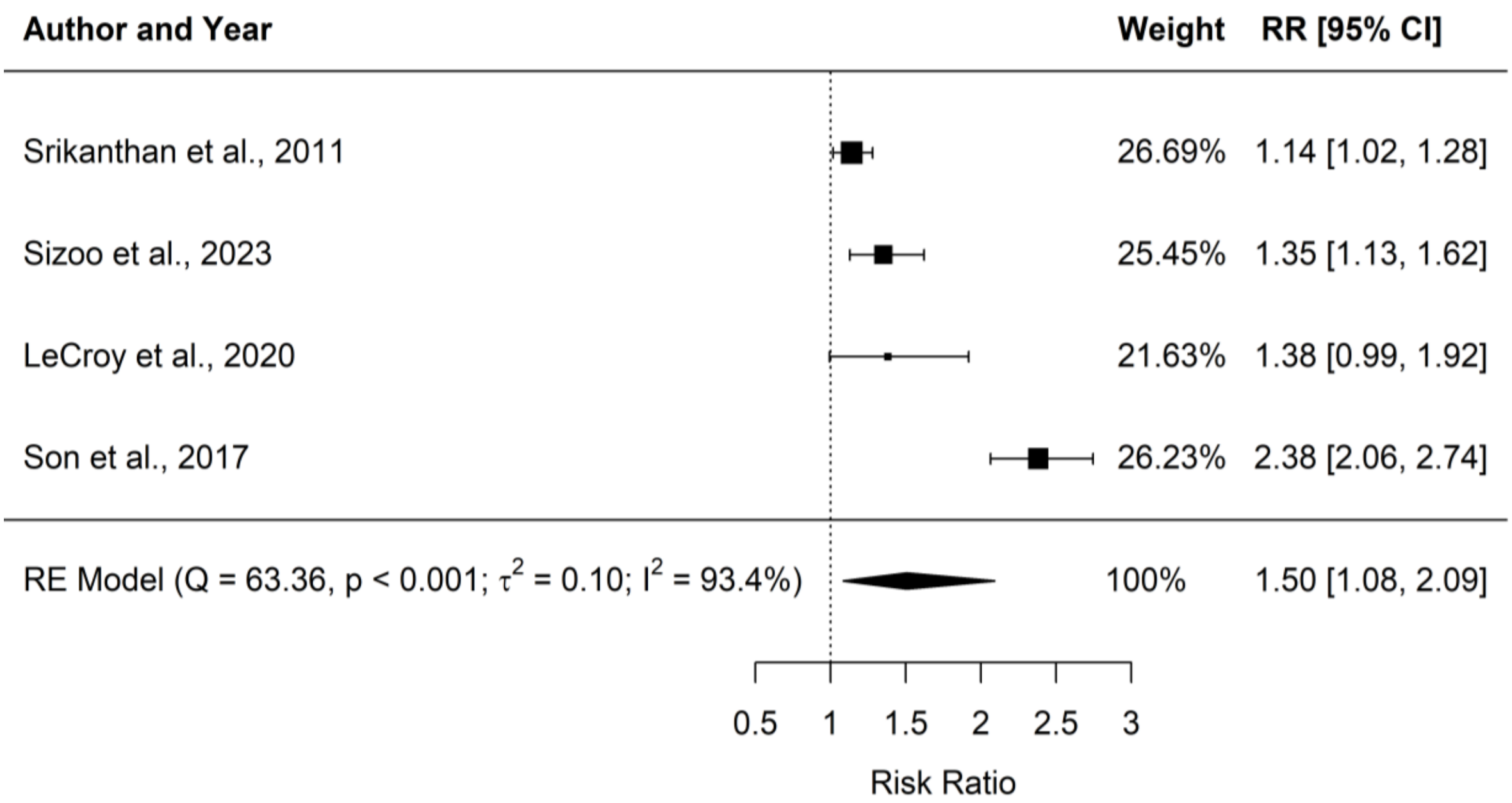

Supplementary Figure 1. Forest plot of sensitivity analysis for the association between low FFM and T2DM risk, excluding the older-age study (57).

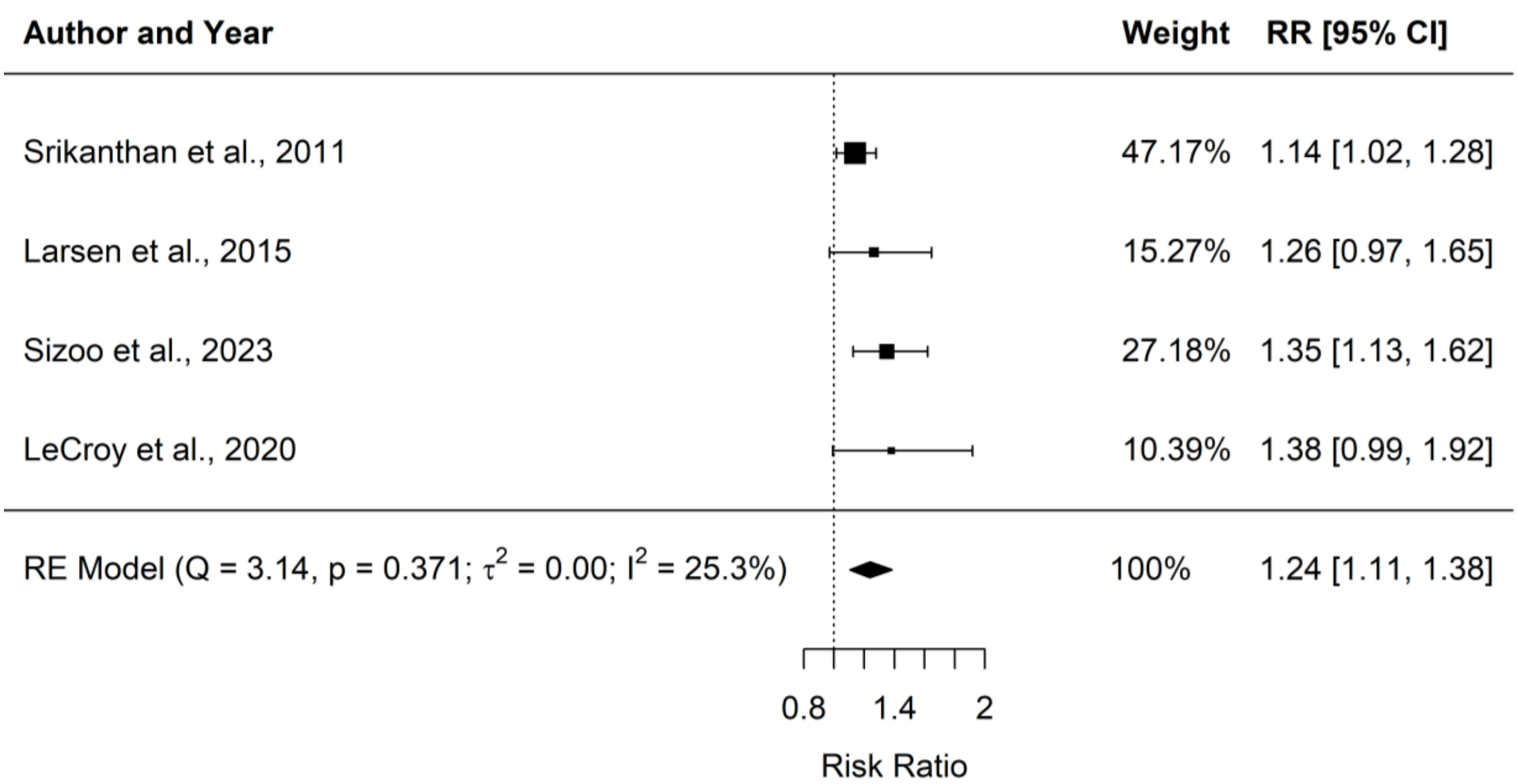

Supplementary Figure 2. Forest plot of sensitivity analysis for the association between low FFM and T2DM risk, excluding the study (19).

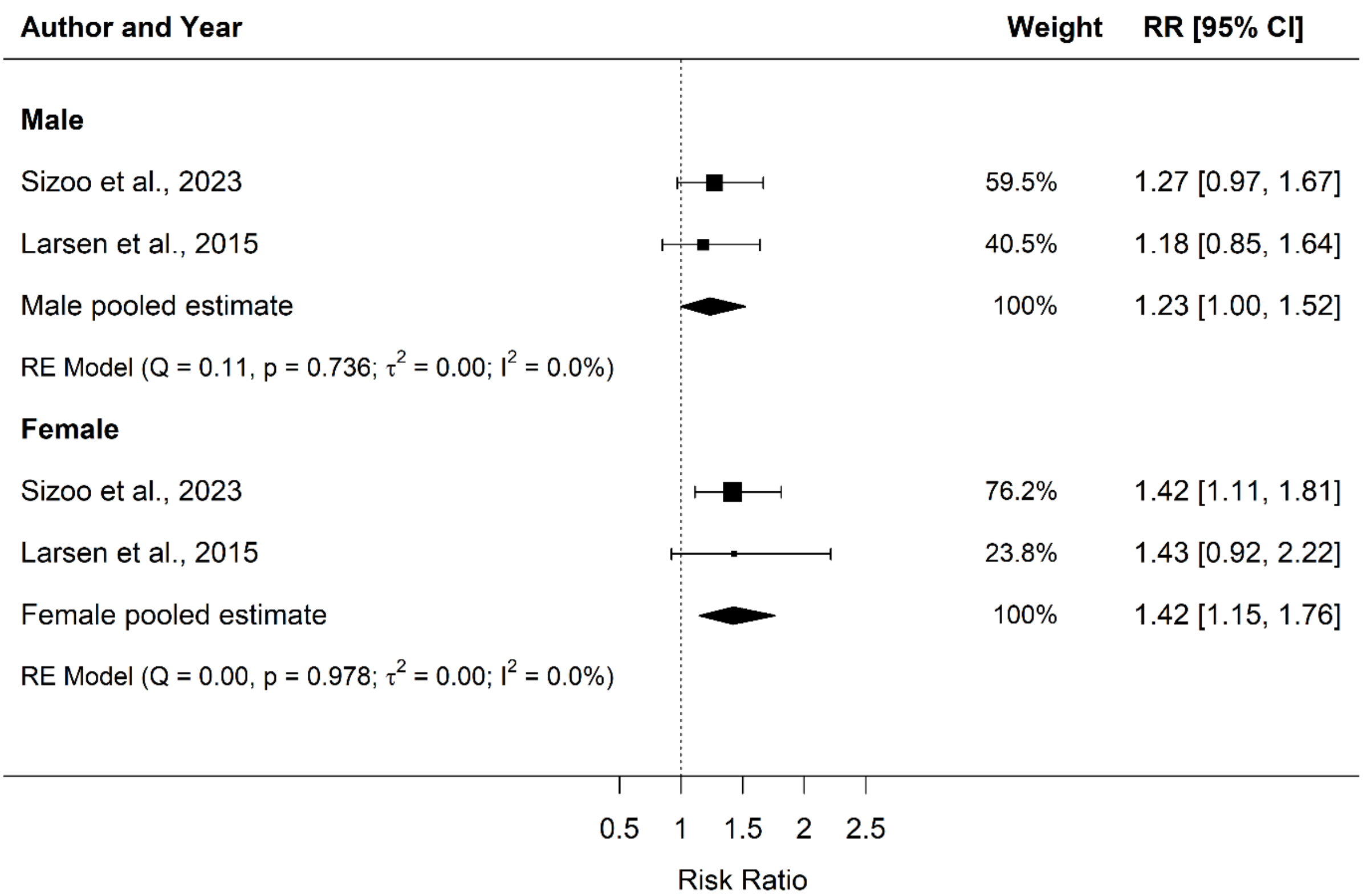

Supplementary Figure 3. Forest plot of association between low FFM and risk of T2DM incidence, stratified by sex.

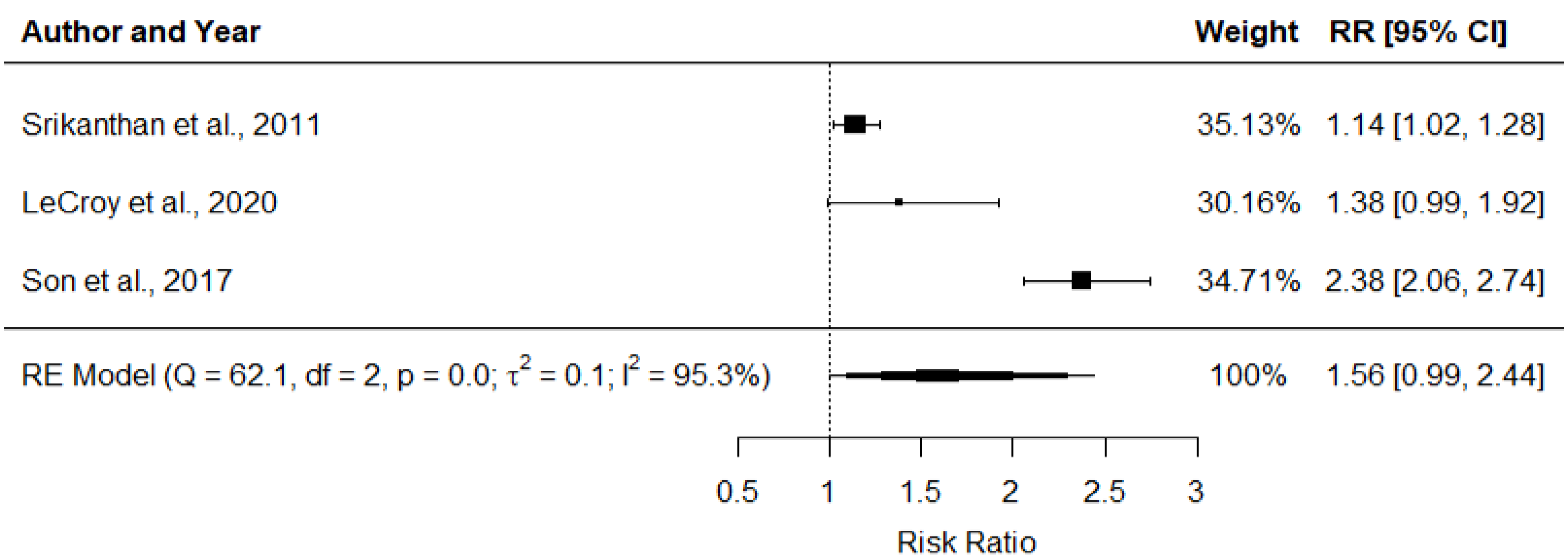

Supplementary Figure 4. Forest plot of the association between low FFM and T2DM risk for studies using BIA.

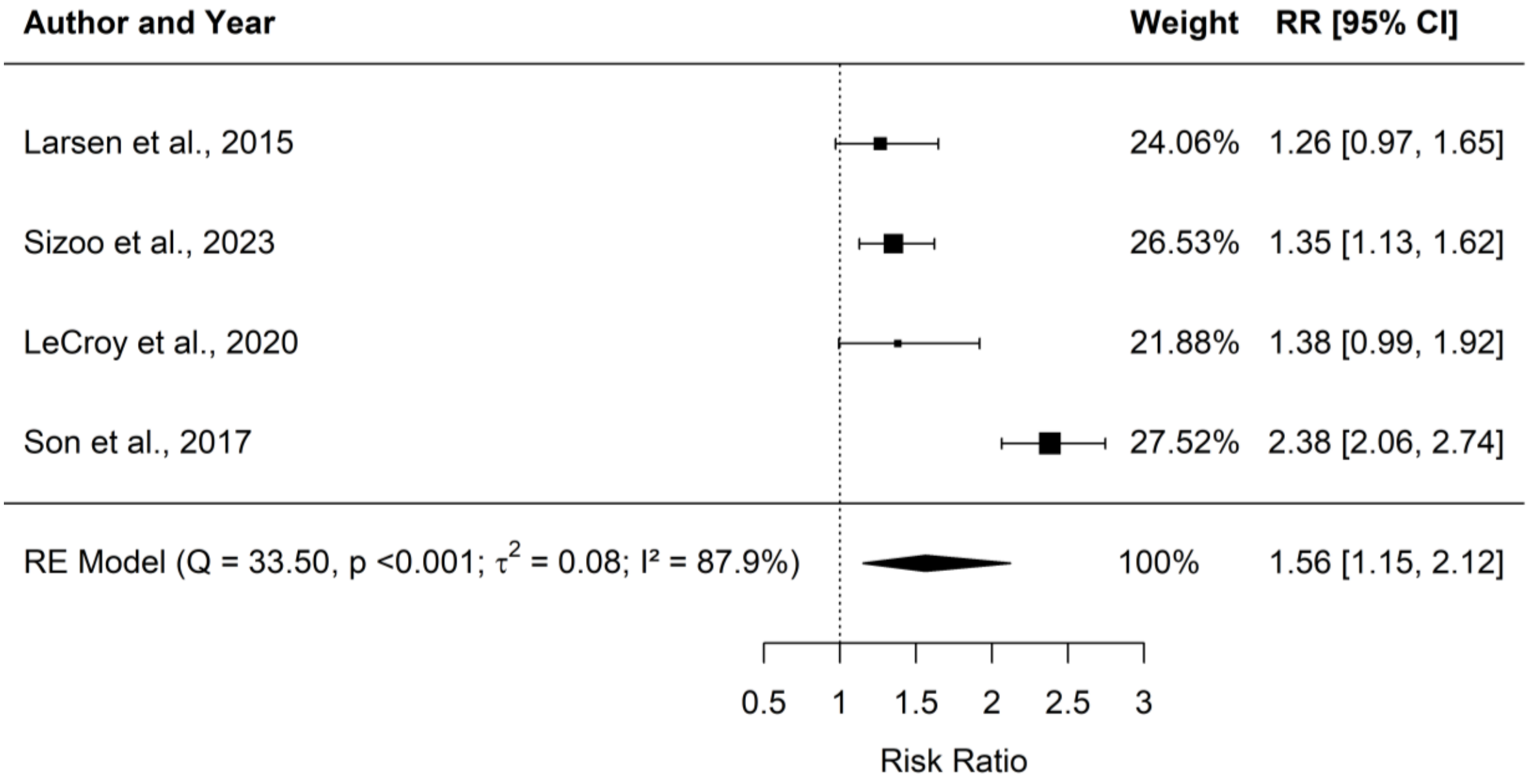

Supplementary Figure 5. Forest plot of association between low FFM and risk of T2DM incidence restricting on cohort studies.

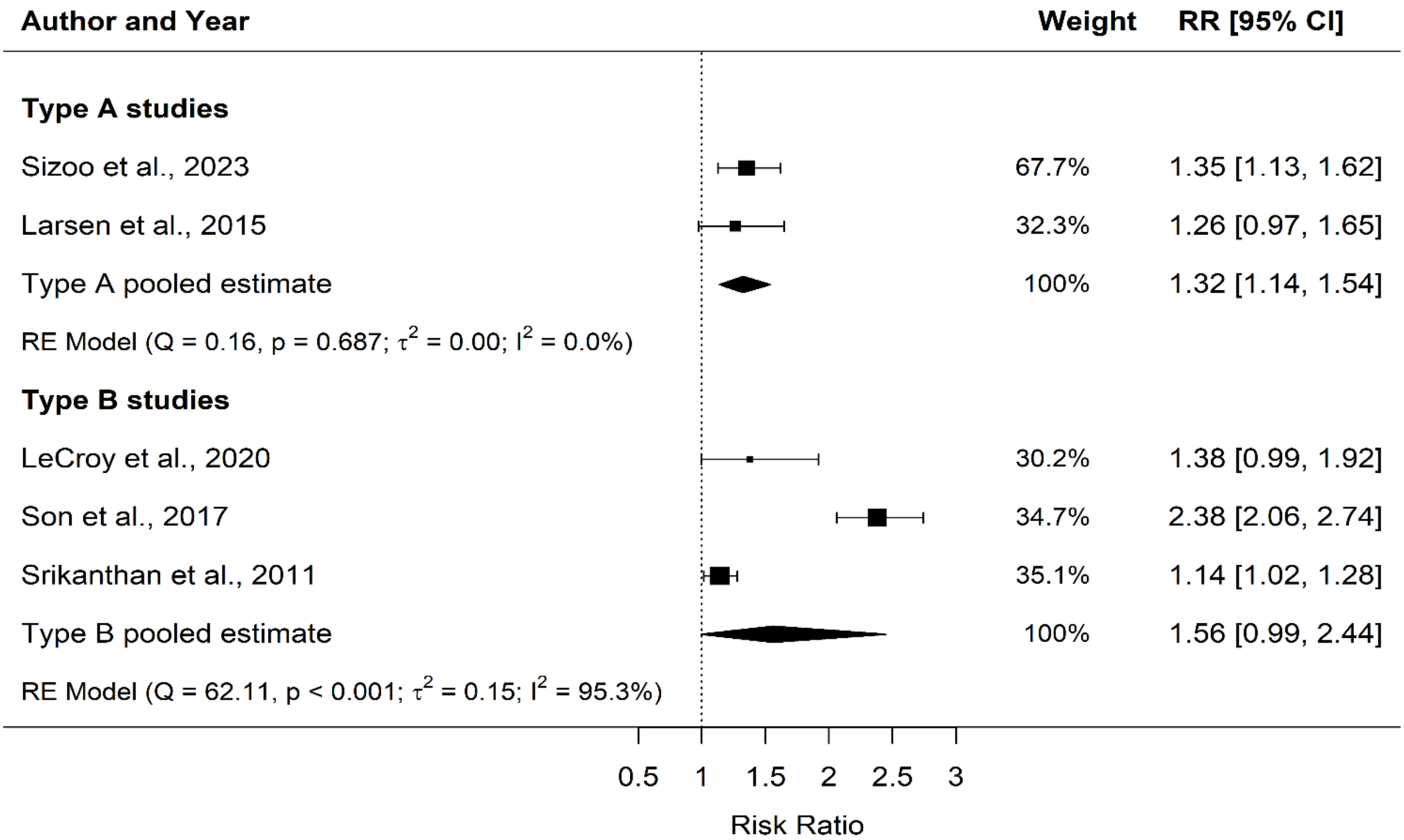

Supplementary Figure 6. Forest plot of association between low FFM and risk of T2DM incidence, stratified by FFM metrics types.

Forest plots of sensitivity and subgroup analyses for the association between low FFM and MetS.

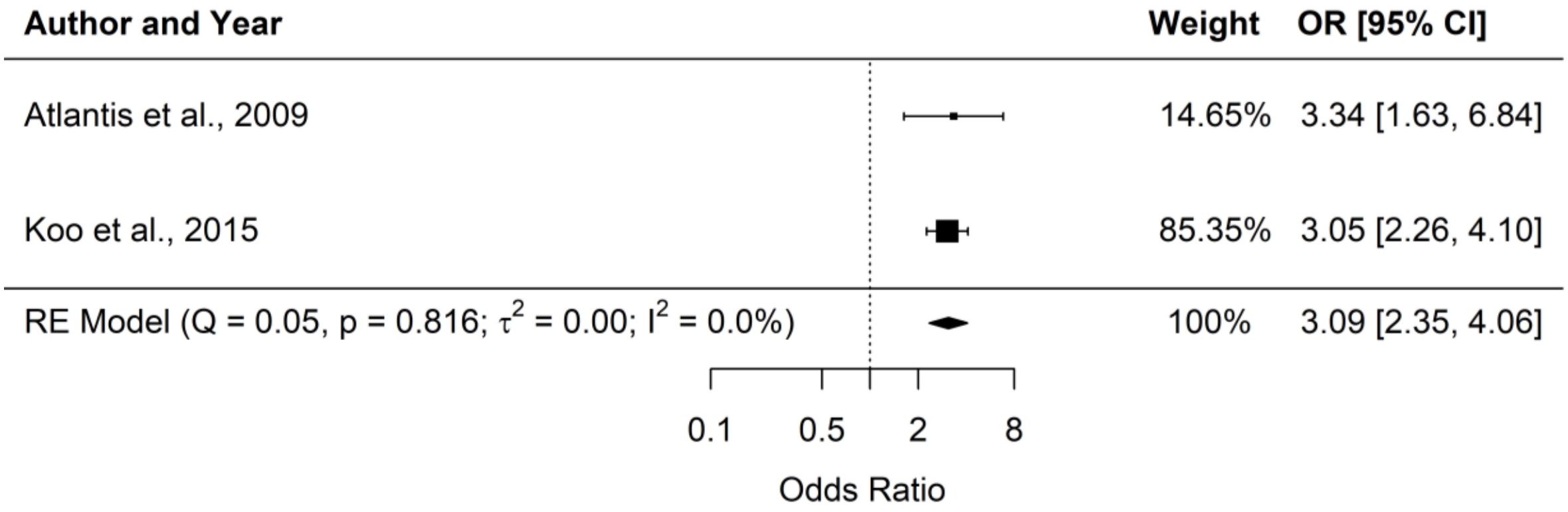

Supplementary Figure 7. Forest plot of sensitivity analysis for the association between low FFM and MetS excluding the study (36).

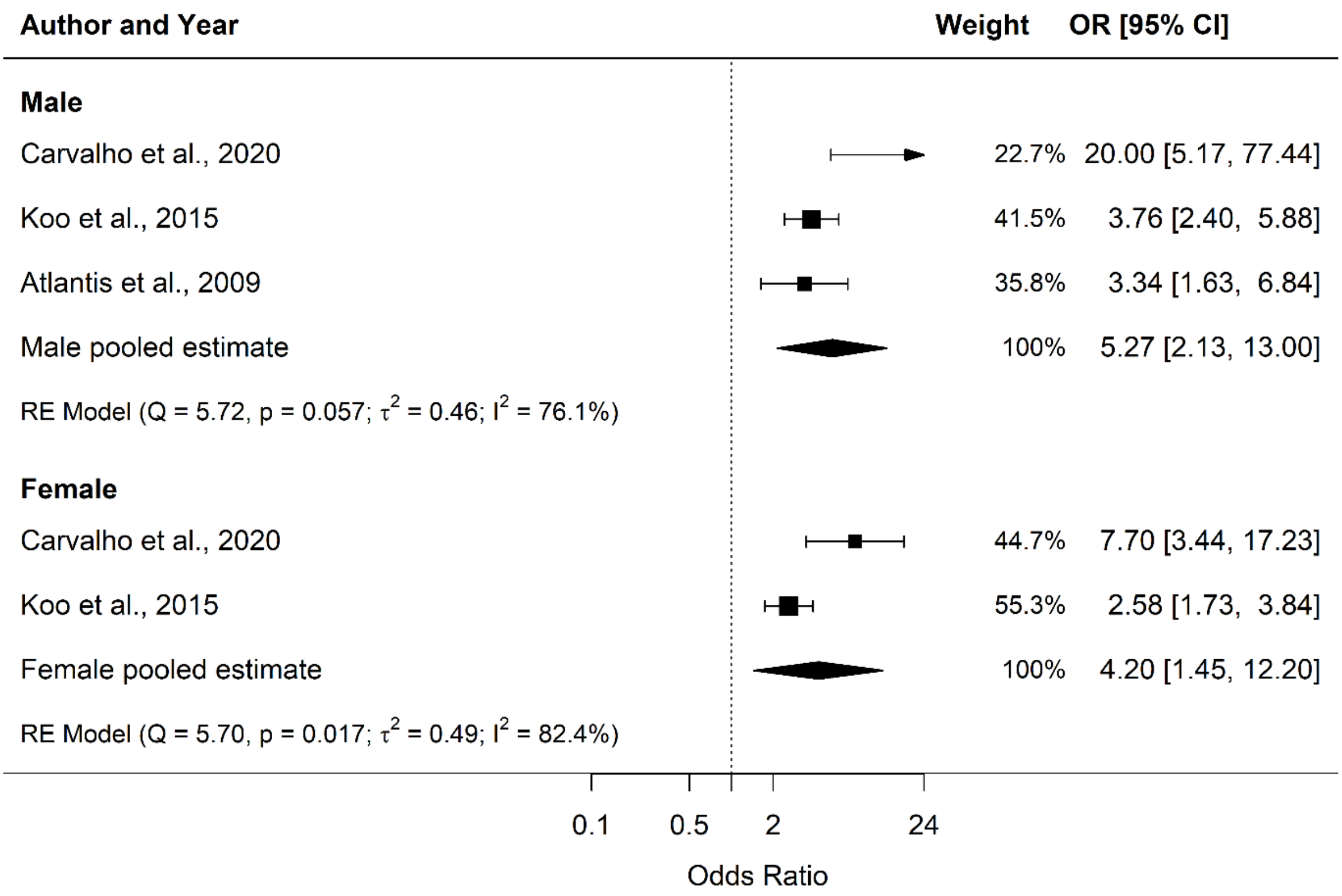

Supplementary Figure 8. Forest plot of association between low FFM and risk of MetS, stratified by sex.

Forest plot of sensitivity analysis for the association between low FFM and CVD risk

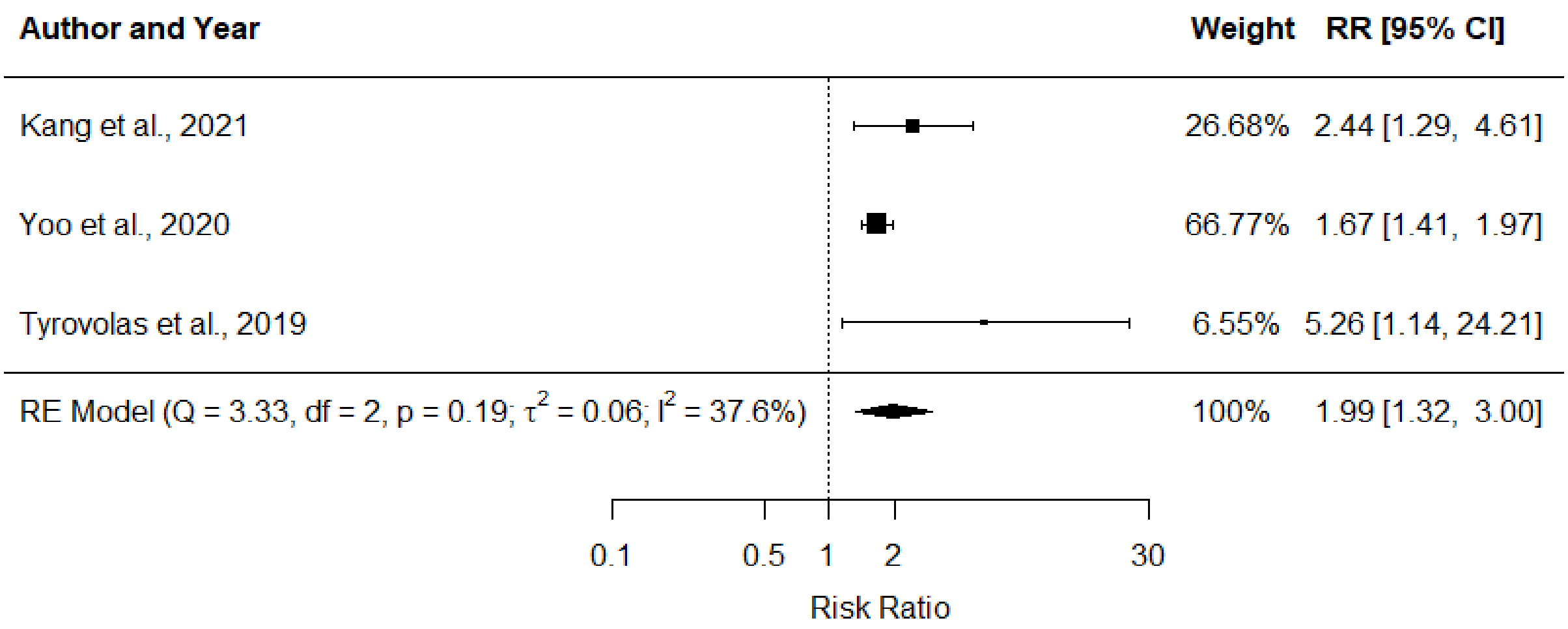

Supplementary Figure 9. Forest plot of sensitivity analysis for the association between low FFM and risk of CVD incidence, substituting the outcome measure in study (40).

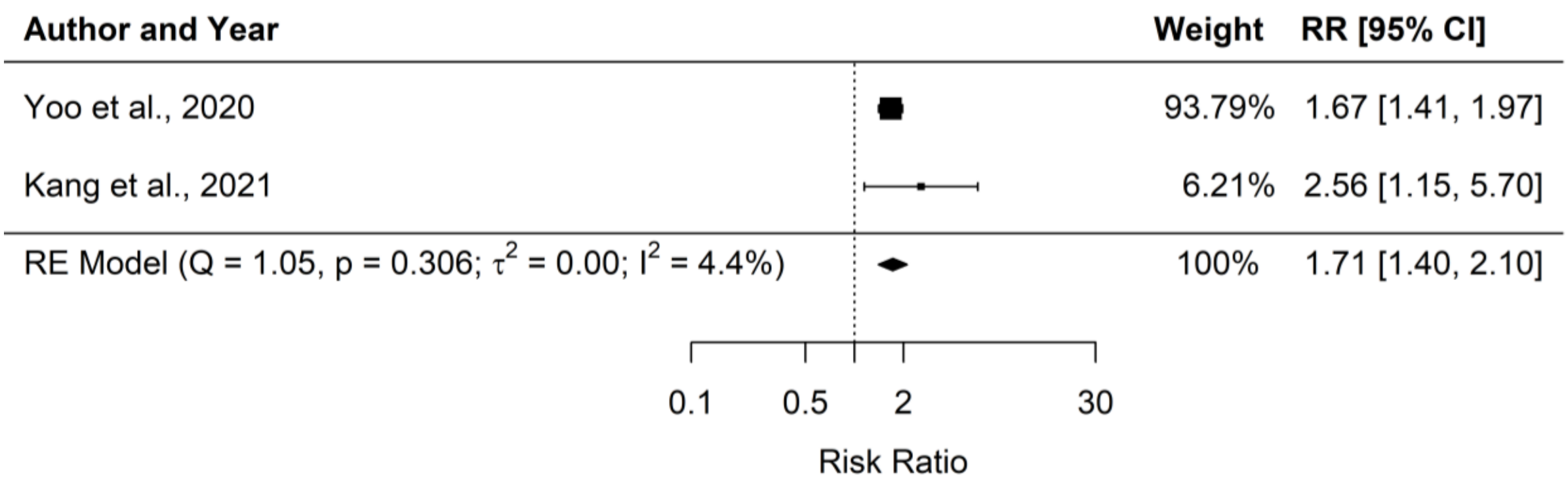

Supplementary Figure 10. Forest plot of sensitivity analysis for the association between low FFM and risk of CVD incidence, excluding the study (62).

Forest plots of sensitivity analyses for the association between low FFM and NAFLD

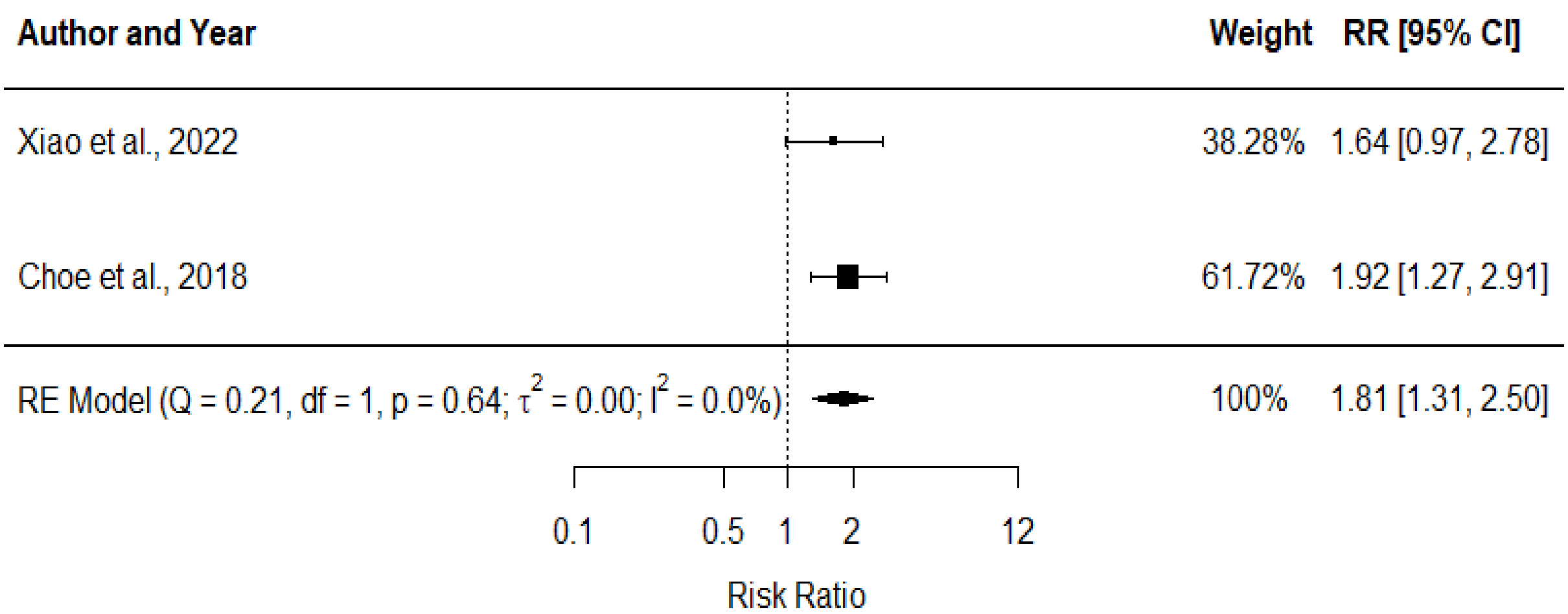

Supplementary Figure 11: Forest plot of sensitivity analysis for the association between low FFM and NAFLD risk, excluding one with the highest effect estimate (54).

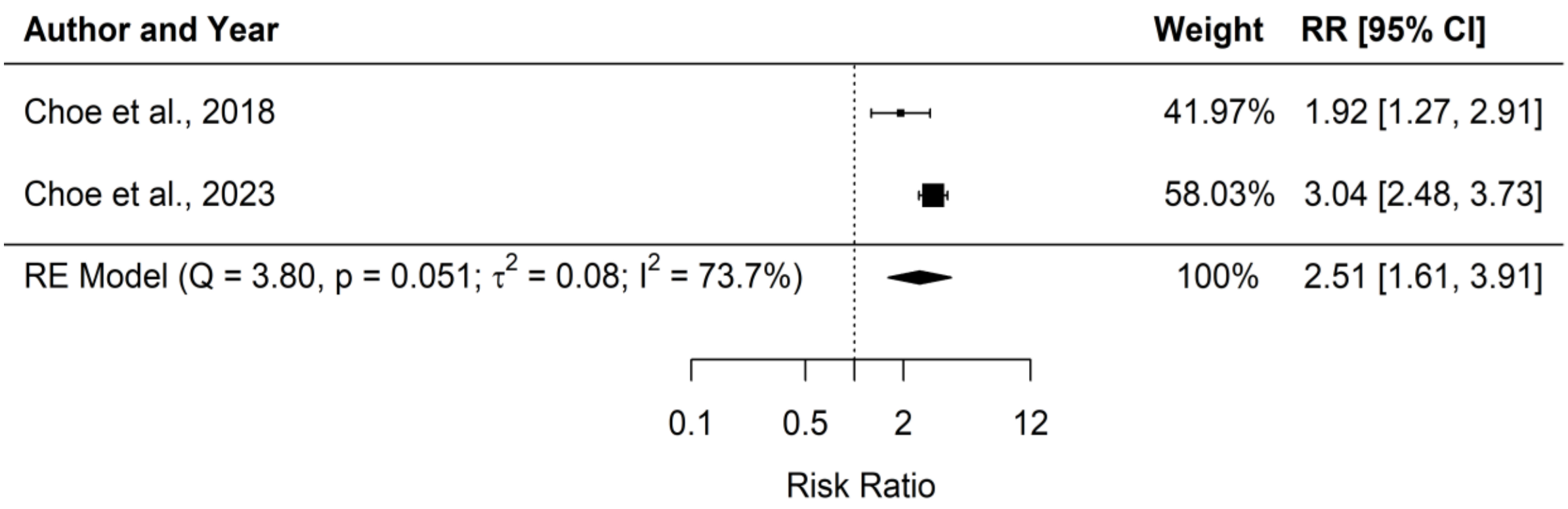

Supplementary Figure 12. Forest plot of sensitivity analysis for the association between low FFM and risk of NAFLD, excluding one type A study (absolute FFM measure) (40).

References for excluded studies

1. Prasad, V.K., et al., *Relation of Body's Lean Mass, Fat Mass, and Body Mass Index With Submaximal Systolic Blood Pressure in Young Adult Men.* American Journal of Cardiology, 2016. **117**(3): p. 394-8.
2. Kim, K. and S.M. Park, *Association of muscle mass and fat mass with insulin resistance and the prevalence of metabolic syndrome in Korean adults: a cross-sectional study.* Scientific Reports, 2018. **8**(1): p. 2703.
3. Kang, M.K., et al., *Association of low skeletal muscle mass with advanced liver fibrosis in patients with non-alcoholic fatty liver disease.* Journal of Gastroenterology & Hepatology, 2019. **34**(9): p. 1633-1640.
4. Kawanabe, S., et al., *Association of the muscle/fat mass ratio with insulin resistance in gestational diabetes mellitus.* Endocrine Journal, 2019. **66**(1): p. 75-80.
5. Yeung, C.H.C., et al., *Lean mass, grip strength and risk of type 2 diabetes: a bi-directional Mendelian randomisation study.* Diabetologia, 2019. **62**(5): p. 789-799.
6. Pacifico, L., et al., *Nonalcoholic Fatty Liver Disease Is Associated With Low Skeletal Muscle Mass in Overweight/Obese Youths.* Frontiers in Pediatrics, 2020. **8(no pagination)**.
7. Vaziri, Y., et al., *Lean Body Mass as a Predictive Value of Hypertension in Young Adults, in Ankara, Turkey.* Iranian Journal of Public Health, 2015. **44**(12): p. 1643-54.
8. Tershakovec, A.M., et al., *Body composition and metabolic factors in obese children and adolescents.* Int J Obes Relat Metab Disord, 2003. **27**(1): p. 19-24.
9. Fabea, L., et al., *Body composition is related to cognitive function among young adults in Ghana.* BMC Psychol, 2024. **12**(1): p. 114.
10. Visser, A.W., et al., *The role of fat mass and skeletal muscle mass in knee osteoarthritis is different for men and women: the NEO study.* Osteoarthritis & Cartilage, 2014. **22**(2): p. 197-202.
11. Takase, M., et al., *Relationships of Fat Mass Index and Fat-Free Mass Index with Low-Density Lipoprotein Cholesterol Levels in the Tohoku Medical Megabank Community-Based Cohort Study.* Journal of Atherosclerosis & Thrombosis, 2024. **31**(6): p. 979-1003.
12. Anoop, S., et al., *High body fat and low muscle mass are associated with increased arterial stiffness in Asian Indians in North India.* Journal of Diabetes & its Complications, 2015. **29**(1): p. 38-43.
13. Cheng, J.J., L.J. Liang, and C.C. Lee, *Associations of appendicular lean mass and abdominal adiposity with insulin resistance in older adults: A cross-sectional study.* PLoS ONE [Electronic Resource], 2024. **19**(5): p. e0303874.

14. Kim, J.A., et al., *Proportion and Characteristics of the Subjects with Low Muscle Mass and Abdominal Obesity among the Newly Diagnosed and Drug-Naive Type 2 Diabetes Mellitus Patients.* Diabetes & Metabolism Journal, 2019. **43**(1): p. 105-113.
15. He, Y.Y., et al., *Associations of muscle mass and strength with new-onset diabetes among middle-aged and older adults: evidence from the China health and retirement longitudinal study (CHARLS).* Acta Diabetologica, 2024. **61**(7): p. 869-878.
16. Scott, D., et al., *Associations of Low Muscle Mass and the Metabolic Syndrome in Caucasian and Asian Middle-aged and Older Adults.* Journal of Nutrition, Health & Aging, 2016. **20**(3): p. 248-55.
17. Jhee, J.H., et al., *High muscle-to-fat ratio is associated with lower risk of chronic kidney disease development.* Journal of Cachexia, Sarcopenia and Muscle, 2020. **11**(3): p. 726-734.
18. Yasuoka, M., et al., *Joint impact of muscle mass and waist circumference on type 2 diabetes in Japanese middle-aged adults: The Circulatory Risk in Communities Study (CIRCS).* Journal Of Diabetes, 2020. **12**(9): p. 677-685.
19. Dai, H., et al., *Fat mass to fat-free mass ratio and the risk of non-alcoholic fatty liver disease and fibrosis in non-obese and obese individuals.* Nutrition and Metabolism, 2021. **18(1) (no pagination)**.
20. Chargi, N., et al., *Cut-off values for low skeletal muscle mass at the level of the third cervical vertebra (C3) in patients with head and neck cancer.* Quantitative Imaging in Medicine and Surgery, 2022. **12(6)**: p. 3024-3033.
21. Mai, Z., et al., *Association between the skeletal muscle mass to visceral fat area ratio and metabolic dysfunction-associated fatty liver disease: A cross-sectional study of NHANES 2017-2018.* Journal Of Diabetes, 2024. **16**(6): p. e13569.
22. Guerra Valencia, J., et al., *The Association Between Lean-to-Fat Mass Ratio and Cardiometabolic Abnormalities: An Analytical Cross-Sectional Study.* Journal of Clinical Medicine Research, 2024. **16**(2-3): p. 81-93.
23. Lee, M.J., et al., *Age-Related Decrease in Skeletal Muscle Mass Is an Independent Risk Factor for Incident Nonalcoholic Fatty Liver Disease: A 10-Year Retrospective Cohort Study.* Gut & Liver, 2019. **13**(1): p. 67-76.
24. Knowles, R., et al., *Associations of Skeletal Muscle Mass and Fat Mass With Incident Cardiovascular Disease and All-Cause Mortality: A Prospective Cohort Study of UK Biobank Participants.* Journal of the American Heart Association, 2021. **10**(9): p. e019337.
25. Matsumoto, T., et al., *Associations of obstructive sleep apnea with truncal skeletal muscle mass and density.* Scientific Reports, 2018. **8**(1): p. 6550.

26. Gan, D., et al., *Low muscle mass and low muscle strength associate with nonalcoholic fatty liver disease.* Clinical Nutrition, 2020. **39**(4): p. 1124-1130.
27. Takegami, M., et al., *Relative low muscle mass and muscle strength is associated with the prevalence of metabolic syndrome in patients with type 2 diabetes.* Journal of Clinical Biochemistry and Nutrition, 2022. **71(2)**: p. 136-142.
28. Kim, T.N., et al., *Skeletal muscle mass to visceral fat area ratio is associated with metabolic syndrome and arterial stiffness: The Korean Sarcopenic Obesity Study (KSOS).* Diabetes Research & Clinical Practice, 2011. **93**(2): p. 285-291.
29. Guo, J., et al., *Fat-free mass index is a feasible predictor of insulin resistance in women with polycystic ovary syndrome: Evidence from a cross-sectional study.* Endocrine, 2024. **84**(2): p. 420-426.
30. Gong, H.J., et al., *Predicted lean body mass in relation to cognitive function in the older adults.* Frontiers in Endocrinology, 2023. **14(no pagination)**.
31. Jun, J.E., et al., *Additive effect of low skeletal muscle mass and abdominal obesity on coronary artery calcification.* European Journal of Endocrinology, 2021. **184**(6): p. 867-877.
32. Han, T.S., et al., *Associations of body fat and skeletal muscle with hypertension.* Journal of Clinical Hypertension, 2019. **21**(2): p. 230-238.
33. Song, Y.M. and K. Lee, *Comparison of the associations between appendicular lean mass adjustment methods and cardiometabolic factors.* Nutrition Metabolism & Cardiovascular Diseases, 2020. **30**(12): p. 2271-2278.
34. Kim, S.R., et al., *Changes in predicted lean body mass, appendicular skeletal muscle mass, and body fat mass and cardiovascular disease.* Journal of Cachexia, Sarcopenia and Muscle, 2022. **13**(2): p. 1113-1123.
35. Beijers, R., et al., *Normal Weight but Low Muscle Mass and Abdominally Obese: Implications for the Cardiometabolic Risk Profile in Chronic Obstructive Pulmonary Disease.* Journal of the American Medical Directors Association, 2017. **18**(6): p. 533-538.
36. Lee, J., et al., Skeletal Muscle Loss Is an Imaging Biomarker of Outcome after Definitive Chemoradiotherapy for Locally Advanced Cervical Cancer. Clinical Cancer Research, 2018. 24(20): p. 5028-5036.
37. Han, D.S., et al., Skeletal muscle mass adjusted by height correlated better with muscular functions than that adjusted by body weight in defining sarcopenia. Scientific Reports, 2016. 6: p. 19457.
38. Kim, T.N., et al., *Body size phenotypes and low muscle mass: the Korean sarcopenic obesity study (KSOS).* Journal of Clinical Endocrinology & Metabolism, 2013. **98**(2): p. 811-7.

39. Choubey, A., et al., *Association of Lean Body Mass and Fat Mass Index with Pulse Pressure Among Young Adults: A Cross Sectional Study.* International Journal of Tropical Medicine, 2024. **19(2)**: p. 70-73.
40. Iritani, S., et al., *Skeletal muscle depletion is an independent prognostic factor for hepatocellular carcinoma.* Journal of Gastroenterology, 2015. **50**(3): p. 323-32.
41. Frisoli, A., Jr., et al., *The association between low lean mass and osteoporosis increases the risk of weakness, poor physical performance and frailty in Brazilian older adults: data from SARCOS study.* European journal of clinical nutrition, 2021. **75**(3): p. 446-455.
42. Rier, H.N., et al., *Low muscle attenuation is a prognostic factor for survival in metastatic breast cancer patients treated with first line palliative chemotherapy.* Breast, 2017. **31**: p. 9-15.
43. Bi, L.N., et al., *Prevalence and influencing factors of muscle mass loss in adults with diabetes and a high body fat percentage: A cross-sectional study.* Journal of Back & Musculoskeletal Rehabilitation, 2024. **37**(1): p. 175-181.
44. Dabak, M.R., et al., *Evaluation of muscle mass in obesity, prediabetes and diabetes mellitus by different equations used for the measurement of muscle mass.* Diabetes & Metabolic Syndrome, 2019. **13**(3): p. 2148-2151.
45. Spahillari, A., et al., *The association of lean and fat mass with all-cause mortality in older adults: The Cardiovascular Health Study.* Nutrition Metabolism & Cardiovascular Diseases, 2016. **26**(11): p. 1039-1047.
46. Aoyama, T., et al., *Loss of Lean Body Mass as an Independent Risk Factor for Continuation of S-1 Adjuvant Chemotherapy for Gastric Cancer.* Annals of Surgical Oncology, 2015. **22**(8): p. 2560-6.
47. Abaza, H., et al., *Association between muscle mass and overall survival among colorectal cancer patients at tertiary cancer center in the Middle East.* Scientific Reports, 2024. **14**(1): p. 20836.
48. Park, J., et al., *Visceral adiposity and skeletal muscle mass are independently and synergistically associated with left ventricular structure and function: the Korean Genome and Epidemiology Study.* International Journal of Cardiology, 2014. **176**(3): p. 951-5.
49. Batsis, J.A., et al., *Low Lean Mass With and Without Obesity, and Mortality: Results From the 1999-2004 National Health and Nutrition Examination Survey.* J Gerontol A Biol Sci Med Sci, 2017. **72**(10): p. 1445-1451.
50. Faron, A., et al., *Fat-free muscle area measured by magnetic resonance imaging predicts overall survival of patients undergoing radioembolization of colorectal cancer liver metastases.* European Radiology, 2019. **29**(9): p. 4709-4717.

51. Low, S., et al., *Reduced skeletal muscle mass to visceral fat area ratio is independently associated with reduced cognitive function in type 2 diabetes mellitus.* Journal of Diabetes & its Complications, 2024. **38**(2): p. 108672.
53. Kouvari, M., et al., *Skeletal muscle mass in acute coronary syndrome prognosis: Gender-based analysis from Hellenic Heart Failure cohort.* Nutrition Metabolism & Cardiovascular Diseases, 2019. **29**(7): p. 718-727.
54. Ding, L., et al., *Central Lean Mass Distribution and the Risks of All-Cause and Cause-Specific Mortality in 40,283 UK Biobank Participants.* Obesity facts, 2024: p. 1-11.
55. Hansen, R.D. and B.J. Allen, *Fat-free mass components in active vs sedentary females aged 55-75 yr.* Applied Radiation & Isotopes, 1998. **49**(5-6): p. 735-6.
56. Bufano, A., et al., *New specific skeletal muscle mass index cut-offs for the assessment of sarcopenia in patients with severe obesity.* Frontiers in Endocrinology, 2024. **15(no pagination)**.
57. Jeznach-Steinhagen, A., et al., *Higher Muscle Mass and Higher Serum Prealbumin Levels Are Associated with Better Survival in Hemodialysis Patients during a Five-Year Observation Period.* Nutrients, 2023. **15(5) (no pagination)**.
58. Kim, T.N., et al., *The association of low muscle mass with soluble receptor for advanced glycation end products (sRAGE): The Korean Sarcopenic Obesity Study (KSOS).* Diabetes/Metabolism Research Reviews, 2018. **34**(3): p. 03.
59. Srikanthan, P., T.B. Horwich, and C.H. Tseng, *Relation of Muscle Mass and Fat Mass to Cardiovascular Disease Mortality.* American Journal of Cardiology, 2016. **117**(8): p. 1355-60.
60. Gotsman, I., et al., *Increased estimated fat-free mass and fat mass associated with improved clinical outcome in heart failure.* European Journal of Clinical Investigation, 2022. **52**(1): p. e13655.
61. Lee, S.Y., et al., *Low Skeletal Muscle Mass in the Lower Limbs Is Independently Associated to Knee Osteoarthritis.* PLoS ONE [Electronic Resource], 2016. **11**(11): p. e0166385.
62.
63. Qu, Q., et al., *Low lean mass with obesity in older adults with hypertension: prevalence and association with mortality rate.* BMC Geriatrics, 2023. **23**(1): p. 619.
64. Kiss, N., et al., *Low muscle mass, malnutrition, sarcopenia, and associations with survival in adults with cancer in the UK Biobank cohort.* JOURNAL OF CACHEXIA SARCOPENIA AND MUSCLE, 2023. **14**(4): p. 1775-1788.
65. Krachler, B., et al., *Cardiopulmonary fitness is a function of lean mass, not total body weight: The DR's EXTRA study.* European Journal of Preventive Cardiology, 2015. **22**(9): p. 1171-9.

66. Kim, D., et al., *Association of low muscle mass and obesity with increased all-cause and cardiovascular disease mortality in US adults.* Journal of Cachexia, Sarcopenia and Muscle, 2024. **15**(1): p. 240-254.
67. Madeira, E., et al., *Lean mass as a predictor of bone density and microarchitecture in adult obese individuals with metabolic syndrome.* Bone, 2014. **59**: p. 89-92.
68. Huang, B.T., et al., *Lean mass index, body fat and survival in Chinese patients with coronary artery disease.* Qjm, 2015. **108**(8): p. 641-7.
69. Kim, T.N., et al., *Association of low muscle mass and combined low muscle mass and visceral obesity with low cardiorespiratory fitness.* PLoS ONE [Electronic Resource], 2014. **9**(6): p. e100118.
70. O'Connor, D.P., et al., *Generalized equations for estimating DXA percent fat of diverse young women and men: the TIGER study.* Med Sci Sports Exerc, 2010. **42**(10): p. 1959-65.
71. Rutten, I.J., et al., *The influence of sarcopenia on survival and surgical complications in ovarian cancer patients undergoing primary debulking surgery.* Eur J Surg Oncol, 2017. **43**(4): p. 717-724.
72. Pickl, C., et al., *Body Composition as a Comorbidity-Independent Predictor of Survival following Nephroureterectomy for Urothelial Cancer of the Upper Urinary Tract.* Cancers (Basel), 2023. **15**(2).
73. Batista, G.A., et al., *Body composition, resting energy expenditure and inflammatory markers: impact in users of depot medroxyprogesterone acetate after 12 months follow-up.* Arch Endocrinol Metab, 2017. **61**(1): p. 70-75.
74. Larsson, B., et al., *Obesity, adipose tissue distribution and health in men--the study of men born in 1913.* Appetite, 1989. **13**(1): p. 37-44.
75. Sergi, G., et al., Assessing appendicular skeletal muscle mass with bioelectrical impedance analysis in free-living Caucasian older adults. Clinical Nutrition, 2015. 34(4): p. 667-73.
76. Cai, X.J., et al., *Cardiovascular effects of weight loss in old adults with overweight/obesity according to change in skeletal muscle mass.* JOURNAL OF CACHEXIA SARCOPENIA AND MUSCLE, 2024. **15**(1): p. 342-351.
77. Dulloo, A.G., et al., *Passive and active roles of fat-free mass in the control of energy intake and body composition regulation.* Eur J Clin Nutr, 2017. **71**(3): p. 353-357.
78. Cao, X., Shen, G., & Sun, T. (2026). Association Between Body Composition and Risk of Type 2 Diabetes Mellitus: A Systematic Review and Meta-Analysis. Diabetes, metabolic syndrome and obesity : targets and therapy, 19, 598731. https://doi.org/10.2147/DMSO.S598731

79. Hukportie, D.N., et al., *Association of Predicted Lean Body Mass and Fat Mass With Incident Diabetic Nephropathy in Participants With Type 2 Diabetes Mellitus: A Post Hoc Analysis of ACCORD Trial.* Frontiers in Endocrinology, 2021. **12**: p. 719666.

80. Batsis, J. A., & Villareal, D. T. (2018). Sarcopenic obesity in older adults: aetiology, epidemiology and treatment strategies. Nature reviews. Endocrinology, 14(9), 513–537. https://doi.org/10.1038/s41574-018-0062-9

81. Kim, B., et al., Changes in lower extremity muscle mass and muscle strength after weight loss in obese men: A prospective study. Obes Res Clin Pract, 2015. 9(4): p. 365-73.

82. Cai, C., Song, X., Chen, Y., Chen, X., & Yu, C. (2020). Relationship between relative skeletal muscle mass and nonalcoholic fatty liver disease: a systematic review and meta-analysis. *Hepatology international*, *14*(1), 115–126. https://doi.org/10.1007/s12072-019-09964-1

83. Cai, J., Yue, J., Lu, N., Li, S., Zheng, J., Huang, R., Jiang, Y., Shan, C., Liu, W., Ma, J., & Wang, L. (2024). Association of Fat Mass and Skeletal Muscle Mass with Cardiometabolic Risk Varied in Distinct PCOS Subtypes: A Propensity Score-Matched Case-Control Study. *Journal of clinical medicine*, *13*(2), 483. https://doi.org/10.3390/jcm13020483

84. Ramsey, R., E. Isenring, and L. Daniels, *Comparing measures of fat-free mass in overweight older adults using three different bioelectrical impedance devices and three prediction equations.* Journal of Nutrition, Health & Aging, 2012. **16**(1): p. 26-30.

85. Carbone, S., et al., *Lean Mass Abnormalities in Heart Failure: The Role of Sarcopenia, Sarcopenic Obesity, and Cachexia.* Curr Probl Cardiol, 2020. **45**(11): p. 100417.
